# Widespread Epigenetic Abnormalities Suggest a Broad DNA Methylation Erasure Defect in Abnormal Human Sperm

**DOI:** 10.1371/journal.pone.0001289

**Published:** 2007-12-12

**Authors:** Sahar Houshdaran, Victoria K. Cortessis, Kimberly Siegmund, Allen Yang, Peter W. Laird, Rebecca Z. Sokol

**Affiliations:** 1 Department of Biochemistry and Molecular Biology, Keck School of Medicine, University of Southern California, Los Angeles, California, United States of America; 2 Department of Preventive Medicine, Keck School of Medicine, University of Southern California, Los Angeles, California, United States of America; 3 Department of Surgery, Keck School of Medicine, University of Southern California, Los Angeles, California, United States of America; 4 Department of Obstetrics and Gynecology, Keck School of Medicine, University of Southern California, Los Angeles, California, United States of America; 5 Department of Medicine, Keck School of Medicine, University of Southern California, Los Angeles, California, United States of America; Texas A&M University, United States of America

## Abstract

**Background:**

Male-factor infertility is a common condition, and etiology is unknown for a high proportion of cases. Abnormal epigenetic programming of the germline is proposed as a possible mechanism compromising spermatogenesis of some men currently diagnosed with idiopathic infertility. During germ cell maturation and gametogenesis, cells of the germ line undergo extensive epigenetic reprogramming. This process involves widespread erasure of somatic-like patterns of DNA methylation followed by establishment of sex-specific patterns by *de novo* DNA methylation. Incomplete reprogramming of the male germ line could, in theory, result in both altered sperm DNA methylation and compromised spermatogenesis.

**Methodology/Principal Finding:**

We determined concentration, motility and morphology of sperm in semen samples collected by male members of couples attending an infertility clinic. Using MethyLight and Illumina assays we measured methylation of DNA isolated from purified sperm from the same samples. Methylation at numerous sequences was elevated in DNA from poor quality sperm.

**Conclusions:**

This is the first report of a broad epigenetic defect associated with abnormal semen parameters. Our results suggest that the underlying mechanism for these epigenetic changes may be improper erasure of DNA methylation during epigenetic reprogramming of the male germ line.

## Introduction

Approximately five million women in the United States reported difficulty in achieving a pregnancy in comprehensive surveys conducted by the CDC and National Survey of Family Growth from 1982 until 1995 [1]–[3], indicating that ten to twenty percent of couples attempting pregnancy are infertile. Preliminary follow-up data for 1990–2002 confirm this percentage [4]–[6]. Male factor infertility accounts for 40–50% of this impaired fecundity [7]. Well defined causes of male-factor infertility include congenital and acquired dysfunction of the hypothalamic-pituitary-testicular endocrine axis, anatomic defects, chromosomal abnormalities, and point mutations [8]–[10]. However, these diagnoses account for only a small proportion of cases, and etiology remains unknown for most male-factor infertility patients [7], [11].

Abnormal epigenetic programming of the germ line is proposed as a possible mechanism compromising fertility of some men currently diagnosed with idiopathic infertility. The mammalian germ line undergoes extensive epigenetic reprogramming during development and gametogenesis. In males, dramatic chromatin remodeling occurs during spermatogenesis [12], [13], and widespread erasure of DNA methylation followed by *de novo* DNA methylation occurs developmentally in two broad waves [12], [14]–[17]. The first occurs before emergence of the germ line, establishing a pattern of somatic-like DNA hypermethylation in cells of the pre-implantation embryo that are destined to give rise to all cells of the body, including germ cells. The second widespread occurrence of erasure takes place uniquely in primordial germ cells. Subsequent *de novo* methylation occurs during germ cell maturation and spermatogenesis, establishing a male germ line pattern of DNA methylation that remains hypomethylated compared with somatic cell DNA [14], [18]–[22]. Disruption of one or more of these epigenetic processes may lead to abnormal spermatogenesis and compromised sperm function.

A small number of studies have addressed the epigenetic state of the human male germ line. Substantial variation in DNA methylation profiles is reported in ejaculated sperm of young, apparently healthy men. Notable distinctions were observed both between samples from separate men and among individually assayed sperm from the same man [23]. Although this variation suggests that DNA methylation may be used as a biomarker of sperm quality, semen quality and fertility were not assessed in this study [23].

Several previous studies did assess sperm DNA methylation together with either sperm quality or fertility outcomes. However, measures of DNA methylation were limited, consisting of either immunostain–a single and somewhat nonspecific measure [24]–or sequence specific measures made at only one or two imprinted genes–a rare and specialized subset of DNA methylation targets [25]–[27].

To assess sperm DNA methylation at a more representative set of targets, we selected a much larger set of sequence-specific assays for use in the present study. We measured DNA methylation in ejaculated spermatozoa, interrogating sequences in repetitive elements, promoter CpG islands, and differentially methylated regions (DMRs) of imprinted genes. Then, to address the possible role of epigenetic programming in abnormal human spermatogenesis, we related sequence-specific levels of DNA methylation to standard measures of sperm quality. This is the first study to describe the epigenetic state of abnormal human sperm using an extensive panel of DNA methylation assays.

## Results

Standard semen analysis was conducted on samples collected by 69 men during clinical evaluation of couples with infertility. Among the 69 samples, semen volume ranged from 0.5 to 7.8 ml; total count 0 to 864 million sperm; total motile count 0 to 396 million sperm; and percentage normal sperm forms 0 to 26. Four samples were found to be azoospermic and excluded from subsequent analysis of DNA methylation.

**Figure 1:**
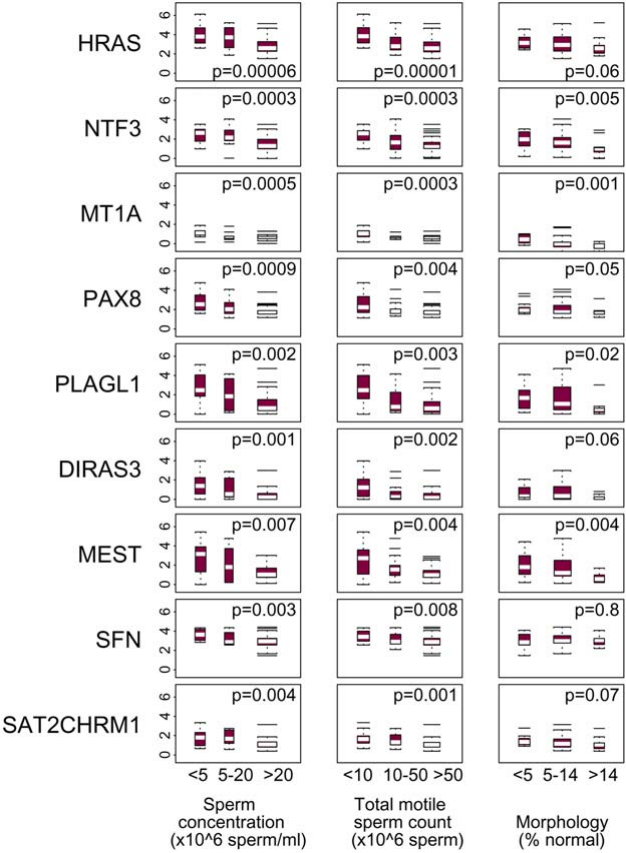
Box plots illustrating associations between semen parameters and level of methylation (PMR, on the natural-log scale) in DNA isolated from 65 study sperm samples. DNA methylation was measured by MethyLight. Methylation targets were sequences specific to the genes *HRAS, NTF3, MT1A, PAX8, PLAGL1, DIRAS3*, *MEST* and *SFN* and the repetitive element Satellite 2 (SAT2CHRM1). P-value for trend over category of semen parameter is given for each plot. Rows: DNA methylation targets; columns: semen parameters.

We evaluated 294 MethyLight reactions (Table S1A–B) for the presence of methylation in sperm DNA from an anonymous semen sample obtained from a sperm bank. The 35 selected reactions (Table S1A) were used to assay sperm DNA from 65 study samples. At many of the 35 sequences methylation levels were elevated in DNA from poor quality sperm. Striking associations with each of sperm concentration, motility and morphology were observed for four sequences: *NTF3*, *MT1A, PAX8* and *PLAGL1* (Figure 1).

*PLAGL1* is maternally imprinted. Our MethyLight assay for this gene interrogates a differentially methylated CpG island [28]. To determine whether other maternally imprinted genes are methylated in abnormal sperm, we used MethyLight to interrogate the differentially methylated sequence of *DIRAS3*. At this sequence we also observed greater DNA methylation in samples with poorer semen parameters (Figure 1). These results appeared to conflict with those of Marques et al [26] who reported no association between low sperm count and methylation of a DMR in a third maternally imprinted gene, *MEST*. We therefore used MethyLight to assess the methylation status of a differentially methylated *MEST* sequence investigated by these authors [26], and found elevated DNA methylation to be significantly associated with poor semen parameters (Figure 1), in agreement with our *PLAGL1* and *DIRAS3* results.

After correction for multiple comparisons, estimated associations between results of each of the 37 MethyLight assays and sperm concentration were significant for *HRAS*, *NTF3*, *MT1A, PAX8, DIRAS3*, *PLAGL1, SFN*, SAT2CHRM1 and *MEST* (Table 1, Figure 1).

**Table 1:** Trend p-values for associations between MethyLight results and semen parameters (see Methods).

MethyLight Reaction	Parameter of Standard Semen Analysis		
	Concentration	Motility	Morphology
*HRAS.HB.144	0.00006	0.00001	0.06265
*NTF3.HB.251	0.00029	0.00026	0.00464
MT1A.HB.205	0.00048	0.00026	0.00119
*PAX.8.HB.212	0.00086	0.00405	0.05143
*DIRAS3.HB.043	0.00109	0.00159	0.06016
*PLAGL1.HB.199	0.00213	0.00255	0.01951
*SFN.HB.174	0.00307	0.00804	0.79899
*SAT2CHRM1.HB.289	0.00448	0.00109	0.06793
*MEST.HB.493	0.00711	0.00373	0.00359
RNR1.HB.071	0.02	0.04	0.89
CYP27B1.HB.223	0.02	0.05	0.10
MADH3.HB.053	0.09	0.15	0.35
BDNF.HB.257	0.11	0.05	0.26
PSEN1.HB.263	0.16	0.27	0.81
CGA.HB.237	0.23	0.34	0.93
SERPINB5.HB.208	0.23	0.64	0.80
ICAM1.HB.076	0.24	0.29	0.05
MINT1.HB.161	0.24	0.60	0.34
PTPN6.HB.273	0.24	0.09	0.08
ALU.HB.296	0.25	0.29	0.87
CYP1B1.HB.239	0.28	0.42	0.61
SP23.HB.301	0.28	0.48	0.48
IFNG.HB.311	0.33	0.22	0.93
C9.HB.403	0.37	0.35	0.89
GP2.HB.400	0.41	0.39	0.94
GATA4.HB.325	0.45	0.20	0.12
UIR.HB.189	0.48	0.47	0.70
TFF1.HB.244	0.48	0.96	0.93
LDLR.HB.219	0.51	0.39	0.11
SASH1.HB.085	0.51	0.15	0.15
ABCB1.HB.051	0.54	0.27	0.16
HOXA10.HB.270	0.63	0.84	0.13
MTHFR.HB.058	0.70	0.38	0.43
LINE1.HB.330	0.87	0.47	0.14
LZTS1.HB.200	0.90	0.95	0.73
SMUG1.HB.086	0.90	0.36	0.76
‡IGF2.HB.345	0.91	0.71	0.11

**Figure 2:**
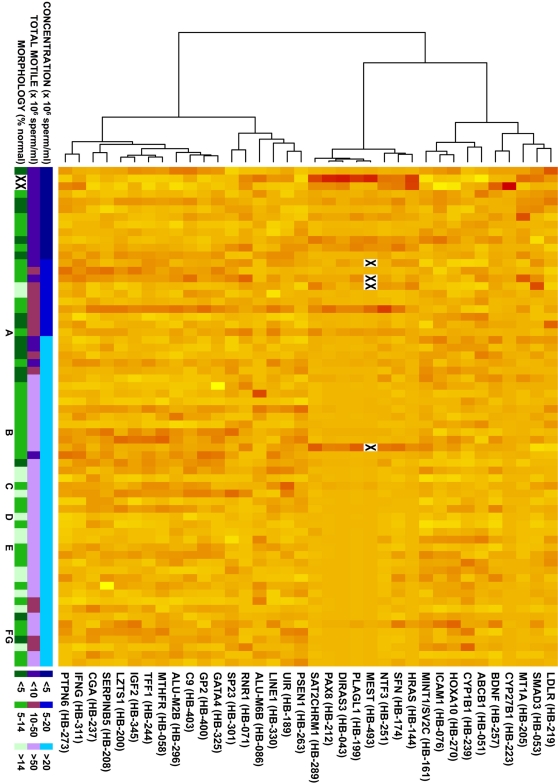
Cluster analysis of 36 MethyLight targets in 65 study sperm DNA samples. Left: dendrogram defining clusters; rows: 35 methylation targets; columns: 65 study samples ordered left to right on sperm concentration (samples A–G were also included in Illumina analyses (see Figure 3)) with poor to good concentration (blue), motility (purple), and morphology (green) represented by darkest to lightest hue; body of figure: standardized PMR values represented lowest to highest as yellow to red. X = missing.

* Belongs to cluster 2 (see Figure 2).

‡ Assay interrogates a non-differentially methylated sequence.

Trends were assessed over the following categories of semen parameters [N = number samples in category]: Concentration (<5 [N = 12], 5–20 [N = 10], >20 [N = 43]×10^6^ sperm per ml), Total motile sperm count (<10 [N = 18], 10–50 [N = 14], >50 [N = 33]×10^6^ sperm), Morphology (<5% [N = 15], 5–14% [N = 35], >14% [N = 13] normal sperm).

We then subjected MethyLight data from 36 of the assays to unsupervised cluster analysis. (Data for *SASH1* were not included, because methylation at this sequence was detected in only one sample.) This analysis identified three distinct clusters of sequences based on DNA methylation profiles in the 65 samples (Figure 2). Notably, the middle cluster shown in Figure 2 includes eight of the nine sequences (all except *MT1A*) individually associated with semen parameters. This middle cluster includes not only three sequences that are differentially methylated on imprinted loci, but also four single copy sequences specific to non-imprinted genes, and a repetitive element, Satellite 2 [29] (reaction named SAT2CHRM1). This result indicates that sperm abnormalities may be associated with a broad epigenetic defect of elevated DNA methylation at numerous sequences of diverse types, rather than a defect of imprinting alone as previously suggested [26].

**Figure 3:**
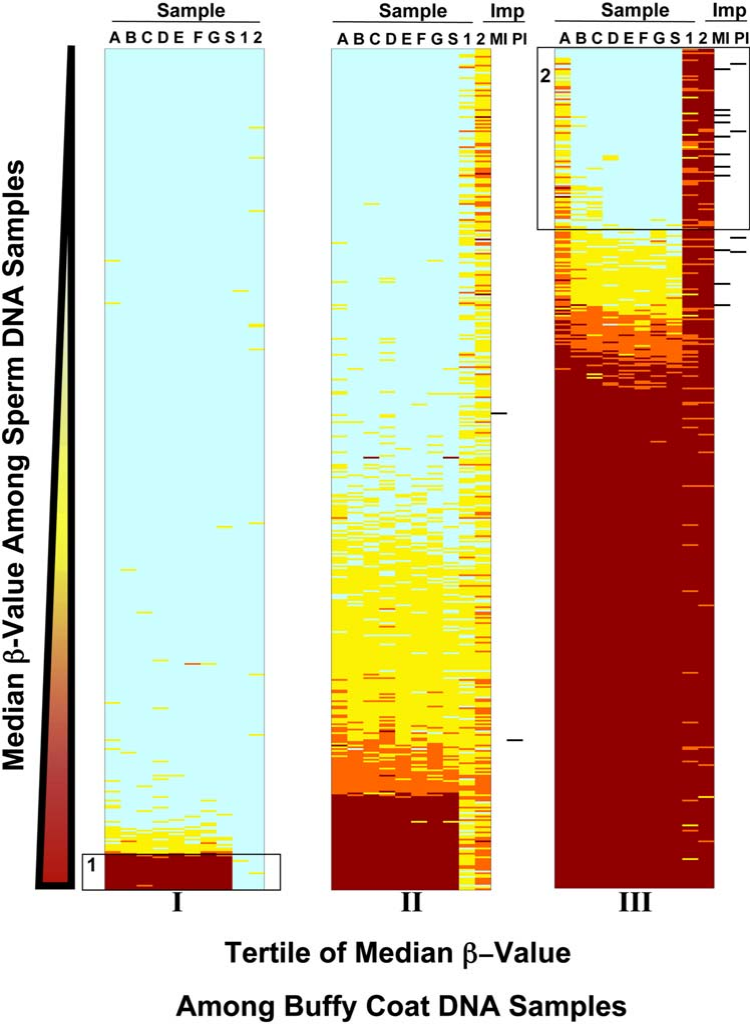
Results of Illumina analysis of 1,421 autosomal sequences in DNA isolated from sperm and buffy coat. Seven study sperm samples (A–G; with values of sperm concentration (10^6 ^sperm/ml) A:20, B:56, C:62, D:67, E:75, F:94, G:95), screening sperm sample (S), two buffy coat samples (1–2). Level of DNA methylation scored as β-value. Color: β-value for column sample at row sequence (green: β<0.1; yellow: 0.1≤β≤0.25; orange 0.25<β≤0.5; red: β>0.5). MI and PI: maternally and paternally imprinted genes (black bar). Sequences assigned to tertile of median β-value among buffy coat DNA samples (I, II, III) and sorted within tertile on median β-value among sperm DNA samples. Box 1: sequences with sperm-specific DNA methylation; Box 2: sequences with buffy coat-specific DNA methylation.

To learn more about the possible extent of this apparent defect, we used the Illumina® platform to conduct DNA methylation analysis of 1,421 sequences in autosomal loci. We included in this analysis DNA from the anonymous sperm sample used in the MethyLight screen (Figure 3, column S), two purchased samples of buffy coat DNA allowing us to observe methylation patterns in somatic cells (Figure 3, columns 1–2), and seven study sperm DNA samples remaining after MethyLight analysis (Figures 2–3, columns A–G).

Results of Illumina analyses appear in Figure 3. A large number of genes were similarly methylated in both sperm DNA and buffy coat DNA (blue regions on the left bar, I; red regions on the right bar, III), while others tended to be more methylated in DNA isolated from only one of these cell types. Boxes enclose sequences for which we observed particularly strong patterns of cell type-specific methylation. Box 1 identifies 19 sequences with sperm-specific DNA methylation. At these sequences, methylation profiles of all DNA sperm samples (A–G, S) closely resemble one another and differ greatly from those of buffy coat DNA. Box 2 identifies 102 sequences with buffy coat-specific DNA methylation. This set is larger in number than the sperm-specific set, as expected, given that sperm DNA is reportedly hypomethylated compared with somatic cell DNA [20]. The buffy coat-specific set comprises 7.2% of the 1,421 sequences including the majority of DMRs associated with imprinted genes that are on the Illumina panel. At many buffy coat-specific sequences, DNA methylation notably was elevated in sample A that had been isolated from sperm with the lowest concentration among samples A–G. Methylation of sample A DNA is elevated (β>0.1) at 76 of the 102 sequences in box 2, including all 10 that are known DMRs associated with imprinted genes.

Several factors assure us that our observations did not arise from somatic cell contamination of separated sperm samples [27]. Somatic cells are far larger than sperm and readily identified by microscopic evaluation of semen samples. Even if somatic cells are present in the neat ejaculate, the Isolate® sperm separation technique is specifically designed to separate spermatozoa from somatic cells and miscellaneous debris [30]. Moreover, although microscopic evaluation of semen samples conducted before sperm separation identified white blood cells in five of the 65 neat semen samples, we found that excluding results on these five samples from statistical analyses had minimal effect on associations between DNA methylation and semen parameters (results not shown), and DNA from these samples were excluded from Illumina assays.

## Discussion

Our observations are consistent with a broad epigenetic abnormality of poor quality human sperm in which levels of DNA methylation are elevated at numerous sequences in several genomic contexts. Previous studies of DNA methylation in poor quality human sperm interrogated only imprinted loci, measuring methylation of sequences in only one or two genes [25]–[27].

In the only study addressing the relationship between DNA methylation and fertility outcomes, immunostaining was used to measure genome-wide levels of DNA methylation in samples of ejaculated sperm collected for conventional in vitro fertilization (IVF). No association was observed between sperm DNA methylation and either fertilization rate or embryo quality in 63 IVF cycles [24]. There was, however, a possible association with pregnancy rate after transfer of good quality embryos. Interpretation of these results is limited by both small sample size and the use of a single summary measure of genome-wide DNA methylation.

Because of the enormous number of methylation targets in the human genome, only sequence-specific measures of DNA methylation are expected to reveal variation at individual sites. These include millions of repetitive DNA elements for which methylation is postulated to silence parasitic and transposable activity. There are also large numbers of target sequences corresponding to single copy genes. Examples include thousands of promoter CpG islands for which methylation appears to mediate expression of genes in a tissue- and lineage-specific fashion, and DMRs associated with dozens of imprinted genes for which parent-of-origin DNA methylation marks are believed to mediate monoallelic expression in somatic cells.

Sequence-specific measures were used in three previous studies investigating the relationship between methylation of human sperm DNA and spermatogenesis [25]–[27]. One study assessed DNA from spermatogonia and spermatocytes microdissected from seminiferous tubules of biopsied testicular tissue with spermatogenic arrest. DNA profiles consistent with correctly established paternal imprints were reported in all samples [25].

In the remaining two studies, DNA profiles were measured at specific DMRs associated with each of two genes, one paternally and one maternally imprinted. The resulting profiles were related to concentration of ejaculated sperm, an indicator of sperm quality. One of these studies reported correctly erased maternal imprints and correctly established paternal imprints in DNA from sperm of low concentration [27]. By contrast, the second reported that although maternal imprinting of *MEST* was correctly erased in DNA from sperm of low concentration, methylation at an *H19* sequence typically *de novo* methylated in spermatogenesis was incomplete in these samples [26]. No compelling explanation was offered for the apparently differing results of these studies. It is noteworthy, however, that each addressed sequences of only one or two imprinted genes, an extremely small and specialized subset of DNA methylation targets in the human genome. Data from these published studies could not, therefore, have revealed a disruption involving large numbers of genes, or shown that genes that are not imprinted are also affected. Our high-throughput analysis addressing hundreds of DNA methylation targets was far more likely to reveal such a defect.

Elevated DNA methylation could, in theory, arise from either *de novo* methylation or improper erasure of pre-existing methylation. Although we cannot rule out the possibility that processes responsible for *de novo* methylation are inappropriately activated in abnormal spermatogenesis, disruption of erasure seems a simpler mechanism. In mice, widespread erasure of DNA methylation has been shown to occur in both the pre-implantation embryo and again, uniquely, in primordial germ cells around the time that they enter the genital ridge. Several factors point to disruption of erasure in primordial germ cells as underlying the defect that we postulate. Primordial germ cells arise from cells of the proximal epiblast which have themselves embarked upon somatic development, as shown by expression of somatic genes [31], [32]. The germ cell lineage must therefore suppress the somatic program, which in mice is accomplished in part by genome-wide erasure of DNA methylation soon after germ cells migrate to the genital ridge [14], [33]–[41]. Incomplete erasure of DNA methylation at this stage of germ cell development has been postulated to explain transmission of variable phenotypes in several well characterized mouse models [42]–[44]. This erasure affects DNA methylation on single copy genes, imprinted genes and at least some repetitive elements [33], [34]. Therefore, its disruption could in theory result in the type of pattern we observe in poor quality sperm, with elevated levels of DNA methylation at DNA sequences of each of these types. Further, because this erasure is confined to primordial germ cells, we anticipate that its disruption would be compatible with normal somatic development.

In humans, primordial germ cells colonize the genital ridge at about 4.5 weeks of gestation. We are not aware of data describing DNA methylation in the human germ line at this date; however, the DMR in *MEST* at which we found elevated DNA methylation in poor quality sperm is reportedly unmethylated in the male germ line by week 24 of gestation [45]. We have not investigated potential causes of disrupted erasure. However, weeks 4.5–24 of gestation represent post-implantation stages of development wherein fetal physiology may be influenced by maternal factors and environmental compounds that cross the placenta. Possible origins of male infertility as early as 4.5 weeks of human gestation have not been studied. However, transient in vivo chemical exposure at 7–15 days post conception, which includes the analogous stage of murine development [34], [39], results in spermatogenic deficits in rats with grossly normal testes [46] and may be associated with elevated methylation of sperm DNA [47].

Taken together, the observations we report here suggest that epigenetic mechanisms may contribute to some cases of male factor infertility and that additional investigation of epigenetic mechanisms is warranted. Research relating sperm DNA methylation profiles to fertility outcomes is underway in our laboratory, and studies addressing pathophysiology associated with aberrant sperm DNA methylation may provide long-awaited mechanistic insights into abnormal sperm function. If, as we now postulate, improper erasure of DNA methylation in primordial germ cells results in an epigenetic defect of sperm, some categories of male factor infertility may be added to the growing list of diseases of adulthood that have fetal origins, and etiologic studies addressing events at this early stage of development will be needed.

## Materials and Methods

### Semen samples

Study semen samples were collected by 69 consecutive men ages 22–49 years who were partners of women undergoing evaluation for infertility at the Endocrine/Infertility Clinic of the Los Angeles County/University of Southern California Keck School of Medicine Medical Center. One additional semen sample was obtained from a sperm bank. The study was approved by the Institutional Review Board of the University of Southern California. Informed consent was not required because this research involved stored materials that had previously been collected solely for non-research purposes and were anonymous to the researchers/authors.

### Semen Analysis

Standard semen analysis was performed using WHO criteria and Strict Morphology as previously described [48], [49]. Semen volume, sperm concentration and motility, and leukocyte count were measured using the MicroCell chamber (Conception Technologies, San Diego, CA). Sperm morphology was assessed with the use of prestained slides (TestSimplets, Spectrum Technologies, Healdsburgh, CA), and percentage of morphologically normal sperm was documented. The samples were categorized according to concentration (<5, 5–20, >20 million sperm/ml), motility (<10, 10–50, >50 total motile sperm count (×10^6^)), and morphology (<5%, 5–14%, >14% normal) of sperm [48], [50]. Presence of any white blood cells, round cells, or epithelial cells was recorded. Following semen analysis, samples were stored at −30°C until processing for molecular analysis.

### Sperm Separation from Seminal Plasma

Semen samples were allowed to thaw at 37°C. Sperm were separated from seminal plasma using Isolate® Sperm Separation Medium (Irvine Scientific, Santa Ana, CA), a density gradient centrifugation column designed to separate cellular contaminants (including leukocytes, round cells, and miscellaneous debris) from spermatozoa [30]. Separation was performed according to the manufacturer's protocol [51], and the purity of separated sperm from contaminating cells was documented by light microscopy.

### DNA isolation

DNA was isolated from purified sperm as previously described [52], with 0.1X SSC added to the Lysis buffer, and samples incubated at 55°C over night or longer to complete the lysis procedure.

### Laboratory Analysis of DNA Methylation

Sodium bisulfite conversion was performed as previously described [29]. The amount of DNA in each aliquot was normalized, and a bisulfite-dependent, DNA methylation-independent control reaction was performed to confirm relative amounts of DNA in each sample.

MethyLight analyses were performed as previously described [29]. Reaction IDs and sequences of the primers and probes used in the 294 MethyLight reactions are provided in Table S1 (sections A–B). Thirty-five MethyLight reactions were selected for analysis of study sperm DNA samples based on cycle threshold (C(t)) values from analysis of the anonymous sample of sperm DNA. In brief, C(t) value is the PCR cycle number at which the emitted fluorescence is detectable above background levels. The C(t) value is inversely proportional to the amount of each methylated locus in the PCR reaction well, such that a low C(t) value suggests that the interrogated sequence is highly methylated. We interpreted C(t) values of 35 or less as an indication that a given sequence was methylated in the anonymous sample and selected 33 reactions on this basis. We included three additional reactions for which C(t) values slightly exceeded 35. Two (*CYP27B1* and *HOXA10*) were selected based on gene function potentially related to fertility, and one (a non-CpG island reaction for *IFNG*) based on prior observation of hypomethylation in tumor versus normal tissue (data not shown). When multiple reactions for a single locus resulted in C(t) values of less than 35, we selected only the reaction with the lowest C(t) value. Results of MethyLight analysis were scored as PMR values as previously defined [29].

Following MethyLight analyses, DNA remained from a subset of abnormal samples with greater sperm concentration. Illumina analysis was performed on sodium bisulfite converted sperm DNA of selected remaining samples, the anonymous semen sample, and purchased buffy coat DNA (HemaCare® Corporation, Van Nuys, CA) at the USC Genomics Core. Sodium bisulfite conversion for Illumina assay was performed using the EZ-96 DNA Methylation Kit (ZYMO Research) according to manufacturer's protocol. Illumina Methods and reagents are as previously described [53]. The primer names are listed in Table S2, identifying the 1,421 autosomal sequences on the *GoldenGate Methylation Cancer Panel I*, more fully described elsewhere [54], [55]. Results of Illumina assays were scored as β-values [53].

### Statistical association analyses of MethyLight data

Associations between the ranked MethyLight data and categorized semen values (Table 1) were tested using simple linear regression, with the semen characteristic categories scored as 0: low, 1: mid, 2: high. For selected sequences, boxplots of the methylation values (on the log(PMR+1) scale) are shown in Figure 1. The top and bottom of the box denote the 75^th^ and 25^th^ percentiles, and the white bar the median. Whiskers are drawn to the observation farthest from the box that lies within 1.5 times the distance from the top to the bottom of the box, with values falling outside the whiskers denoted as lines. Results of this analysis were included in Figure 1 for sequences associated with sperm concentration using the Benjamini and Hochberg procedure [56] to control the false discovery rate at 5%.

### Statistical cluster analysis of MethyLight data

Hierarchical cluster analysis of 36 loci was performed, using correlation to measure the distance between any two loci and Ward's method of linkage [57]. *SASH1* was omitted from the cluster analysis because only a single sample showed positive methylation. The 65 study samples were ordered from left to right by increasing semen concentration.

### Display of Illumina data

llumina data were displayed graphically in Figure 3 with results for study samples ordered left to right in columns by sperm concentration. Rows corresponding to each of the 1,421 sequences were divided into three tertiles of median β-value among buffy coat DNA samples (I, II, III), then sorted within tertile by median β-value among all sperm DNA samples. Box 1 contains all sequences within tertile I with median β-value among sperm DNA samples >0.5; box 2 contains all sequences within tertile III with median β-value among sperm DNA samples <0.1. Maternal or paternal imprinting status of each locus was scored according to the categorization of R. Jirtle [58]. All sequences specific to genes imprinted in humans were individually reviewed to determine whether they have been reported as belonging to a DMR for which parent of origin marks are maintained by DNA methylation [59]–[81]. Sequences meeting these criteria were scored as maternally imprinted (MI) or paternally imprinted (PI) with an indicator set for each on Figure 3.

## Supporting Information

Table S1 Primers, probes and reaction IDs for 294 MethyLight Assays; C. Group A: used in screening procedure and analysis of 65 study samples. Group B: used only in screening procedure. Group C: new assays designed to DMRs of maternally imprinted genes and used only in analysis of 65 study samples.
(0.10 MB PDF)

Table S2 Gene symbols, probe IDs, and measured β-values of Illumina analysis of 1,421 autosomal sequences. This panel is a subset of the *GoldenGate Methylation Cancer Panel I* described at www.illumina.com.Tertiles and heat map of methylation level of CpG loci are as shown in Figure 3. β-values of all loci for all samples are given. β-values of the heat map are as follows: Green, β<0.1; yellow, 0.1≤β≤0.25; orange, 0.25<β≤0.5; red, β>0.5. A to G represent 7 sperm samples selected from 65 study samples and are ordered from left to right from lowest to highest sperm concentration. S, Pre Screen sample; 1 and 2: Buffy Coat samples 1 and 2, respectively; Human maternal or paternal imprinted loci are indicated with a filled box: IMP, Imprinted; MI, Maternally Imprinted; PI, Paternally Imprinted.
(0.20 MB PDF)
