# Supplementary material for: Widespread Epigenetic Abnormalities Suggest a Broad DNA Methylation Erasure Defect in Abnormal Human Sperm
Source: PLoS One. 2007 Dec 12;2(12):e1289. doi: 10.1371/journal.pone.0001289 (PMC2100168; doi:10.1371/journal.pone.0001289)
Supplement: Table S1 — Primers, probes and reaction IDs for 294 MethyLight Assays; C. Group A: used in screening procedure and analysis of 65 study samples. Group B: used only in screening procedure. Group C: new assays designed to DMRs of maternally imprinted genes and used only in analysis of 65 study samples. (0.10 MB PDF) [file pone.0001289.s001.pdf]

Table S1. Primers, probes and reaction IDs for 294 MethyLight Assays; C(t) values of prescreen sperm DNA. Group A: used in screening procedure and analysis of 65 study samples. Group B: used only in screening procedure. Group C: new assays designed to DMRs of maternally imprinted genes and used only in analysis of 65 study samples.

GROUP	REACTION ID	GENE SYMBOL	PS Sample C(t) Value	Amplicon Location Start (UCSC Genome Coordinates)	Amplicon Location End (UCSC Genome Coordinates)	Forward primer Sequence	Reverse Primer Sequence	Probe Oligo Sequence
**A**	HB-189	UIR*	20.12	N/A	N/A	TTAGACGATGGGTGGTTAAGTAGAGAC	CCAACCGCCTACCTTAACCTC	6FAM-CTACCCGACCGCCACGCCG-BHQ-1
	HB-296	ALU	21.98	N/A	N/A	GGATTATAGGCGCGCGTTATT	TCACACCCGTAATCCGAACA	6FAM-ACCGAAACGAACGAATCACGAAATCAAA-BHQ-1
	HB-301	SP23	23.73	N/A	N/A	GGAGTTAATTTATGGTAGAGAGGATACGTTAT	AACCTTTAAAAACGCGAACCCTA	6FAM-CACCTTTCGCTACGACCCTTACGCTCA-BHQ-1
	HB-244	TFF1	26.23	42656349	42656489	ATGTTGATTAGAGTTTTTGTAGTTTTAAATGAT	CCGTTCGTAAAATCCCCTAATACTT	6FAM-TATCCTAATACCATCGACGTCCCTCCAAAAAA-BHQ-1
	HB-200	LZTS1	26.61	20154741	20154825	GCGGCGTTGTAGGGACG	CGCGCGCTAACTCTTCTACG	6FAM-ATTACCGCCTTTAAACTCCGAACCCTCCA-BHQ-1
	HB-263	PSEN1	27.12	72672585	72672701	TGTGATGTAGTTGGTTCGTAAATAGGTAT	AACCCCTATATAAATTAAACCCAACGTA	6FAM-ACTTCTCCCGACATACAAACGATAACCATCCT-BHQ-1
	HB-257	BDNF	27.14	27700880	27700949	AGTGGTTGCGTAAGTTGGGAA	TCATCCTTTCGCCTCCAAAT	6FAM-CTTTCCGCCAACACGTAACCTCTTCG-BHQ-1
	HB-237	CGA	27.18	87861458	87861547	GGGTTTTTTGTAGGATGTGTTTAGG	AACTACAATTACTAAAAACTCATAAAACGAAACT	6FAM-TCCCTCTTCGAATCCACAATCAACCG-BHQ-1
	HB-400	GP2	27.24	20246283	20246417	GATTTATTTTCGATGAGAATATAAAGCGT	CTCCCATCATACCTATACACCAATTAAA	6FAM-TTGTGGGTCGTTTTTTTA-MGBNFQ
	HB-085	SASH1	28.05	148705695	148705778	TAGTTCGTGGTTATTTAGATTCGAGGT	GCGCTACATACCCCGACG	6FAM-CCGCGAAAACCCTTCGCAAACG-BHQ-1
	HB-325	GATA4	28.31	11645081	11645162	TTACGTTGTGGCGTCGTAATG	CCACGTACCCCGTAAAACTTCA	6FAM-CGCAAACATTACACACAAACTCGCCCT-BHQ-1
	HB-208	SERPINB5	28.84	59295148	59295227	GAAAAGGAATAGGTAAGCGAGGAGT	ATAAACCACCGCTACTTCTACCCA	6FAM-CACGATCGCCTCCACATCCAAATCTTT-BHQ-1
	HB-403	C9	30.05	39400223	39400378	ATGTTTTATTAGATTTGTTTTGTTATTTTATTCGT	CAAACAAAAAAATAACTAACTCACCTAATCG	6FAM-TTAGTTTGTCGGAGTTTTGTAG-MGBNFQ
	HB-289	SAT2CHRM1	30.16	N/A	N/A	TCGAATGGAATTAATATTTAACGGAAAA	CCATTCGAATCCATTCGATAATTCT	6FAM-CGATTCCATTCGATAATTCCGTTT-MGBNFQ
	HB-086	ALU	30.20	N/A	N/A	GGATTATAGGCGCGCGTTATT	TCACACCCGTAATCCGAACA	6FAM-ACCGAAACGAACGAATCACGAAATCAAA-BHQ-1
	HB-273	PTPN6	30.23	6930354	6930436	GATGGCGGTGATAATTGTTATCG	ATCAAACAAAACATATAAACGCCATT	6FAM-AACACAACGCCCGACATCCAACAAA-BHQ-1
	HB-071	RNR1	30.67	N/A	N/A	CGTTTTGGAGATACGGGTCG	AAACAACGCCGAACCGAA	6FAM-ACCGCCCGTACCACACGCAAA-BHQ-1
	HB-058	MTHFR	30.89	11797288	11797377	TGGTAGTGAGAGTTTTAAAGATAGTTCGA	CGCCTCATCTTCTCCCGA	6FAM-TCTCATACCGCTCAAAATCCAAACCCG-BHQ-1
	HB-076	ICAM1	31.03	10242630	10242709	GGTTAGCGAGGGAGGATGATT	TCCCCTCCGAAACAAATACTACAA	6FAM-TTCCGAACTAACAAAATACCCGAACCGAAA-BHQ-1
	HB-239	CYP1B1	31.71	38215493	38215577	CGGCGTTATTTGGAGTGGTT	CCCGACAATACTTAAACACACAAAA	6FAM-CGCAACTAACCCTAAAATTTCCCGCGTAA-BHQ-1
	HB-051	ABCB1	32.01	86874884	86874962	TCGGGTCGGGAGTAGTTATTTG	CGACTATACTCAACCCACGCC	6FAM-ACGCTATTCCTACCCAACCAATCAACCTCA-BHQ-1
	HB-174	SFN	32.04	26874056	26874136	GAGGAGGGTTCGGAGGAGAA	ATCGCACACGCCCTAAAACT	6FAM-TCTCCCGATACTCACGCACCTCGAA-BHQ-1
	HB-330	LINE1	32.10	N/A	N/A	GGACGTATTTGGAAAATCGGG	AATCTCGCGATACGCCGTT	6FAM-TCGAATATTGCGTTTTCGGATCGGTTT-BHQ-1
	HB-212	PAX8	32.31	113709183	113709259	CGGGATTTTTTTGTCGTATTTGA	ACCTTTCCCCATACTACCTCCG	6FAM-ACGAACAATTCACGAACCAAACCCTCCT-BHQ-1
	HB-251	NTF3	32.47	5473982	5474055	TTTCGTTTTTGTATTTTATGGAGGATT	CCGTTTCCGCCGTAATATTC	6FAM-TCGCCACCACGAAACTACCCACG-BHQ-1
	HB-161	MINT1/SV2C**	33.06	75415734	75415861	GGGTTGAGGTTTTTTGTTAGCG	CCCCTCTAAACTTCACAACCTCG	6FAM-CTACTTCGCCTAACCTAACGCACAACAAACG-BHQ-1
	HB-205	MT1A	33.93	55229471	55229550	CGTGTTTTCGTGTTATTGTGTACG	CTCGCTATCGCCTTACCTATCC	6FAM-TCCACACCTAAATCCCTCGAACCCACT-BHQ-1
	HB-345	IGF2	34.04	N/A	N/A	TGACGAGGTTAGTGAGGGACGGC	CAAACTCCTCGATCCACCCAAAATAATATCTATAA	6FAM-AAAAATTCAC/TTTCCCCAAAAA-MGBNFQ
	HB-053	SMAD3	34.06	65145579	65145653	CGTGAAGCGTTTGTTGGGT	TTAACCGCCTTCTCGCACC	6FAM-TCCTCCTACCCGTTCTACTCGCCCTTCTT-BHQ-1
	HB-199	PLAGL1	34.15	1443711135	144371211	ATCGACGGGTTGAATGATAAATG	CTCGACGCAACCATCCTCTT	6FAM-ACTACCGCGAACGACAAAACCCACG-BHQ-1
	HB-144	HRAS	34.22	524232	524327	GAGCGATGACGGAATATAAGTTGG	CGTCCACAAAATAATTCTAAATCAACTAA	6FAM-CACTCTTACCCACACCGCCGACG-BHQ-1
	HB-219	LDLR	34.28	11060912	11061014	GATATCGGTTTTTTAATTCGTGAAGTT	TTCACCGAAAACCCAAATACAA	6FAM-ATCAAATCGCCTACCCTAACGACACTTTCG-BHQ-1
	HB-223	CYP27B1	35.07	56446731	56446808	GGGATAGTTAGAGAGAACGGATGTTT	CCGAATATAACCACACCGCC	6FAM-CCAACCTCAACTCGCCTTTTCCTTATTTCA-BHQ-1

	HB-270	HOXA10	35.16	26987339	26987422	TGTATTGATGGGTTAGGAGACGTATT	CCCACCAACCACGTTAAAACA	6FAM-CAACTCCCGACCTTCGAACCAAAATATCG-BHQ-1
	HB-311	IFNG	37.86	66839949	66840111	TGAAGAGTTAATATTTTATTAGGGCGAA	TTCCTTTAAACTCCTTAAATCCTTTAACG	6FAM-ACAAACCCATTATACCCACCTA-MGBNFQ
**B**	HB-145	TFF1	30.47	42659974	42660121	TAAGGTTACGGTGGTTATTTCGTGA	ACCTTAATCCAAATCCTACTCATATCTAAAA	6FAM-CCCTCCCGCCAAAATAAATACTATACTCACTACAAAA-BHQ-1
	HB-351	IGF2	34.05	N/A	N/A	GACGAGGTTAGTGAGGGACGGC	AACTCCTCGATCCACCCAAAATAATATCTATAA	6FAM-AAAAATTCAC/TTTCCCCAAAAA-MGBNFQ
	HB-346	IGF2	34.08	N/A	N/A	TTTGACGAGGTTAGTGAGGGACGGC	CCAAACTCCTCGATCCACCCAAAATAATATCTATAA	6FAM-AAAAATTCAC/TTTCCCCAAAAA-MGBNFQ
	HB-349	PTPN6	34.38	6931106	6931193	TTGGTTCGGTTTAGTCGTAAGAATTA	ATAACCAAAATACCCGAAATTACGAA	6FAM-CCCACCTACCTAACGAAAAACGAAAAATCACC-BHQ 1
	HB-348	PTPN6	35.09	6930726	6930844	TTAAGGGGTCGGTCGCGT	CAACTCCCAATACCGAACCG	6FAM-CTCACCACCCCCGATAATCCCAATTCTAAA-BHQ-1
	HB-072	ALU	36.12	N/A	N/A	AGGTCGAGGTCGGCGG	CCACGCCCGACTAATTTTATATCTT	6FAM-CAAACTAATCTCAAACTCCCGACCTCAAACGA-BHQ-1
	HB-302	HTRS	36.23	N/A	N/A	GGCGGTTTCGGAGTAAGTCG	TCGCAACATCTTACTACCTTCGAAA	6FAM-AAACACCGACCGAACTCTACTCCGCTCC-BHQ-1
	HB-306	TNFRSF10A	36.58	23138801	23138877	AGTTTTTGGTATTTAGTAGGCGTTCG	CAAACCCCGCAATAACCTCTATATC	6FAM-ATTCCGCCACCCATCCGTCCA-BHQ-1
	HB-061	S100A2	36.82	150354273	150354354	TGTTTGAGTCGTAAGTAGGGCGT	CGTATCATTACAATACCGACCTCCT	6FAM-ATCCTCCCTTTCTTATCCGCCAAACCCT-BHQ-1
	HB-332	IGF2	37.05	N/A	N/A	CGAGGTTAGTGAGGGACGGC	CCTCGATCCACCCAAAATAATATCTATAA	6FAM-AAAAATTCATTTCCCCAAAAA-MGBNFQ
	HB-242	ONECUT2	37.47	53253467	53253547	ACGGGCGTTAAGCGTAATTATTT	CCACAACCACTAATAACTTCCCGTA	6FAM-CCCGCCTCCCGAAACAACTACGA-BHQ-1
	HB-333	IGF2	38.01	N/A	N/A	CGAGGTTAGTGAGGGACGGC	CCTCGATCCACCCAAAATAATATCTATAA	6FAM-AAAAATTCACTTCCCCAAAAA-NFQMGB
	HB-395	2C64 / BC031882	38.04	47124419	47124488	TGTTTTTAGGGTTTTGGCGTTAAG	TACAAACGCGACCGCCA	6FAM-AAACGCCCAACGCGCGACTCTAAA-BHQ-1
	HB-262	PSEN1	38.06	72673243	72673319	GTCGGGTGGAGAGAGATTTCG	AACACCTACGCCCTAAAACGTC	6FAM- TCGAACAAACAACATTTCCGAACCAAAACT-BHQ-1
	HB-310	CLDN7	38.07	7106456	7106530	GGGTTGTAGGTTCGAGGTTGC	CACCTATCCGCCCGCAC	6FAM-CTAACTCACCTCCGAACCACCTCTACTACGCA-BHQ-1
	HB-269	CDKN2A	38.34	21961094	21961191	GCGTTCGAGTGGCGGA	CTCCCGAACAACGTCGTACAC	6FAM-CAATTAAACTCCGCGCCGTAAAACAACAA-BHQ-1
	HB-404	HDGF	39.02	153533994	153534099	GACGTCGTGTTCGTTAGGTGTTT	CTTATAAATCTCCGACTCTTTCTCTATCG	6FAM-TTCCAAACCCACCCGCCGATT-BHQ-1
	HB-193	COL1A2	39.70	93668865	93668953	CGGTAGTAGGAGGTTTCGGTTAAGT	CCTAAATCACCGACGAAAATATCA	6FAM-CGAACGCGAACATACAATCGTAACCAATACCT-BHQ
	HB-048	DNAJC15	40.06	42495746	42495826	TTTCGGGTCGTTTTGTTATGG	ACTACAAATACTCAACGTAACGCAAACT	6FAM-TCGCCAACTAAAACGATAACACCACGAACA-BHQ-1
	HB-202	LPHN2	40.07	81978452	81978551	GAGGATTTAGCGCGTAGTGAGTG	AATCCCCGAACTCTACCTCCA	6FAM-CCCATTAACACACCCATTCAACCGCTAA-BHQ-1
	HB-142	MBD2	40.11	50005060	50005138	AGGCGGAGATAAGATGGTCGT	CCCTCCTACCCGAAACGTAAC	6FAM-CGACCACCGCCTCTTAAATCCTCCAAA-BHQ-1
	HB-241	SYK	40.15	90643286	90643370	AGGGTCGTTGGGTGTTTGTG	AACATAAACCGCATCGATCCC	6FAM-CGCCAACGCGATAACTTCTATAACTACCCAA-BHQ-1
	HB-207	MT3	40.21	55180944	55181018	GGTTTTAGGGTTTATGTCGAGGAGA	CCGCGCGTCCAATTACTTA	6FAM-AAAACCCGTTCACCGCCTCCAACTACTA-BHQ-1
	HB-154	MYOD1	40.33	17697363	17697435	GAGCGCGCGTAGTTAGCG	TCCGACACGCCCTTTCC	6FAM-CTCCAACACCCGACTACTATATCCGCGAAA-BHQ-1
	HB-159	MGMT	40.68	131155782	131155872	CTAACGTATAACGAAAATCGTAACAACC	AGTATGAAGGGTAGGAAGAATTCGG	6FAM-CCTTACCTCTAAATACCAACCCCAAACCCG-BHQ-1
	HB-176	RARB	40.70	25444834	25444919	TTTATGCGAGTTGTTTGAGGATTG	CGAATCCTACCCCGACGATAC	6FAM-CTCGAATCGCTCGCGTTCTCGACAT-BHQ-1
	HB-312	IFNG	40.93	66839601	66839685	TTGGTTTAATTTTTTCGGAAACGA	CAACCAAAAAAACCCAAAACGAT	6FAM-AAACCAAAATATAACTTATATATTTCA-MGBNFQ
	HB-279	SFRP2	41.09	155068371	155068471	TTTATAATTTTGATTTTTTTACGGTATTGG	GAAACCGCCTCGACGAACT	6FAM-CTCGAATCTCCAACCACCGTTCAACAA-BHQ-1
	HB-258	BDNF	41.12	27678453	27678525	CGTATCGGGTTGGTTTTTTTGTT	CGCCCGCTCGCTATCC	6FAM-CCGTAACGCCTCGAACTCCCGA-BHQ-1
	HB-391	GPR88	41.14	100717612	100717678	CGCGGGTGTTTAGTTGCGT	GCGACGACCGCTACCGTTA	6FAM-CCGCCACAACCGTACCCGCAATATC-BHQ-1
	HB-210	PAX8	41.86	113752743	113752811	ATCGATCGGTTTTATTTCGTTGAG	ACCAATCCGCGACCTACG	6FAM-ACCTCGCCAAACCCATCTCCCAAAA-BHQ-1
	HB-052	PTTG1	42.07	159781430	159781499	GCGTTCGTTTATCGCGGT	CCGCGACCCTCCCATT	6FAM-ACTCACGCAAATCTTAACAACCGCATTCA-BHQ-1
	HB-393	LAMB1	42.08	107237637	107237723	GGCGGGCGGTCGTAAT	CGCAAATTCGACTTTCAAACAA	6FAM-AAAATAAAACCGAAACCTCACCCGATCCTC-BHQ-1
	HB-110	ERCC1	42.14	50618574	50618664	GGGCGAGTCGAAGGTGG	CTCCGAAAACTCCATAACGTCAA	6FAM-CCCAACGCTAAAAACTCTATAACGCCACG-BHQ-1
	HB-069	CADM1	43.02	114880288	114880369	GGGTTTCGGAGGTAGTTAACGTC	CACTAAAATCCGCTCGACAACAC	6FAM-ACACTCGCCATATCGAACACCTACCTCAAA-BHQ-1
	HB-065	PTGS2	43.03	183381471	183381545	CGGAAGCGTTCGGGTAAAG	AATTCCACCGCCCCAAAC	6FAM-TTTCCGCCAAATATCTTTTCTTCTTCGCA-BHQ-1
	HB-068	VDR	43.03	46585350	46585440	ACGTATTTGGTTTAGGCGTTCGTA	CGCTTCAACCTATATTAATCGAAAATACA	6FAM-CCCACCCTTCCTACCGTAATTCTACCCAA-BHQ-1
	HB-062	CLIC4	43.09	24817200	24817272	GGCGGTGTTGAGGAGTTGA	CCGATTCCCGCCGTACTAC	6FAM-CGCTAAACTATCCGAAATCGAACTAACCACG-BHQ-1
	HB-234	PITX2	44.06	111915835	111915919	GGAGTGACGTGACGTTAGTAGAGATTT	AACCGCGCAACCGAACT	6FAM-CGCCCGCGCGCCACTATACA-BHQ-1
	HB-278	SMAD6	44.08	64781526	64781629	ATGTTAGTTTAGATATTTTGGCGGTTTC	CGACCCTACAATAAAACGTATTCTCCT	6FAM-AAACCTTATTTACGCAACAATCAACGCCG-BHQ-1
	HB-103	RPA2	44.15	27925472	27925546	TGGCGCGAATTTGAGTACG	CGTATAATCCCACCCTCGTCA	6FAM-CGCGACTTCTACCGTCACTTCCTTTATTCG-BHQ-1
	HB-253	DRD2	44.16	112850580	112850649	GAAGTCGGAAATTTTGGTCGC	ATCTCGAAAAAACACTTCCCCC	6FAM-ACACCCAAACGCGAAACCCGAAACT-BHQ-1
	HB-254	GABRA2	44.39	46233296	46233369	TCGTCGGAGGAGCGGA	AACCTCTCGAAAACCCCAACA	6FAM-ACGACCTCGAAAAACAACCCGAAACTACG-BHQ-1
	HB-256	GAD1	44.66	171500487	171500569	CGATTGGTTCGGCGTAGAAA	CCCTCCGATATACAAAACCCC	6FAM-CCCGCACAACTCTCGCTTCTCTTTACAA-BHQ-1
	HB-327	GATA3	44.66	8136301	8136380	TGTATCGGGACGGAATCGTT	ACGCGCGCTCTAACCCTT	6FAM-AAATATAACCGCGACTCCTACCAATTCATTCG-BHQ-1
	HB-184	SEZ6L	45.06	24889734	24889836	GCGTTAGTAGGGAGAGAAAACGTTC	ATACCAACCGCCTCCTCTAACC	6FAM-CCGTCGACCCTACAAAATTTAACGCCA-BHQ-1

HB-040	CCND2	45.09	4252120	4252184	GGAGGGTCGGCGAGGAT	TCCTTTCCCCGAAAACATAAAA	6FAM-CACGCTCGATCCTTCGCCCG-BHQ-1
HB-259	NEUROD1	45.88	182370725	182370806	GTTTTTGCGTGGGCGAAT	CCGCGCTTAACATCACTAACTAAA	6FAM-CGCGCGACCACGACACGAAA-BHQ-1
HB-201	SFRP1	46.02	41286207	41286276	GAATTCGTTCGCGAGGGA	AAACGAACCGCACTCGTTACC	6FAM-CCGTCACCGACGCGAAAACCAAT-BHQ-1
HB-247	THBS1	46.52	37659935	37660009	GTTTTGAGTTGGTTTTACGTTCGTT	CGACGCACCAACCTACCG	6FAM-ACGCCGCGCTCACCTCCCT-BHQ-1
HB290	SATALPHACHRM1	47.48	N/A	N/A	TGATGGAGTATTTTTAAAATATACGTTTTGTAGT	AATTCTAAAAATATTCCTCTTCAATTACGTAAA	6FAM-TATCCCGTTTCCAACGAA-MGBNFQ
HB-268	HOXA1	47.90	26908602	26908684	TTGTTTATTAGGAAGCGGTCGTC	TCGAACCATAAAATTACAACTTTCCA	6FAM-TCGTACGCGATCAACGCCAACAATTA-BHQ-1
HB-248	TYMS	48.58	647871	647946	CGGCGTTAGGAAGGACGAT	TCTCAAACTATAACGCGCCTACAT	6FAM-CCGAATACCGACAAAATACCGATACCCGT-BHQ-1
HB-206	MT2A	48.99	55199620	55199708	GCGTTTTCGTCGTGTGTATAGTTT	TTCCCAAATCCCGCTTTCA	6FAM-CGCGCGCTAACGACTCAAATTCG-BHQ-1
HB-162	CACNA1G	49.10	45991745	45991815	GTCGTCGGCGTTATTTTAGAAAGTT	CACCGACGCCCAACACA	6FAM-ACGCTCCGCTCCCGAATACCCA-BHQ-1
HB-113	NDUFA12L	49.12	60277058	60277170	GGTTAAGGCGTTTAGAGTCGGG	TCATACGACACTTAAAATATCACCGAAA	6FAM-CCCTTCACTCTAACATCGAAACCCTACCCG-BHQ-1
HB-215	TFAP2A	49.57	10493365	10493490	CACCCCCATATACGCGCTAA	GGTCGTTACGTTTCGGGTAGTTTA	6FAM-CGCGCTCACACGCTCAAAAACCT-BHQ-1
HB-041	FHIT	None$^{\phi}$	61211898	61211972	GGCGCGGGTTTGGG	CGCCCCGTAAACGACG	6FAM-CACTAAACTCCGAAATAATAACCTAACGCGCG-BHQ-1
HB-042	SOCS1	None	11256473	11256558	GCGTCGAGTTCGTGGGTATTT	CCGAAACCATCTTCACGCTAA	6FAM-ACAATTCCGCTAACGACTATCGCGCA-BHQ-1
HB-043	DIRAS3	None	68228349	68228434	GCGTAAGCGGAATTTATGTTTGT	CCGCGATTTTATATTCCGACTT	6FAM-CGCACAAAAACGAAATACGAAAACGCAAA-BHQ-1
HB-044	RASSF1	None	50353155	50353219	ATTGAGTTGCGGGAGTTGGT	ACACGCTCCAACCGAATACG	6FAM-CCCTTCCCAACGCGCCCA-BHQ-1
HB-045	BRCA1	None	38530918	38531006	GAGAGGTTGTTGTTTAGCGGTAGTT	CGCGCAATCGCAATTTTAAT	6FAM-CCGCGCTTTTCCGTTACCACGA-BHQ-1
HB-046	DAPK1	None	87342485	87342552	TCGTCGTCGTTTCGGTTAGTT	TCCCTCCGAAACGCTATCG	6FAM-CGACCATAAACGCCAACGCCG-BHQ-1
HB-047	TWIST1	None	18929791	18929865	GTAGCGCGGCGAACGT	AAACGCAACGAATCATAACCAAC	6FAM-CCAACGCACCCAATCGCTAAACGA-BHQ-1
HB-049	DPH1	None	1880101	1880170	ACGCGGAGAGCGTAGATATTG	CCGCCCAACGAATATCCC	6FAM-CCCGCTAACCGATCGACGATCGA-BHQ-1
HB-050	CDH1	None	67328528	67328623	AGGGTTATCGCGTTTATGCG	TTCACCTACCGACCACAACCA	6FAM-ACTAACGACCCGCCCACCCGA-BHQ-1
HB-054	CXADR	None	17807103	17807169	TACGCGGTTGGAGAAGTCG	ATAAACTCGCGTCACTTCCGA	6FAM-AACGACCCGAACCGAACTACGAACG-BHQ-1
HB-059	CLDN1	None	191522936	191523032	CGGTGAGTCGTTTTGAAATCG	ACGCAAAACCGCTAAACGC	6FAM-GATTTAAAACAACTCCGCCCGCCTCA-BHQ-1
HB-060	PPARG	None	12304350	12304465	GCGTTCGCGTTCGTTTTC	CGCCCCAAACGACGAC	6FAM-CCCGCCTACCCGCGACGAAA-BHQ-1
HB-063	STAT1	None	191704255	191704343	GCGTAGGATTCGGAAGGGTTA	AACAAACCCCAAACCGAACA	6FAM-AACGACCCAACGCGCTCGAAAA-BHQ-1
HB-064	SORBS3	None	22479225	22479297	GTTTCGGTTGTCGTTGGGTT	ACGCCGACGAACTCTACGC	6FAM-ACGACGCAATCAAAACCCGCGA-BHQ-1
HB-066	HSD17B4	None	118816177	118816247	TATCGTTGAGGTTCGACGGG	TCCAACCTTCGCATACTCACC	6FAM-CCCGCGCCGATAACCAATACCA-BHQ-1
HB-067	NR3C1	None	142763209	142763279	GGGTGGAAGGAGACGTCGTAG	AAACTTCCGAACGCGCG	6FAM-GTCCCGATCCCAACTACTTCGACCG-BHQ-1
HB-070	LTB4R	None	23850461	23850536	GCGTTGGTTTTATCGGAAGG	AAACCGTAATTCCCGCTCG	6FAM-GACTCCGCCCAACTTCGCCAAAA-BHQ-1
HB-073	SYK	None	90643560	90643636	GGGCGCGATATTGGGAG	GCGACTCTTCCTCATTTTAAACAAC	6FAM-CCTTAACGCGCCCGAACAAACG-BHQ-1
HB-074	TERT	None	1348267	1348382	GGATTCGCGGGTATAGACGTT	CGAAATCCGCGCGAAA	6FAM-CCCAATCCCTCCGCCACGTAAAA-BHQ-1
HB-075	CDH13	None	81218210	81218312	AATTTCGTTCGTTTTGTGCGT	CTACCCGTACCGAACGATCC	6FAM-AACGCAAAACGCGCCCGACA-BHQ-1
HB-077	NCL	None	232154778	232154864	CGTGTCGTTTCGGTTCGTT	ACCAAAACTCGCGACCGTC	6FAM-CCATAAACCAATCGCGAACCTCTAACCGT-BHQ-1
HB-078	CYP1B1	None	38214997	38215082	GTGCGTTTGGACGGGAGTT	AACGCGACCTAACAAAACGAA	6FAM-CGCCGCACACCAAACCGCTT-BHQ-1
HB-079	SLC6A20	None	45812864	45812998	AGGCGAATACGAATTGTAGCG	TAAAACGACGCGCCTAACG	6FAM-CCGCGCACTAAAACTACCGTACCGAA-BHQ-1
HB-080	TNFRSF25	None	6460427	6460495	GCGGAATTACGACGGGTAGA	ACTCCATAACCCTCCGACGA	6FAM-CGCCCAAAAACTTCCCGACTCCGTA-BHQ-1
HB-081	CDKN2A	None	21964732	21964801	TGGAGTTTTCGGTTGATTGGTT	AACAACGCCCGCACCTCCT	6FAM-ACCCGACCCCGAACCGCG-BHQ-1
HB-082	UNG	None	107998395	107998490	GTTTGACGGAGGGCGTGTA	ACAACGACGACTATTTTAAACACGTAA	6FAM-CCCGAATTTACCGAATCAAAAACGCGA-BHQ-1
HB-083	MBD4	None	130641365	130641480	TCGTGTTTATCGAGTAGGGTTCG	TCGATTACAACCCGATACCGTAA	6FAM-CACACCCTAAACGTTACGACGCTAAACTCG-BHQ-1
HB-084	MSH6	None	47921669	47921753	GGAGTGTTTCGGTTCGGTTAGT	CTACCGCCGACGCCTAAA	6FAM-CCCTTCCCTCACGCCGCGA-BHQ-1
HB-087	OGG1	None	9766425	9766556	TAGGGTGGGCGGGTCG	CCGCGAAACGCCCAA	6FAM-CAATACCGACCAACCGCGCGA-BHQ-1
HB-088	MUTYH/TOE1	None	45474733	45474807	TCGGGTGGATTCGAGTTACG	AAAATTACCTCCCGCGAACTCTA	6FAM-CGCGCCCGACTTTCCGACG-BHQ-1
HB-089	NTHL1	None	2037891	2038004	CGGGACGTCGTCGGAAG	CCGACCTTTCCGCCAAA	6FAM-CGACCCTCCGCGCAATACCG-BHQ-1
HB-090	APEX1	None	19993146	19993280	CGTATTTGTATCGGTTCGATGGTA	GCGCATTCTTCGACCACG	6FAM-CAAACGCGCCTCTAATCACGTAACCAAAT-BHQ-1
HB-091	LIG3	None	30331610	30331701	GTCGCGGGTAGTTTACGACG	CGACCTTAACTCTTACGCCTACG	6FAM-CGCTACCTCCCGCTCTAAAACCCGA-BHQ-1
HB-092	XRCC1	None	48771564	48771673	CGTTGTTAAGGAACGTAGCGTTTT	GCGCGAAACTCGAACCTTT	6FAM-CCAATCGCGCCTCTCCAAAACG-BHQ-1
HB-093	PARP1	None	222902100	222902168	CGGGTTTAGGGAGCGAGC	AAACGACCGCGAACCCATA	6FAM-CGCTCCGAAAACCCGAACCGAA-BHQ-1
HB-094	PARP2	None	19881678	19881744	GGGCGAGAGGTTCGGAGT	TCGTTCCTTTCTAACTACCCGC	6FAM-CCCGCATACCGTCCCGCGATA-BHQ-1
HB-095	MSH2	None	48542284	47542370	TTTTAGTGCGGAGGTACGGG	AAACGATCCTCCGAAACCAAA	6FAM-CCGCACAAACACCAACGTTCCG-BHQ-1
HB-096	MSH4	None	75974790	75974880	CGGATTTTAGGAGATTTTATAGAGTCG	CCGATCGCCCGCAAC	6FAMAACGTACCAAAACAAATAAATACAAAAACCACCTAAACCG-BHQ-1
HB-097	MSH5	None	31815771	31815853	TTCGTGGCGGTCGGTTA	CCGCCATCGCAACGTT	6FAM-CCCGCCTTTTCAATAACCTAAATCGCTACA-BHQ-1
HB-098	PILRB	None	99578411	99578495	TCGTGGTTTGGCGTGGAT	CCTAATACATCGAAATAACGCGTACC	6FAM-CCAACGATCGAAAACCGCCAAACA-BHQ-1

HB-099	MLH3	None	74587699	74587769	TGATGATGGTTGCGCGTAGT	CGACCGCCAAACCGC	6FAM-CGAAACCCTCGCGCATCCGA-BHQ-1
HB-100	XPC	None	14195020	14195117	GTCGGGTGCGTTATTCGC	CTACGCAATTCGCGTCCC	6FAM-ACCGCGCGTTTCCGAACCATATTACT-BHQ-1
HB-101	RAD23A	None	12917467	12917552	TATCGATAACGGGTATGGCGTT	GCAAACTAAACTCCGCGCTATAA	6FAM-TTACTCGACCCGCACACGTAATCTCCTAAA-BHQ-1
HB-102	XPA	None	97539016	97539079	CGCGGAGTTGTTTGTTTCG	CAACATCAATACCCGCTACCG	6FAM-CCGCTCGATACTCGCCCGCA-BHQ-1
HB-104	RPA3	None	7453370	7453448	AGCGCGATTGCGATTTAGG	TTTCTCGACACCAATCAACGAA	6FAM-TCCAACTTCGCCAATTAAATACGCGAAA-BHQ-1
HB-105	ERCC2	None	50565643	50565727	CGAGTTTTCGAGGATGTTTACGA	CCGACCGAACTATACAACGAAAT	6FAM-ACCCGCCTCCCTCATAAATATTCAACGAA-BHQ-1
HB-109	ERCC5	None	102296112	102296188	TAAGCGTAGAAAATATACGTTATGTGCG	CCCGCTCGATTTCCGTCT	6FAM-CGACGCGCAAAACGAAAACTCCG-BHQ-1
HB-111	ERCC4	None	13921544	13921615	TCGACGGATTGTTATGGCG	CCGTCAATATCGAACAATTCCA	6FAM-CACCAACTATCGCTCGTACTCCAACAACG-BHQ-1
HB-114	ERCC6	None	50417137	50417262	ACGTAAGTAGAAAGGCGTTGTTGAG	CGACTCCGACTTCTACTAATACGAAA	6FAM-CCCGTAACGCATACGCCTAACTCAACG-BHQ-1
HB-115	XAB2	None	7600520	7600597	GACGGATAGGTTTACGTTATTGATTTT	CGCATCTTCTAACGCCTCTATTC	6FAM-ACTTCCGATCGCTAACGTCGTCGAAA-BHQ-1
HB-116	DDB1	None	60857034	60857134	GGGCGGAGGTAGCGGT	CCCGTCGAAACTCGAACG	6FAM-CCAACAACGCGCAACGAACTCCA-BHQ-1
HB-117	MMS19L	None	99248168	99248271	TTAGGTAGAAGTCGGTAGGTACGTGA	ATAACTCGAAACGAACTCTCCGC	6FAM-CGCCTCCCGAACCAATCTCCG-BHQ-1
HB-126	BRCA2	None	31787586	31787652	CGTTACGGCGTTACGTGGT	CCGCCTCTACCGCCTAATTT	6FAM-CGCGCCACAAACCCGCG-BHQ-1
HB-133	DCLRE1C	None	15036151	15036236	CGAAGCGCGGGTGATTTA	AAAATCCGAAAACCGAAAACAA	6FAM-ATCCGATCGAATTCTAAACGCCCGCTACT-BHQ-1
HB-139	POLD1	None	55579103	55579174	GGGACGCGGAGGATGC	GATCTAAACGCCGCGATTCTAT	6FAM-TCCTCCCACCCTCGAATATTACGCG-BHQ-1
HB-140	BCL2	None	59136618	59136701	TCGTATTTCGGGATTCGGTC	AACTAAACGCAAACCCCGC	6FAM-ACGACGCCGAAAACAACCGAAATCTACA-BHQ-1
HB-141	TSHR	None	80491125	80491211	TTGAGGGTTAGAGGCGGGTA	ACAACGAAAATCCTCCTCCAAAAATACA	6FAM-AACGACGACTTCGACCGCACCG-BHQ-1
HB-143	MBD4	None	130641481	130641560	GCGTGGAAATGGCGGTT	AATCTAAATATTACGAAAACGCCCC	6FAM-AACGCCGCCGCTACTTTCCGA-BHQ-1
HB-146	CCND1	None	69164885	69164967	GGTAATTTCGTCGTAGGGTAGGC	GAACGCCAAACGCCGA	6FAM-ACCCAAAAACCATCCCTAAAACGCCG-BHQ-1
HB-147	CTSD	None	1741982	1742072	TACGTTTCGCGTAGGTTTGGA	TCGTAAAACGACCCACCCTAA	6FAM-CCTATCCCGACCGCCGCGA-BHQ-1
HB-148	CTSD	None	1741878	1741942	AAGTTGGGTCGGGTTGATTTC	ATAATACGCCTACCGCGCC	6FAM-ACAATTCCCGCCGCTCGCG-BHQ-1
HB-149	PGR	None	100503526	100503701	GGCGGTGACGGTCGTATTC	ACAAACCGTCCCGCGAA	6FAM-AACAACCGCTCGCGCCCGA-BHQ-1
HB-150	MLH1	None	37009766	37009849	AGGAAGAGCGGATAGCGATTT	TCTTCGTCCCTCCCTAAAACG	6FAM-CCCGCTACCTAAAAAAATATACGCTTACGCG-BHQ-1
HB-151	FBXW7	None	153814403	153814526	TGTCGTTGCGGTTGGGAT	CGAAAATAAATAACTACTCCGCGATAA	6FAM-ACGCCAAAACTTCTACCTCGTCCCGTAA-BHQ-1
HB-152	EPM2AIP1	None	37009363	37009450	CGTTATATATCGTTCGTAGTATTCGTGTTT	CTATCGCCGCCTCATCGT	6FAM-CGCGACGTCAAACGCCACTACG-BHQ-1
HB-153	APC	None	112101379	112101452	TTATATGTCGGTTACGTGCGTTTATAT	GAACCAAAACGCTCCCCAT	6FAM-CCCGTCGAAAACCCGCCGATTA-BHQ-1
HB-157	PTEN	None	89612994	89613081	GTTTCGCGTTGTTGTAAAAGTCG	CAATATAACTACCTAAAACTTACTCGAACCG	6FAM-TTCCCAACCGCCAACCTACAACTACACTTA-BHQ-1
HB-158	CACNA1G	None	45993464	45993530	TTTTTTCGTTTCGCGTTTAGGT	CTCGAAACGACTTCGCCG	6FAM-AAATAACGCCGAATCCGACAACCGA-BHQ-1
HB-160	MGMT	None	131155503	131155585	GCGTTTCGACGTTCGTAGGT	CACTCTTCCGAAAACGAAACG	6FAM-CGCAAACGATACGCACCGCGA-BHQ-1
HB-163	PENK	None	57521694	57521792	GGTTAATTATAAAGTGGTTTTAGTAGTCGTTAAG	CAACGTCTCTACGAAATCACGAAC	6FAM-AACGCCTACCTCGCCGTCCCG-BHQ-1
HB-164	ESR1	None	152220942	152221042	GGCGTTCGTTTTGGGATTG	GCCGACACGCGAACTCTAA	6FAM-CGATAAAACCGAACGACCCGACGA-BHQ-1
HB-165	ESR2	None	63830670	63830741	TTTGAAATTTGTAGGGCGAAGAGTAG	ACCCGTCGCAACTCGAATAA	6FAM-CCGACCCAACGCTCGCCG-BHQ-1
HB-166	CALCA	None	14950501	14950601	GTTTTGGAAGTATGAGGGTGACG	TTCCCGCCGCTATAAATCG	6FAM-ATTCCGCCAATACACAACAACCAATAAACG-BHQ-1
HB-167	TIMP3	None	N/A	N/A	GCGTCGGAGGTTAAGGTTGTT	CTCTCCAAAATTACCGTACGCG	6FAM-AACTCGCTCGCCCGCCGAA-BHQ-1
HB-168	HIC1	None	1906660	1906760	GTTAGGCGGTTAGGGCGTC	CCGAACGCCTCCATCGTAT	6FAM-CAACATCGTCTACCCAACACACTCTCCTACG-BHQ-1
HB-169	PGR	None	100505562	100505655	TTATAATTCGAGGCGGTTAGTGTTT	TCGAACTTCTACTAACTCCGTACTACGA	6FAM-ATCATCTCCGAAAATCTCAAATCCCAATAATACG-BHQ-1
HB-170	CTNNB1	None	41215587	41215667	GGAAAGGCGCGTCGAGT	TCCCCTATCCCAAACCCG	6FAM-CGCGCGTTTCCCGAACCG-BHQ-1
HB-171	CDH1	None	67328515	67328584	AATTTTAGGTTAGAGGGTTATCGCGT	TCCCCAAAACGAAACTAACGAC	6FAM-CGCCCACCCGACCTCGCAT-BHQ-1
HB-172	GSTP1	None	67107783	67107882	GTCGGCGTCGTGATTTAGTATTG	AAACTACGACGACGAAACTCCAA	6FAM-AAACCTCGCGACCTCCGAACCTTATAAAA-BHQ-1
HB-173	CDKN2B	None	21998980	21999060	AGGAAGGAGAGAGTGCGTCG	CGAATAATCCACCGTTAACCG	6FAM-TTAACGACACTCTTCCCTTCTTTCCCACG-BHQ-1
HB-175	KL	None	32488560	32488687	AGTTTGGTTTTCGCGTAGTATGTTC	CGCCCGACTCCGCAC	6FAM-CGAACGACGCGACGAAACGCT-BHQ-1
HB-177	TP73	None	3592223	3592304	GGGTCGGGTAGTTCGTTTTG	CGATTTCGCTACGTCCCCT	6FAM-AACCTCCGAACGAATACGCGAACGAA-BHQ-1
HB-178	DCC	None	48121053	48121210	GGGTTCGGCGCGTGT	CGAAAAATACAAAAACCAACTTAAATACC	6FAM-ACCAAAAATCGCGAACAACGACAACACT-BHQ-1
HB-179	ATM	None	107599021	107599090	ACGGAGAAAAGAAGTCGTGGTC	GCGACGATAACTACAACGCAAAT	6FAM-CGACTCCTCTCGCCTCCTCCCG-BHQ-1
HB-180	ATR	None	143780282	143780372	AGCGGTTTTCGGGAGGAGT	GAATTCCCGACGTCTCCAAA	6FAM-CGACGCCCGACGAAACCGTATAA-BHQ-1
HB-181	RUNX3	None	25001393	25001509	CGTTCGATGGTGGACGTGT	GACGAACAACGTCTTATTACAACGC	6FAM-CGCACGAACTCGCCTACGTAATCCG-BHQ-1
HB-182	STK11	None	1157626	1157718	TGAGGTTCGGGTTTTATTGGAA	GAATCCAAACCCGATACAAAATCT	6FAM-CGAAAATCCGAAACGACGACTCAAACG-BHQ-1
HB-183	STK11	None	1156690	1156793	AATTAACGGGTGGGTACGTCG	GCCATCTTATTTACCTCCCTCCC	6FAM-CGCACGCCCGACCGCAA-BHQ-1
HB-185	RBP1	None	140741145	140741234	CGCGTTGGGAATTTAGTTGTC	GATACTACGCGAATAATAAACGACCC	6FAM-ACGCCCTCCGAAAACAAAAAACTCTACG-BHQ-1
HB-186	ARPC1B	None	98616846	98616917	TGCGCGGGTATCGGTAGTAT	ACCTAAAACAACGATCGCGAAAT	6FAM-CAAATCCCGCCCTCCCTTCGAAAT-BHQ-1
HB-187	MINT2	None	58567574	58567662	TTGAGTGGCGCGTTTCGT	TCCCCGCCTAAACCAACC	6FAM-CTTACGCCACCGCCTCCGA-BHQ-1
HB-190	CHFR	None	132074209	132074312	CGGGAGTTTTTATGGGCGT	AACCGTCCCCAAAACTACGAC	6FAM-CCTCGAACCGCTCCATCGAAATTCA-BHQ-1

HB-191	VHL	None	10158449	10158542	CGGGAGCGCGTACGTAGTT	CTCCGAAACATTCCCTCCG	6FAM-CGAACCGAACGCCGCGAAA-BHQ-1
HB-192	TGFBR1	None	98946812	98946910	ACGCGCGTTTATTGGTTGTC	ACGAACCCGCAAACGAAA	6FAM-TAAATCCCGCTTAACAACTCGCGACGA-BHQ-1
HB-194	SCGB3A1	None	179950956	179951042	GGCGTAGCGGGCGTC	CTACGTAACCCTATCCTACAACTCCG	6FAM-CGAACTCCTAACGCGCACGATAAAACCTAA-BHQ
HB-195	CDX1	None	149526555	149526622	TGAGCGGTTGTTCGTCGTC	AAATCCCCCGCGCATACTA	6FAM-CCTAAAACCGCCGCTACCGACCG-BHQ-1
HB-196	ARF/CDKN2A	None	21984540	21984608	ACGGGCGTTTTCGGTAGTT	CCGAACCTCCAAAATCTCGA	6FAM-CGACTCTAAACCCTACGCACGCGAAA-BHQ-1
HB-197	CRABP1	None	76419794	76419875	TCGAAATTTTCGTTGTTGCGT	TATCCGTACCTACCGCCGC	6FAM-ACCATACCCAACTTCGCCGACACCTAA-BHQ
HB-198	FAM127A***	None	133891933	133892014	GATGGACGGTCGGGTGTAGTT	AATCGAATTCCTCCAACGACG	6FAM-TCGCAAACCGAATCGACAAAACCAA-BHQ
HB-203	JUP	None	37196423	37196513	GGATAGCGAATTGAGTTCGGC	CTCTTCGCCTTTTATTCGATTACTAAAT	6FAM-AACAACCGCCGCCCGACCA-BHQ-1
HB-204	MT1G	None	55259560	55259636	CGTTTAAGGGATTTTGTATTTGGTTTAT	CCGCTAAATCCGCACCG	6FAM-CGCGATCCCGACCTAAACTATACGCA-BHQ-1
HB-209	OPCML/HNT	None	132319029	132319100	CGTTTCGAGGCGGTATCG	CGAACCGCCGAAATTATCAT	6FAM-AACAACTCCATCCCTAACCGCCACTTTCT-BHQ-1
HB-211	PAX8	None	113752214	113752309	GTTCGTAGTTCGTCGAGGGTTC	CGCATCTCATACCCTTCTCCTAAAT	6FAM-CAAACGCGACCCGAACCTACGAAAA-BHQ-1
HB-213	TITF1	None	36058456	36058584	CGAAATAAACCGAATCCTCCTTAA	TGTTTTGTTGTTTTAGCGTTTACGT	6FAM-CTCGCGTTTATTTTAACCCGACGCCA-BHQ-1
HB-214	PRKAR1A	None	64019490	64019573	CGGATTTGTAGTAGTTGCGTTGC	ACCGAACACAAAATACGCGAC	6FAM-CATCCCGACCATCCGCCCG-BHQ-1
HB-216	THRB	None	24511656	24511731	TCGTCGTCGTTATCGTCGC	GCGTCTACGAACCGATAACCTAAT	6FAM-CCCTCCAACCCTCACGACTATCCGACTTA-BHQ-1
HB-217	WDR79	None	7532404	7532486	TTTGTTGTCGCGGGATTTC	CGAATTCCGTAAATCGCCC	6FAM-TAATCCGAAATACGACGACCCAATCGAAAA-3'BHQ
HB-218	DLC1	None	13034914	13034989	AGTAAGGATGCGTTGAGGATCG	ACGACTCGACTTCCGCGTC	6FAM-AACCCACGACGACACCCGAAACG-BHQ-1
HB-221	GDNF	None	37875633	37875741	CGGTAGTTGTCGTTGAGTCGTTC	AACAACCGCCGCTACTTTAAATA	6FAM-CGCGCGTCGCGCTCTTAACTAAAA-BHQ-1
HB-222	GDNF	None	37870607	37870697	TCGTTTGTTCGCGTAGGTGTC	CGATATAAAACAACACCAAACAAACAAC	6FAM-TCCCATAACTTCATCTTAAAATCCCGTCCG-BHQ-1
HB-224	LRRC41	None	46480839	46480910	TTCGGTTTCGGGTTTTAACG	CCCATATAAACGCTCACCGC	6FAM-CCCGCACAACTCGAACAAAACGAAA-BHQ-1
HB-225	DLEC1	None	38055976	38056105	TCGTTGCGTATTTAAGATATTTCGTATT	CGTAACGCTCATTCTCGCTACC	6FAM-TAATCAAACTTACGCTCACTTCGTCGCCG-BHQ-1
HB-226	CDK2AP1	None	122281168	122281288	CGCGGAAAGTTTGCGGT	CGCACTTTTTATTATCGACGACTC	6FAM-CGACAAATATAACCGTCCGCGCCCTA-BHQ-1
HB-227	AXIN1	None	342144	342213	CGGTTTTTGTAGTTGTTTCGTGTT	CGACGCGATAACCGCTTAAA	6FAM-ATCCGAAACCTCGAACGCGTCTCG-BHQ-1
HB-228	PYCARD	None	31121237	31121332	TTGGAGATTTACGGCGTCG	ACCCTAATACGTAACCGCCTACAA	6FAM-CATCTCCTACAAACCCATATCGCGCAA-BHQ-1
HB-229	EBF3	None	131652354	131652431	GTAGGATATTGCGGGATCGTTC	GCAACACTCACTACCCCGTTTAT	6FAM-TCTTTAAAACAAACGAACCGCGCCAA-BHQ-1
HB-230	CDKN1A	None	36754719	36754810	CGCGTTCGGTTTGCGTAT	TTATAATCCCGCTCTCCGCC	6FAM-AAATCTCCGACACATCCCGACTCTCGT-BHQ-1
HB-231	PSAT1	None	78141710	78141790	TGGGTTTGGTTTCGTTAAGTTGT	ACGTACTCCCGCCTAAACCTC	6FAM-ACGCCCGCTCGCGAAAACTTACTAAATA-BHQ-1
HB-232	ERBB2	None	35110117	35110193	AGTAGCGGCGGGATTTTGT	ACTCGTCCGAACCCCTCTAAC	6FAM-CGCCGACCGCGAAACTCG-BHQ-1
HB-236	PITX2	None	111901970	111902049	GTGGTTTCGTATTAATTAGGACGTTTC	CCGACCCCGCACTACTAATAAAT	6FAM-ACAAAACAATCCCTCCACCGCGACT-BHQ-1
HB-238	CYP1B1	None	38213993	38214078	GGGTTAGCGGTTGTTGAGGTAAC	CCGCGTTTCCGATCAATAA	6FAM-AAAACGCGAACCGAAACTACCGCCT-BHQ-1
HB-243	ONECUT2****	None	53256301	53256419	CGTTACGTATATCGCGCGG	CAAAAACCTCCTXXXATAAACGACGAAT	6FAM-AACACGCAATTACGCGCTTTTATACGCA-BHQ-1
HB-245	RB1	None	47775771	47775890	TTAGTTCGCGTATCGATTAGCG	ACTAAACGCCGCGTCCAA	6FAM-TCACGTCCGCGAAACTCCCGA-BHQ-1
HB-246	TGFBR2	None	30623298	30623377	GCGCGGAGCGTAGTTAGG	CAAACCCCGCTACTCGTCAT	6FAM-CACGAACGACGCCTTCCCGAA-BHQ-1
HB-249	AR	None	66546885	66546963	GCGTTTTTTTCGAGATTTCGG	GCCTCCTCTACCTATAAACTTACTCCG	6FAM-ACCGTCCCGCTCTCCCAACAAACTA-BHQ-1
HB-250	GRIN2B	None	14025182	14025264	GTCGGATTTACGCGTCGAGT	CTACCGCCGCGCTAAAATAC	6FAM-ACGCACGAAACTTCACCTACAACGTATCG-BHQ-1
HB-252	DRD1	None	174804232	174804303	GGCGCGCGTTGGTTC	TACCCGTAAACGCCTATACTCACC	6FAM-CTCGCAAAAAAAACGCGACGCAACTA-BHQ-1
HB-255	GAD1	None	171497859	171497935	ATTTTTATTAGAGGGCGTTAAGAGTTTAGA	CGCTCGAACGCTAACGAAA	6FAM-CCCGAAAACGCAAAACCTCTCCGT-BHQ-1
HB-260	NEUROD2	None	35017643	35017731	GGTTTGGTATAGAGGTTGGTATTTCGT	ACGAACGCCGACGTCTTC	6FAM-CGCCATACGAACCGCGAAACGAATATAA-BHQ-1
HB-261	NEUROG1	None	134899670	134899757	CGTGTAGCGTTCGGGTATTTGTA	CGATAATTACGAACACACTCCGAAT	6FAM-CGATAACGACCTCCCGCGAACATAAA-BHQ-1
HB-264	PSEN2	None	223365485	223365573	GAGGCGTGTAGTAGGCGGG	CCGATACTAAAAACCGAATAAACTCG	6FAM-CGCAACGAAAATCTCCGACGAAAAAA-BHQ-1
HB-265	PSEN2	None	223365233	223365337	TTTTGCGTGCGTAGCGTTT	CTTCCACGACCCAATTCCG	6FAM-CGCCGCCTACCGAACACTCTCCTCT-BHQ-1
HB-266	APP	None	26465290	26465385	AACGAAATGCGGATAAAAACGTAT	TCGTCCCCGTAAACTTAAATCATC	6FAM-CCCGCAAACCTCCCGAAAATATCGTATAAA-BHQ-1
HB-267	HOXA1	None	26909068	26909138	GTTGTTGCGGCGATTGTAAA	CGCGCAAAACGCAACTT	6FAM-TACTCTTCTTCGCTCCAACACTCCAAATCG-BHQ-1
HB-271	HOXA10	None	26992754	26992829	ATCGGAAGTGCGTTATTTCGTG	TTCCGTCTCTCGACTCGAAACT	6FAM-CTCCGACCCAACCTAAACGCGCA-BHQ-1
HB-272	HOXA11	None	26998639	26998738	TTTTGTTTTCGATTTTAGTCGGAAT	TAATCAAATCACCGTACAAATCGAAC	6FAM-ACCACCAAACAAACACATCCACGACTTCA-BHQ-1
HB-274	TMEFF2	None	192885270	192885342	CGACGAGGAGGTGTAAGGATG	CAACGCCTAACGAACGAACC	6FAM-TATAACTTCCGCGACCGCCTCCTCCT-BHQ-1
HB-275	SMAD2	None	43711755	43711832	CGAGGCGGTAGGTTTTTATAGGT	CGCATTAAAACGATTCCCGAT	6FAM-CCGATCCCTCGCCAACGTCGTAA-BHQ-1
HB-276	SMAD2	None	43710779	43710916	GTCGTCGGTCGGGTTCG	CCTTTTATTCCTTCCTCTTCCGAT	6FAM-AAAAACACTCCCGACCGCCGTCTT-BHQ-1
HB-277	SMAD4	None	46810459	46810528	GTTTGCGTAGAGCGATTTTTTTC	GCAACTTTCCTTTCTCCCGACT	6FAM-CCCGCCTCCCGCTCCGAATA-BHQ-1
HB-280	SFRP2	None	155067644	155067735	GCGTTTTAGTCGTCGGTTGTTAGT	AAACGACCGAAATTCGAACTTATC	6FAM-CGAACCCGCTCTCTTCGCTAAATACGA-BHQ-1
HB-281	SFRP4	None	37729547	37729625	GTTGTTCGGGCGGGTTC	GCGAAACTCCGCCGTCTA	6FAM-AAACACGAACAACGCCAACTCTCAACCT-BHQ-1
HB-282	SFRP5	None	99521371	99521463	GCGTTTGTAGTTATCGTGTGGTAGA	GAACCGCTACACGACCGCT	6FAM-CGCCGCAATACCTTAACATCCCTACCG-BHQ-1
HB-303	CLDN7	None	7105597	7105689	CGGTTTAAGTATCGTTGTTTCGAG	GACTACACGTAAACGCGCACC	6FAM-CGATCGCCCACGTTCCTCTTCCT-BHQ-1

HB-304	FAF1	None	51138014	51138088	CGTTTTGCGGTTTTACGTGA	CAACGCAAAAATCCTAACCGAA	6FAM-CGCGCGCTCAACGCTTAACAAAAAAATA-BHQ-1
HB-305	FAF1	None	51137773	51137850	GGATGTGGGTTCGGTTTTATAGTC	CCGCTAAAAATATCCGAATACGC	6FAM-ATCGCAACCTACCCTCATCCTAACCCG-BHQ-1
HB-307	TNFRSF10B	None	22982682	22982764	TTTTGGCGGTTGCGTTTC	CTCATTTCCCCCAAATTTCGAT	6FAM-ATCCTAACGCGAACAAAACCCAAAAACAA-BHQ-1
HB-308	TNFRSF10C	None	23016667	23016789	GGGAAGAGCGTATTTGGCG	TCCCCTAACTCCGACGACG	6FAM-CGAACATACCCGACCGCAAATAACCA-BHQ-1
HB-309	TNFRSF10D	None	230770092	23077216	GGGAAGAGCGTATTTGGCG	TCCCCTAACTCCGACGACG	6FAM-TACCCGACCGCAAACGACCCG-BHQ-1
HB-314	TFAP2A	None	10523187	10523295	CGTTAATTTTTAAAGTATTTTTATGGATCG	CCGACAACCAACACTTTACGC	6FAM-CGAAACCGAAAAAAACATATCCGTTCACG-BHQ-1
HB-315	SMAD9	None	36392381	36392455	CGCGAAGTTTTATCGTTCGTATTAG	CGAAAACGAACCGCAAACA	6FAM-AACTCCCTAACCGCTTTCCAAATCGACG-BHQ-1
HB-316	EYA4	None	133604237	133604343	GGAAAGAGTTGCGGGAAAAGT	ACCAAAACTCCGAACTACGACAAA	6FAM-AACGCGCCCAACCGCCG-BHQ-1
HB-317	EYA4	None	133604142	133604208	TGGATAGGATGGAAGTTTTGCG	AACTACCGACAACGCGACG	6FAM-CGCTCCGACCGTTCCCGACTT-BHQ-1
HB-318	IGF2	None	2117157	2117238	GGTTGGCGCGATTATAAGAGTC	AACCGACCGAACCTCACG	6FAM-AACTCCGCCGAAAAACAAACGAACTACG-BHQ-1
HB-319	IGF2	None	2116714	2116801	GAGCGGTTTCGGTGTCGTTA	CCAACTCGATTTAAACCGACG	6FAM-CCCTCTACCGTCGCGAACCCGA-BHQ-1
HB-320	NKD2	None	1062127	1062205	CGGTTTATCGTGTTTCGATTGTAGT	CAACCCTACACCTAAACGCGA	6FAM-CCCGACGAAACGTAACGACGACGATAA-BHQ-1
HB-321	ITGA4	None	182147511	182147581	TGCGGAGGCGTAGGGTC	CAACCGAAATTCCCCAACG	6FAM-CCTACAACCGCGCGTAAACAAAAACG-BHQ-1
HB-322	RARRES1	None	159932677	159932741	GGCGAGTCGGATCGGAA	CGCAAACTCCTACAACAAACGA	6FAM-CGCGCGACGCTTCACTTCTTCAA-BHQ-1
HB-323	GATA4	None	11599555	11599628	GATGGTGGTCGCGTGAAGTTA	TTCCCTCCATATACGAACTACCG	6FAM-CCTATCCCGAATCCGTCAATCCCG-BHQ-1
HB-324	GATA4	None	11602786	11602873	GGCGAGGAGGAAGGAGTTAGTT	GAATCCCCCACTCGTCACAC	6FAM-CCGCGACCACAAACGCAAAAACTACT-BHQ-1
HB-326	GATA5	None	60484577	60484661	AGTTACGTGATTTTGGTAGGTTTTGTT	TAATCCGAACTCCGCGCTA	6FAM-CCCGTATCGTACGTCCTTATCGCCAAA-BHQ-1
HB-328	CDKN1C	None	2863369	2863454	TTATCGGATAGTTAGGTAGTCGTCGC	AAAACGAAAACCGAACGCAA	6FAM-CGCGCCGCCCGACTCTACGTAT-BHQ-1
HB-329	CDKN1C	None	2862551	2862625	TCGAGTAGGGCGCGAATTAG	GTCCCGAAATCCCCGAAT	6FAM-AACTAATCAACGAAAAACTCCTAACCGCGCT-BHQ-1
HB-334	MUTYH/TOE1	None	45475024	45475101	GTTTTGAAGGTTATTTTTGGGAAGTC	GCCGACAATAACGATAACGCA	6FAM-TCAACTCCCGCAACTTCCGACGATA-BHQ-1
HB-335	TUSC3	None	15442252	15442317	CGCGGTAGGA GTTGGGC	CCCCCATCGCGACAAAT	6FAM-TCTCCTCCACGCGCGATAACCGT-BHQ-1
HB-336	SOCS4/WDHD1	None	54563562	54563642	TATATTGCGTTTGCGTCGATTC	ACGCCGCAACTAACGCC	6FAM-CCCGACGACCTCTATCGAAACCTACGCTA-BHQ-1
HB-337	SOX4	None	21702727	21702814	GTTCGTCGAGTCGAGGTTATGG	CGCCCGAATCCGAACTCT	6FAM-CCAACAACGCTTCCGTATTCTCGACATT-BHQ-1
HB-340	PTEN	None	89613229	89613308	TCGGTTTTTCGAGGCGTTC	CGCGTATCCTACCGCAACG	6FAM-CACATCACCGCCCGCCGACCTA-BHQ-1
HB-341	PTEN	None	89613229	89613308	TTTCGGAGGTCGTCGGC	CTACGACGACTACTAAATAATTACAACGACT	6FAM-AACAACCAAAAACCTAACAACGACGACAACG-BHQ-1
HB-342	PTEN	None	89613229	89614064	TTTCGGAGGTCGTCGGC	GCAACCGAATAATAACTACTACGACG	6FAM-AACAACCAAAAACCTAACAACGACGACAACG-BHQ-1
HB-343	PTEN	None	89613911	89614051	TTTCGGAGGTCGTCGGC	AACTACTACGACGACTACTAAATAATTACAACG	6FAM-AACAACCAAAAACCTAACAACGACGACAACG-BHQ-1
HB-350	PTPN6	None	6926164	6926293	GTAAGTTTCGGGTATTATCGGGGTT	AACCGAAAACAAACGCAAAACA	6FAM-AAATCGCACTCACGTTCACACACGTCGAA-BHQ-1
HB-361	TFPI2	None	93164648	93164723	TTGGAGTAGAAAGTCGCGTATTTTT	AAAAATCGAACGACCCGCTA	6FAM-ACAAAACGTCCGAAAAAACGCCTAACGAA-BHQ-1
HB-362	CXCR4	None	136709382	136709472	TCGGTCGCGGTTAGAAATTT	AATACGATAACTACTAAAACACTCAAACCCT	6FAM-ACGTCACTTTACTACCTACTACCGCAACCAACAAA-BHQ-1
HB-363	BNIP3	None	133645457	133645525	TGCGTACGCGTAGGTTTTAAGTC	CAAAACGAAACCGAACGAATCT	6FAM-CGACCGCGTCGCCCATTAACC-BHQ-1
HB-364	MINT32/ZFP64	None	50155080	50155158	GGTTTTTTCGTTATTTGCGGG	CCCAACACCTCAACTCCGA	6FAM-CGCGACCTACGCGCCGAAAATATA-BHQ-1
HB-386	OAZ1	None	2220665	2220783	AATTTAGCGTTATTATTTACGTTAGTCGTATT	CGACGCTTCTAAAAACTTCGAAC	6FAM-CGCCGCGACTATACAAACTTAAAAACGACA-BHQ-1
HB-387	SAT1	None	23560691	23560805	CGTGTGAATTCGGAGGATAAAAGT	CACCGACTCCCAATAAAATTTCC	6FAM-CGCGAAACGCCACTAAAAAACGCC-BHQ-1
HB-388	ODC1	None	10539638	10539727	TTAGGGAGTAGAGGCGCGG	CGCGAAAACGTAAAAACGACT	6FAM-CCAACCCTACCCGCAACGTACTAACGA-BHQ-1
HB-389	SHARBEY locus	None	4445358	4445473	CGTTTTTTTCGTTTAATTATCGGAG	TAAAACCGAAACCCGCGAT	6FAM-ACGACTAACTCCGAACCGCGACCG-BHQ-1
HB-390	LHFPL4	None	9569077	9569177	TATTATTGCGTGGGTAGCGG	ACGACCGCCTTAAAAACGCT	6FAM-CCGACAAATAAACTCGCGACCCGCC-BHQ-1
HB-392	PTPRN2	None	156981836	156981929	CGTTTTAATAGTTTCGGGTTTAGTTATAAGT	AACTACGCTTTCTCAACGCCTC	6FAM-TAAAACGACCGCGTACTCGCCAAAAAA-BHQ-1
HB-394	2C35	None	23502454	23502526	TCGTTATTTAGGCGGTCGTTGT	ATCAACCCCATTCTTACGCTTC	6FAM-CAAAACCCGCGACGCAACGAAA-BHQ-1
HB-396	PCTK2	None	95296605	95296679	TAGTCGTCGAGTGTGAAAGCGA	CCGCGAAAAACTCAAAAAACTAC	6FAM-ACGACCGCGTTCGCTCCTTACG-BHQ-1
HB-397	RNF135	None	26322491	26322594	ATATAGGCGGGTTTCGATTTTGTT	CCTACCCGCTACAATTCCGA	6FAM-CGAAACCTCGCGACCGCGCTA-BHQ-1
HB-398	GP1BB	None	18085438	18085506	TGGGAGCGGAAGTTTGAGC	AACGCGCGCTACAACGAC	6FAM-AATAACACACTATCGCCGAAAACCGCAAA-BHQ-1
HB-401	GATM	None	43458059	43458132	TTTTTGTAGTCGCGTTTCGTTTC	GCCGACCCCACCACCTAT	6FAM-CGACAACCAATAAAACCGCGAAAAACGA-BHQ-1
HB-402	ECH1	None	44014197	44014286	TTACGTCGGGTTAGTAGGTCGC	CTCCGTCCGAACGCAATAAA	6FAM-CGACGATAACGACGAAAATAATAACTTCTCGCA-BHQ-1
HB-405	COMP	None	18762980	18763072	CGGGTGTTAACGCGGTTT	CCCGACACCGCCTACGT	6FAM-CTTCTACTCACCCTAACTACCCTCGACGCG-BHQ-1
HB-406	COMP	None	18760492	18760565	CGTCGTTGGTAGTCGGACGT	ACGCAAAACTCCTTCCAATACG	6FAM-CCCGTACCAACCCGACTTCGTAAACG-BHQ-1
HB-407	EIF3S6IP	None	36569780	36569878	TGAGTTACGGAACGCGGTTT	CCCACTCGAACCTACCGAAA	6FAM-CGAAAACCTTTCCGAAACCTCTAACCATCTAAA-BHQ-1
HB-408	FKBP2	None	63765045	63765119	GTTAGCGGGCGTGTGGTC	TCGTCGCTACCAAACCGAAC	6FAM-CGCCGCCCTCCCTTATTAAACCGT-BHQ-1
HB-409	OPCML	None	132318804	132318904	GTTCGTTTAGTTTTTCGTGCGTTC	TCCGATCGATAACCGTCCTCTA	6FAM-CGATCGACGACTCGCGACCAACC-BHQ-1

	HB-410	HNT	None	131285898	131285982	TTCGTCGAGTTTCGTCGGATA	GAAATCCTACGACAACCGCCT	6FAM-CAACTTCCTCGAAACCCAAATACGCGCT-BHQ-1
**C**	HB-043	DIRAS3		68228349	68228434	GCGTAAGCGGAATTTATGTTTGT	CCGCGATTTTATATTCCGACTT	6FAM-CGCACAAAAACGAAATACGAAAACGCAAA-BHQ-1
	HB-493	MEST		6058	6144	CGGCGTTCGGTGTTTTGTAA	CACACTCACCTACGAAAACGATCTC	6FAM-ACGCACCATAACCGCGTTATCCCATACC-BHQ-1

* Unidentified Interspersed Repeat (UIR) within Caspase 8 (apoptosis-related cysteine protease) sequence.
** This is the seq derived from clone AF135501. The bigger genomic clone we used for CpG island location has mismatched with the FW pr
*** The FAM127A gene with its promoter is highly similar(90%) with 2 other uncharactyerized adjacent genes and their promoters, DKFZ56B417 (to the right) and BC048268 (to the left), both on the opposite strand. The promoters are CpG islands for all these 3 genes. There are few mismatches in the primer/probe seq of the FAM127A amplicon relative to the other genes so that is very likely that all these 3 genes are targeted for amplification.
**** Antisense primer is missing 3 nucleotides (XXX). These should be GTG.

$^{\phi}$ C(t) of 'None' indicates that no methylation was detected by this reaction.
