# Supplementary material for: Widespread Epigenetic Abnormalities Suggest a Broad DNA Methylation Erasure Defect in Abnormal Human Sperm
Source: PLoS One. 2007 Dec 12;2(12):e1289. doi: 10.1371/journal.pone.0001289 (PMC2100168; doi:10.1371/journal.pone.0001289)
Supplement: Table S2 — Gene symbols, probe IDs, and measured β-values of Illumina analysis of 1,421 autosomal sequences. This panel is a subset of the GoldenGate Methylation Cancer Panel I described at www.illumina.com.Tertiles and heat map of methylation level of CpG loci are as shown in Figure 3. β-values of all loci for all samples are given. β-values of the heat map are as follows: Green, β<0.1; yellow, 0.1≤β≤0.25; orange, 0.25<β≤0.5; red, β>0.5. A to G represent 7 sperm samples selected from 65 study samples and are ordered from left to right from lowest to highest sperm concentration. S, Pre Screen sample; 1 and 2: Buffy Coat samples 1 and 2, respectively; Human maternal or paternal imprinted loci are indicated with a filled box: IMP, Imprinted; MI, Maternally Imprinted; PI, Paternally Imprinted. (0.20 MB PDF) [file pone.0001289.s002.pdf]

Table S2. **Gene symbols, probe IDs, and measured β-values of Illumina analysis of 1,421 autosomal sequences.** This panel is a subset of the *GoldenGate Methylation Cancer Panel I* described at www.illumina.com. Tertiles and heat map of methylation level of CpG loci are as shown in Figure 3. β-values of all loci for all samples are given. β-values of the heat map are as follows: Green, β <0.1; yellow, 0.1≤ β ≤0.25; orange, 0.25< β ≤0.5; red, β >0.5. A to G represent 7 sperm samples selected from 65 study samples and are ordered from left to right from lowest to highest sperm concentration. S, Pre Screen sample; 1 and 2: Buffy Coat samples 1 and 2, respectively; Human maternal or paternal imprinted loci are indicated with a filled box: IMP, Imprinted; MI, Maternally Imprinted; PI, Paternally Imprinted.

SYMBOL	PROBE ID	SAMPLE									
		A	B	C	D	E	F	G	S	1	2
TYRO3	5237	0.01	0.02	0.01	0.01	0.02	0.01	0.01	0.01	0.02	0.03
IGFBP3	2706	0.01	0.01	0.01	0.01	0.02	0.01	0.01	0.02	0.02	0.07
FGF3	1333	0.02	0.01	0.01	0.01	0.01	0.01	0.01	0.01	0.02	0.02
SHB	2347	0.02	0.01	0.02	0.02	0.01	0.01	0.01	0.01	0.01	0.02
CCNC	5518	0.02	0.01	0.01	0.01	0.01	0.01	0.01	0.01	0.02	0.02
RBP1	3868	0.02	0.02	0.01	0.01	0.02	0.01	0.01	0.01	0.02	0.05
ALPL	5846	0.02	0.01	0.01	0.01	0.02	0.02	0.01	0.01	0.02	0.02
COL6A1	1990	0.01	0.01	0.01	0.02	0.02	0.01	0.02	0.01	0.02	0.03
NRG1	5093	0.02	0.02	0.02	0.01	0.01	0.01	0.02	0.01	0.07	0.09
ERBB4	5633	0.02	0.01	0.02	0.01	0.02	0.02	0.01	0.01	0.03	0.04
FHIT	2728	0.01	0.01	0.03	0.01	0.02	0.02	0.02	0.02	0.02	0.03
SMAD4	5229	0.01	0.01	0.02	0.01	0.02	0.02	0.02	0.02	0.02	0.03
LAMC1	5629	0.02	0.01	0.02	0.01	0.02	0.02	0.02	0.01	0.02	0.04
PTPRO	3842	0.01	0.01	0.02	0.02	0.02	0.02	0.02	0.01	0.03	0.02
MT1A	1564	0.03	0.01	0.01	0.02	0.02	0.02	0.01	0.02	0.02	0.04
RBP1	1838	0.02	0.02	0.02	0.01	0.02	0.02	0.02	0.02	0.02	0.04
PTPNS1	3841	0.01	0.02	0.02	0.02	0.01	0.02	0.02	0.01	0.02	0.02
PTPRG	5055	0.02	0.01	0.02	0.01	0.02	0.02	0.02	0.02	0.03	0.05
PTPNS1	1784	0.03	0.01	0.01	0.02	0.02	0.02	0.02	0.02	0.03	0.03
ABCB1	6068	0.03	0.02	0.02	0.01	0.02	0.02	0.02	0.02	0.02	0.03
RASSF1	1836	0.02	0.01	0.02	0.02	0.02	0.02	0.01	0.02	0.02	0.02
SHB	2342	0.02	0.01	0.02	0.01	0.02	0.02	0.02	0.02	0.02	0.02
ERBB2	2442	0.02	0.02	0.02	0.02	0.02	0.02	0.02	0.02	0.03	0.02
MLH1	2741	0.02	0.03	0.01	0.01	0.02	0.01	0.01	0.02	0.02	0.02
HLA-F	3465	0.02	0.02	0.02	0.02	0.02	0.02	0.02	0.02	0.03	0.02
HSD17B12	3484	0.02	0.02	0.02	0.02	0.02	0.02	0.02	0.02	0.03	0.03
TK1	5348	0.02	0.02	0.02	0.02	0.02	0.02	0.02	0.02	0.03	0.02
RASGRF1	3860	0.02	0.02	0.01	0.02	0.02	0.02	0.02	0.01	0.02	0.03
BMP2	1884	0.03	0.03	0.02	0.02	0.02	0.01	0.02	0.02	0.04	0.04
DSP	3736	0.02	0.02	0.02	0.02	0.02	0.02	0.02	0.02	0.03	0.03
GPX1	5567	0.02	0.01	0.02	0.03	0.02	0.02	0.02	0.02	0.03	0.02
EPHA1	3871	0.02	0.02	0.02	0.02	0.02	0.02	0.02	0.02	0.02	0.02
MEST	1392	0.03	0.02	0.02	0.02	0.02	0.02	0.02	0.02	0.05	0.07
MLLT3	905	0.02	0.02	0.03	0.02	0.02	0.02	0.02	0.02	0.03	0.03
SMARCA4	5180	0.02	0.02	0.02	0.02	0.02	0.03	0.02	0.02	0.02	0.02
NQO1	4893	0.04	0.02	0.01	0.02	0.02	0.02	0.02	0.02	0.03	0.05
PLAUR	4261	0.02	0.02	0.02	0.02	0.02	0.02	0.02	0.02	0.04	0.04
PTEN	2827	0.02	0.02	0.02	0.02	0.02	0.02	0.02	0.03	0.03	0.02
ABCA1	5366	0.02	0.02	0.02	0.02	0.02	0.02	0.02	0.02	0.03	0.03
BMP3	1700	0.02	0.02	0.02	0.02	0.02	0.02	0.02	0.02	0.03	0.03
IGFBP2	5664	0.02	0.02	0.02	0.03	0.02	0.02	0.02	0.02	0.02	0.09
KDR	4232	0.02	0.02	0.02	0.02	0.02	0.02	0.02	0.02	0.04	0.09
ERBB4	5632	0.04	0.02	0.02	0.02	0.02	0.02	0.02	0.02	0.03	0.04
SMAD2	6035	0.02	0.02	0.02	0.02	0.02	0.02	0.02	0.02	0.02	0.05
DCC	2424	0.03	0.02	0.02	0.02	0.02	0.02	0.02	0.02	0.05	0.11
HFE	3397	0.02	0.02	0.02	0.01	0.02	0.02	0.02	0.02	0.04	0.04
EFNA1	2057	0.02	0.02	0.02	0.04	0.02	0.02	0.02	0.02	0.03	0.02
MYBL2	4173	0.02	0.02	0.02	0.02	0.03	0.02	0.02	0.02	0.03	0.03
DAB2	3569	0.02	0.02	0.02	0.02	0.02	0.02	0.02	0.02	0.04	0.06
ICAM1	2658	0.04	0.02	0.02	0.02	0.02	0.03	0.02	0.02	0.03	0.03
WNT5A	5397	0.02	0.02	0.02	0.04	0.02	0.02	0.02	0.02	0.02	0.05
RHOC	5160	0.02	0.02	0.02	0.02	0.03	0.02	0.02	0.02	0.04	0.04
NTRK2	4909	0.02	0.02	0.02	0.03	0.02	0.02	0.02	0.02	0.03	0.05
BMP2	2857	0.02	0.02	0.02	0.02	0.02	0.03	0.02	0.02	0.04	0.05
IL12A	657	0.02	0.02	0.02	0.02	0.02	0.02	0.02	0.02	0.03	0.05
AGTR1	4219	0.02	0.02	0.02	0.01	0.02	0.02	0.03	0.03	0.03	0.08
PAX6	1661	0.02	0.02	0.02	0.02	0.03	0.02	0.02	0.02	0.03	0.04
IGSF4	1138	0.03	0.02	0.02	0.02	0.02	0.02	0.02	0.02	0.04	0.05
ITPR3	3533	0.02	0.02	0.02	0.02	0.03	0.02	0.02	0.02	0.04	0.05
TGFBR3	5095	0.02	0.03	0.02	0.03	0.02	0.02	0.03	0.02	0.03	0.03
APC	2259	0.04	0.02	0.02	0.02	0.03	0.02	0.02	0.02	0.05	0.04
HOXA9	3908	0.02	0.02	0.02	0.02	0.02	0.04	0.02	0.02	0.02	0.12
CCKBR	1753	0.02	0.02	0.02	0.02	0.02	0.02	0.02	0.02	0.04	0.06
RIPK3	2308	0.03	0.02	0.02	0.01	0.03	0.02	0.02	0.03	0.02	0.02
ABCG2	2985	0.03	0.02	0.02	0.02	0.02	0.02	0.02	0.02	0.03	0.05
CCND2	2328	0.02	0.02	0.02	0.03	0.03	0.03	0.02	0.02	0.03	0.02
TWIST1	3995	0.02	0.02	0.05	0.01	0.03	0.03	0.02	0.03	0.03	0.05
CTSL	5565	0.02	0.02	0.02	0.02	0.03	0.02	0.02	0.02	0.03	0.08
EDN1	696	0.02	0.02	0.02	0.02	0.02	0.02	0.02	0.02	0.03	0.03
IHH	5583	0.02	0.04	0.02	0.02	0.03	0.02	0.02	0.02	0.04	0.06
RASSF1	3865	0.02	0.03	0.02	0.03	0.03	0.02	0.02	0.02	0.02	0.02
FGF5	3734	0.02	0.03	0.02	0.03	0.02	0.02	0.02	0.02	0.04	0.09
NRAS	4900	0.02	0.02	0.03	0.02	0.02	0.03	0.02	0.02	0.02	0.02
NCL	1588	0.02	0.03	0.02	0.02	0.03	0.02	0.02	0.02	0.04	0.04
ACVR2B	5842	0.02	0.02	0.02	0.02	0.03	0.02	0.02	0.02	0.04	0.03
RRAS	5966	0.02	0.02	0.04	0.05	0.03	0.02	0.02	0.02	0.04	0.02
EVI1	2179	0.02	0.02	0.02	0.02	0.03	0.02	0.02	0.03	0.04	0.05
FRZB	4041	0.04	0.02	0.02	0.02	0.03	0.02	0.03	0.02	0.06	0.06
LIG4	2723	0.02	0.02	0.02	0.03	0.03	0.02	0.03	0.02	0.04	0.03
IGF1R	4144	0.02	0.02	0.02	0.02	0.02	0.02	0.02	0.02	0.05	0.03
CTNNA1	3503	0.02	0.02	0.02	0.04	0.03	0.03	0.02	0.02	0.03	0.03
GRB10	943	0.02	0.02	0.03	0.02	0.03	0.02	0.02	0.02	0.05	0.05
MAPK12	5655	0.03	0.02	0.02	0.05	0.03	0.02	0.02	0.02	0.03	0.04
PTPN6	3840	0.05	0.02	0.03	0.02	0.03	0.02	0.02	0.02	0.04	0.03
BCAM	3038	0.02	0.03	0.02	0.02	0.03	0.02	0.03	0.02	0.03	0.02
CD44	4049	0.03	0.02	0.02	0.02	0.03	0.03	0.02	0.02	0.06	0.06
ODC1	5104	0.02	0.03	0.03	0.02	0.02	0.02	0.02	0.02	0.03	0.02
CDKN1B	2375	0.03	0.02	0.01	0.01	0.02	0.02	0.02	0.03	0.02	0.03
HOXA11	1066	0.03	0.03	0.02	0.02	0.02	0.02	0.02	0.02	0.04	0.1
CCKBR	1756	0.02	0.02	0.02	0.03	0.03	0.03	0.02	0.02	0.03	0.02
TJP1	5268	0.02	0.02	0.03	0.03	0.03	0.02	0.02	0.02	0.04	0.05
GATA6	5874	0.02	0.02	0.04	0.02	0.03	0.02	0.02	0.02	0.02	0.12
MAF	4251	0.04	0.02	0.03	0.02	0.03	0.02	0.02	0.02	0.03	0.03
PROK2	1765	0.02	0.02	0.03	0.03	0.02	0.03	0.02	0.02	0.02	0.03
HIC1	1000	0.03	0.02	0.02	0.04	0.02	0.02	0.03	0.02	0.03	0.04
FLT1	3783	0.04	0.02	0.02	0.02	0.02	0.03	0.03	0.02	0.05	0.08
CAV2	5468	0.02	0.02	0.03	0.03	0.03	0.02	0.02	0.02	0.04	0.03
SYK	4178	0.02	0.02	0.03	0.02	0.03	0.03	0.02	0.02	0.03	0.06
MOS	2748	0.03	0.02	0.03	0.02	0.03	0.02	0.02	0.03	0.07	0.04
FGF3	5573	0.03	0.02	0.03	0.04	0.02	0.03	0.02	0.02	0.05	0.06
CCNA1	3049	0.02	0.03	0.02	0.02	0.03	0.03	0.02	0.02	0.04	0.07
EPHB2	2927	0.07	0.02	0.02	0.03	0.03	0.02	0.03	0.02	0.02	0.02
GAS1	1343	0.03	0.03	0.02	0.02	0.02	0.03	0.02	0.02	0.02	0.03
IL6	2721	0.02	0.02	0.03	0.03	0.02	0.03	0.02	0.03	0.03	0.03
EGFR	4086	0.02	0.02	0.03	0.02	0.04	0.03	0.02	0.03	0.04	0.07
GSTP1	4101	0.04	0.02	0.02	0.02	0.03	0.03	0.02	0.03	0.04	0.08
DHCR24	1777	0.03	0.03	0.02	0.02	0.03	0.03	0.02	0.02	0.03	0.03
EPHB6	1874	0.03	0.02	0.02	0.03	0.03	0.02	0.03	0.02	0.05	0.04
VIM	5982	0.04	0.02	0.02	0.02	0.03	0.03	0.02	0.03	0.04	0.04
ABCC5	27	0.02	0.02	0.03	0.02	0.03	0.03	0.02	0.03	0.03	0.04
TCF7L2	5054	0.04	0.03	0.02	0.02	0.03	0.02	0.03	0.02	0.03	0.02
NTRK3	4957	0.03	0.02	0.04	0.04	0.02	0.03	0.02	0.03	0.04	0.04
NTRK3	991	0.02	0.02	0.03	0.05	0.03	0.03	0.03	0.02	0.03	0.03
TMEFF2	6131	0.03	0.03	0.02	0.02	0.03	0.03	0.03	0.03	0.04	0.04
TIMP2	1317	0.03	0.02	0.02	0.04	0.03	0.03	0.03	0.02	0.03	0.02

SYMBOL	PROBE ID	SAMPLE										IMP	
		A	B	C	D	E	F	G	S	1	2	MI	PI
RAB32	1804	0.012	0.011	0.014	0.012	0.017	0.013	0.011	0.011	0.021	0.140		
DBC1	629	0.018	0.011	0.014	0.013	0.012	0.022	0.012	0.012	0.060	0.204		
ESR1	2461	0.026	0.013	0.012	0.032	0.015	0.013	0.015	0.012	0.076	0.244		
IGFBP5	4145	0.015	0.027	0.013	0.014	0.020	0.019	0.015	0.017	0.103	0.216		
HGF	2755	0.038	0.020	0.023	0.009	0.016	0.016	0.013	0.017	0.151	0.371		
COL1A2	315	0.016	0.017	0.016	0.014	0.019	0.017	0.018	0.020	0.038	0.123		
CDH13	4061	0.027	0.016	0.017	0.016	0.022	0.017	0.017	0.019	0.057	0.146		
MATK	5744	0.017	0.018	0.027	0.022	0.017	0.020	0.013	0.016	0.033	0.171		
ERBB3	2458	0.018	0.030	0.027	0.017	0.023	0.017	0.019	0.018	0.090	0.180		
TNK1	5291	0.018	0.034	0.046	0.018	0.021	0.019	0.019	0.018	0.105	0.342		
PITX2	6125	0.038	0.028	0.020	0.016	0.016	0.019	0.017	0.022	0.327	0.145		
NEO1	4885	0.017	0.022	0.027	0.018	0.019	0.024	0.018	0.020	0.078	0.120		
POMC	1743	0.022	0.017	0.019	0.018	0.022	0.019	0.020	0.021	0.098	0.197		
RBL2	5132	0.033	0.022	0.021	0.017	0.019	0.020	0.022	0.019	0.068	0.093		
FAT	4000	0.026	0.022	0.021	0.018	0.027	0.020	0.021	0.018	0.063	0.168		
DNAJC15	3155	0.027	0.022	0.021	0.017	0.026	0.022	0.020	0.022	0.080	0.136		
GSTM1	4902	0.043	0.023	0.019	0.019	0.026	0.022	0.021	0.017	0.182	0.238		
IL6	2717	0.026	0.023	0.016	0.021	0.025	0.021	0.023	0.021	0.058	0.117		
LMO1	813	0.033	0.023	0.017	0.015	0.032	0.022	0.017	0.022	0.076	0.133		
HOXA5	1072	0.038	0.027	0.022	0.023	0.023	0.020	0.020	0.019	0.078	0.418		
BMPR1A	1899	0.023	0.022	0.020	0.021	0.022	0.022	0.022	0.021	0.042	0.151		
FGFR1	4033	0.024	0.021	0.026	0.033	0.023	0.023	0.017	0.022	0.071	0.088		
CDH11	3237	0.018	0.023	0.023	0.018	0.028	0.024	0.024	0.023	0.038	0.125		
DCC	2425	0.038	0.023	0.017	0.041	0.024	0.018	0.028	0.018	0.160	0.251		
CD44	2330	0.032	0.021	0.029	0.018	0.022	0.030	0.025	0.022	0.032	0.185		
SMO	5241	0.046	0.020	0.021	0.024	0.028	0.021	0.027	0.023	0.059	0.106		
TSP50	2158	0.055	0.023	0.031	0.017	0.024	0.025	0.018	0.017	0.239	0.406		
DIO3	636	0.024	0.021	0.030	0.027	0.027	0.023	0.020	0.019	0.054	0.220		
IRF5	3518	0.055	0.021	0.022	0.019	0.025	0.023	0.027	0.025	0.098	0.204		
TAL1	5221	0.026	0.023	0.024	0.027	0.031	0.024	0.022	0.022	0.077	0.144		
TFAP2C	1623	0.055	0.048	0.025	0.038	0.024	0.023	0.025	0.015	0.109	0.132		
CFTR	247	0.034	0.022	0.024	0.023	0.028	0.043	0.026	0.024	0.036	0.131		
HDAC1	5786	0.068	0.029	0.023	0.030	0.028	0.018	0.023	0.016	0.126	0.183		
SEMA3C	2331	0.026	0.022	0.026	0.019	0.024	0.040	0.025	0.030	0.102	0.124		
SLIT2	5211	0.023	0.034	0.030	0.026	0.027	0.030	0.025	0.025	0.046	0.289		
FGF12	3722	0.027	0.025	0.029	0.019	0.029	0.032	0.024	0.026	0.056	0.123		
ADCYAP1	42	0.027	0.031	0.026	0.022	0.029	0.027	0.026	0.024	0.065	0.111		
ABCA1	4214	0.042	0.023	0.025	0.029	0.027	0.029	0.026	0.024	0.041	0.115		
SOX17	5958	0.047	0.026	0.023	0.026	0.026	0.027	0.036	0.032	0.202	0.509		
SEZ6L	1931	0.039	0.026	0.025	0.027	0.031	0.025	0.028	0.026	0.042	0.148		
LOX	1311	0.025	0.025	0.031	0.023	0.051	0.044	0.023	0.028	0.053	0.258		
SOX2	5959	0.041	0.020	0.024	0.027	0.024	0.028	0.030	0.027	0.052	0.180		
EPHA3	3239	0.023	0.026	0.031	0.024	0.028	0.037	0.028	0.021	0.051	0.295		
ERG	312	0.034	0.038	0.025	0.022	0.028	0.037	0.026	0.020	0.053	0.157		
HTR1B	1104	0.032	0.028	0.027	0.023	0.034	0.028	0.030	0.026	0.045	0.492		
MYCL1	2798	0.027	0.029	0.023	0.059	0.029	0.030	0.024	0.023	0.047	0.231		
CTSL	5548	0.051	0.028	0.037	0.022	0.030	0.027	0.024	0.028	0.280	0.440		
IGF2R	2692	0.028	0.025	0.030	0.023	0.034	0.028	0.027	0.029	0.076	0.101		
PRKCDBP	3824	0.030	0.029	0.025	0.027	0.038	0.031	0.031	0.027	0.042	0.122		
PENK	1678	0.043	0.029	0.031	0.024	0.026	0.030	0.033	0.026	0.097	0.171		
MYOD1	1565	0.030	0.030	0.027	0.026	0.032	0.034	0.038	0.025	0.103	0.205		
ISL1	4974	0.049	0.018	0.040	0.028	0.030	0.032	0.026	0.030	0.070	0.219		
PLXDC2	3051	0.031	0.031	0.029	0.027	0.034	0.036	0.033	0.029	0.043	0.140		
HS3ST2	3482	0.037	0.032	0.023	0.053	0.030	0.035	0.030	0.026	0.075	0.330		
NPR2	2439	0.044	0.032	0.028	0.032	0.038	0.031	0.029	0.030	0.097	0.333		
SLC22A3	1985	0.039	0.025	0.033	0.029	0.034	0.030	0.028	0.035	0.429	0.204		
MDS1	799	0.034	0.031	0.029	0.032	0.032	0.036	0.030	0.033	0.099	0.100		
CRK	3392	0.085	0.032	0.052	0.032	0.028	0.027	0.041	0.019	0.157	0.331		
IGFBP6	4176	0.030	0.036	0.029	0.025	0.033	0.035	0.031	0.037	0.132	0.262		
MEST	3648	0.057	0.027	0.028	0.032	0.034	0.034	0.033	0.029	0.069	0.167		
ATP10A	109	0.032	0.047	0.029	0.037	0.047	0.033	0.031	0.031	0.189	0.258		
KIAA1804	2039	0.033	0.033	0.037	0.028	0.033	0.033	0.027	0.026	0.048	0.177		
DES	5517	0.069	0.032	0.035	0.033	0.033	0.033	0.033	0.032	0.092	0.167		
CD9	5546	0.043	0.027	0.032	0.037	0.031	0.054	0.036	0.030	0.138	0.271		
CALCA	178	0.042	0.034	0.034	0.047	0.034	0.057	0.033	0.024	0.063	0.102		
ABL1	2249	0.049	0.032	0.024	0.038	0.036	0.027	0.037	0.028	0.042	0.133		
PALM2-AKAP2	5114	0.035	0.028	0.037	0.024	0.034	0.040	0.035	0.030	0.051	0.118		
EVI1	2960	0.034	0.034	0.030	0.031	0.055	0.035	0.040	0.037	0.107	0.270		
PEG10	1671	0.041	0.054	0.049	0.018	0.025	0.025	0.038	0.031	0.169	0.327		
IL17RB	3510	0.033	0.040	0.036	0.033	0.038	0.036	0.034	0.032	0.084	0.318		
GRB7	1375	0.055	0.031	0.042	0.029	0.037	0.039	0.030	0.032	0.205	0.524		
ERN1	753	0.065	0.033	0.033	0.061	0.035	0.034	0.043	0.035	0.303	0.275		
RUNX1T1	5716	0.042	0.056	0.029	0.054	0.033	0.030	0.038	0.029	0.135	0.383		
CSPG2	4073	0.050	0.029	0.028	0.021	0.045	0.042	0.045	0.024	0.033	0.144		
IGSF4	1125	0.058	0.043	0.043	0.028	0.030	0.037	0.035	0.034	0.049	0.123		
MYOD1	3697	0.047	0.032	0.035	0.031	0.039	0.039	0.031	0.037	0.081	0.246		
CDH13	2356	0.050	0.039	0.035	0.034	0.036	0.044	0.036	0.034	0.126	0.327		
CD82	1967	0.034	0.042	0.026	0.041	0.037	0.036	0.040	0.036	0.172	0.334		
RIPK4	2906	0.053	0.034	0.024	0.041	0.040	0.035	0.038	0.026	0.086	0.125		
ONECUT2	1768	0.037	0.033	0.041	0.043	0.044	0.036	0.034	0.037	0.065	0.171		
GABRB3	3299	0.031	0.037	0.038	0.084	0.046	0.040	0.036	0.026	0.060	0.179		
VAV2	2822	0.038	0.039	0.051	0.076	0.037	0.031	0.032	0.033	0.073	0.093		
FABP3	3283	0.073	0.038	0.035	0.033	0.040	0.037	0.040	0.033	0.215	0.243		
NES	5911	0.063	0.060	0.036	0.036	0.048	0.041	0.035	0.035	0.130	0.342		
AGTR1	4222	0.046	0.034	0.036	0.033	0.041	0.044	0.038	0.041	0.038	0.240		
SEMA3F	1032	0.060	0.040	0.053	0.037	0.048	0.039	0.036	0.029	0.132	0.121		
TYRO3	5236	0.050	0.037	0.072	0.048	0.043	0.034	0.033	0.031	0.111	0.303		
HHIP	2230	0.060	0.039	0.205	0.030	0.038	0.042	0.041	0.025	0.299	0.271		
MOS	4133	0.027	0.037	0.039	0.060	0.034	0.042	0.067	0.041	0.081	0.328		
TNFRSF10C	2143	0.035	0.042	0.039	0.032	0.046	0.042	0.037	0.044	0.059	0.101		
EPHB6	2715	0.052	0.040	0.041	0.040	0.041	0.038	0.038	0.045	0.086	0.079		
JAK3	5615	0.040	0.058	0.049	0.072	0.040	0.042	0.032	0.029	0.066	0.097		
BMP4	3050	0.063	0.063	0.043	0.035	0.039	0.055	0.038	0.028	0.233	0.384		
HOXA9	4107	0.047	0.041	0.032	0.043	0.050	0.039	0.043	0.029	0.051	0.298		
SLC5A8	2003	0.030	0.048	0.032	0.021	0.064	0.038	0.050	0.045	0.069	0.118		
ISL1	4969	0.041	0.046	0.035	0.031	0.044	0.043	0.041	0.054	0.045	0.152		
MAP2K6	5050	0.048	0.047	0.051	0.029	0.043	0.042	0.036	0.034	0.366	0.179		
CALCA	3043	0.045	0.040	0.048	0.036	0.049	0.045	0.039	0.041	0.113	0.332		
FES	4002	0.063	0.042	0.038	0.042	0.051	0.038	0.055	0.044	0.080	0.134		
ZNF215	2216	0.032	0.047	0.033	0.049	0.047	0.035	0.039	0.049	0.159	0.125		
GPX1	4901	0.052	0.037	0.043	0.049	0.047	0.042	0.041	0.044	0.174	0.156		
CD40	5537	0.041	0.030	0.036	0.038	0.050	0.049	0.046	0.053	0.088	0.112		
SHH	1739	0.097	0.040	0.042	0.047	0.040	0.059	0.045	0.043	0.153	0.323		
FAS	4870	0.033	0.056	0.048	0.037	0.042	0.053	0.046	0.037	0.101	0.063		
PPARD	4278	0.050	0.036	0.049	0.037	0.057	0.038	0.045	0.043	0.135	0.174		
IMPACT	1191	0.044	0.037	0.060	0.033	0.045	0.048	0.044	0.049	0.059	0.149		
EYA4	3279	0.037	0.043	0.053	0.050	0.038	0.045	0.060	0.044	0.102	0.080		
KCNK4	3534	0.077	0.060	0.044	0.042	0.047	0.036	0.060	0.040	0.193	0.511		
TIMP3	6126	0.064	0.030	0.052	0.054	0.039	0.036	0.052	0.029	0.129	0.098		
COL1A1	3253	0.115	0.053	0.047	0.039	0.070	0.043	0.045	0.039	0.141	0.470		
DBC1	3141	0.053	0.054	0.039	0.053	0.047	0.045	0.041	0.033	0.136	0.351		
HLA-DRA	1044	0.035	0.026	0.056	0.047	0.046	0.038	0.071	0.054	0.084	0.076		
EFNB3	1828	0.043	0.053	0.054	0.046	0.047	0.048	0.039	0.033	0.059	0.099		
GSTM2	946	0.061	0.038	0.043	0.057	0.047	0.049	0.048	0.038	0.064	0.123		
RET	6099	0.034	0.050	0.038	0.056	0.046	0.057	0.056	0.039	0.071	0.089		

SYMBOL	PROBE ID	SAMPLE										IMP	
		A	B	C	D	E	F	G	S	1	2	MI	PI
OGG1	936	0.08	0.02	0.02	0.02	0.02	0.02	0.01	0.01	0.37	0.77		
TSC2	4202	0.09	0.02	0.03	0.02	0.02	0.02	0.02	0.01	0.52	0.73		
EPHA2	2081	0.06	0.03	0.03	0.02	0.02	0.02	0.02	0.02	0.34	0.48		
PPARG	4993	0.1	0.03	0.03	0.03	0.02	0.02	0.02	0.02	0.89	0.61		
MET	805	0.07	0.03	0.02	0.02	0.03	0.02	0.03	0.02	0.55	0.62		
MST1R	4162	0.18	0.04	0.04	0.02	0.03	0.02	0.02	0.02	0.55	0.8		
ALOX12	75	0.13	0.03	0.02	0.03	0.02	0.03	0.03	0.02	0.48	0.83		
FER	3706	0.11	0.04	0.04	0.02	0.03	0.02	0.03	0.03	0.88	0.9		
MEG3	1384	0.32	0.06	0.07	0.02	0.03	0.02	0.03	0.03	0.83	0.83		■
ELL	3515	0.06	0.04	0.03	0.03	0.04	0.02	0.02	0.03	0.54	0.51		
DDIT3	3712	0.05	0.03	0.03	0.03	0.04	0.03	0.03	0.03	0.59	0.61		
SGCE	1969	0.36	0.08	0.08	0.02	0.03	0.02	0.03	0.03	0.86	0.87	■	
SIN3B	2807	0.22	0.04	0.06	0.04	0.02	0.02	0.03	0.03	0.91	0.91		
NBL1	1581	0.17	0.03	0.04	0.04	0.03	0.02	0.03	0.02	0.89	0.84		
BMPR1A	2440	0.24	0.06	0.08	0.04	0.03	0.03	0.03	0.03	0.8	0.83		
SEPT5	5988	0.09	0.04	0.04	0.04	0.03	0.03	0.03	0.03	0.44	0.53		
RARRES1	1824	0.18	0.05	0.05	0.03	0.03	0.03	0.03	0.04	0.75	0.87		
GSTM2	944	0.07	0.04	0.05	0.03	0.03	0.04	0.03	0.03	0.46	0.6		
WRN	5444	0.22	0.05	0.05	0.03	0.03	0.03	0.04	0.03	0.92	0.93		
ZIM2	2198	0.12	0.05	0.04	0.04	0.04	0.03	0.03	0.02	0.62	0.69		
DDR1	4303	0.14	0.05	0.05	0.04	0.04	0.03	0.03	0.03	0.84	0.85		
GML	5741	0.33	0.06	0.07	0.03	0.04	0.03	0.04	0.03	0.95	0.94		
HOXA5	3470	0.26	0.07	0.07	0.03	0.04	0.02	0.04	0.02	0.48	0.88		
IGFBP1	4111	0.06	0.04	0.05	0.05	0.03	0.03	0.03	0.03	0.38	0.45		
NBL1	3704	0.28	0.05	0.06	0.05	0.03	0.03	0.02	0.02	0.92	0.87		
TM7SF3	2122	0.04	0.04	0.05	0.03	0.05	0.02	0.04	0.03	0.54	0.77		
MAP3K8	4067	0.14	0.04	0.04	0.05	0.04	0.03	0.04	0.02	0.55	0.83		
IGFBP1	2698	0.06	0.06	0.05	0.04	0.04	0.04	0.04	0.04	0.2	0.54		
DNAJC15	656	0.27	0.08	0.08	0.04	0.04	0.03	0.04	0.03	0.93	0.91		
ALOX12	3001	0.1	0.04	0.05	0.04	0.04	0.03	0.05	0.03	0.59	0.8		
CCKAR	2459	0.12	0.04	0.04	0.05	0.03	0.03	0.03	0.05	0.92	0.87		
SIN3B	2816	0.36	0.08	0.09	0.05	0.04	0.04	0.03	0.03	0.94	0.94		
FANCA	3977	0.17	0.05	0.05	0.04	0.04	0.04	0.04	0.03	0.9	0.84		
TRIM29	2394	0.2	0.05	0.04	0.03	0.04	0.04	0.03	0.03	0.96	0.96		
PLAGL1	1689	0.2	0.07	0.06	0.05	0.04	0.04	0.04	0.03	0.71	0.68	■	
MAGEL2	1339	0.25	0.05	0.05	0.03	0.07	0.04	0.03	0.03	0.83	0.88		
FGFR3	2536	0.15	0.04	0.05	0.06	0.03	0.04	0.04	0.04	0.19	0.57		
SNURF	2035	0.18	0.05	0.04	0.04	0.04	0.04	0.05	0.04	0.75	0.73	■	
XPC	5297	0.37	0.12	0.09	0.05	0.03	0.03	0.04	0.03	0.86	0.85		
ZNF264	2225	0.09	0.04	0.04	0.07	0.05	0.04	0.05	0.03	0.68	0.82		
E2F5	5602	0.2	0.04	0.07	0.05	0.05	0.04	0.05	0.03	0.41	0.55		
SNURF	2037	0.14	0.04	0.05	0.04	0.05	0.05	0.05	0.04	0.54	0.53	■	
DIRAS3	5576	0.15	0.1	0.07	0.04	0.05	0.03	0.04	0.05	0.75	0.75		
CYP2E1	5510	0.29	0.18	0.07	0.04	0.05	0.04	0.04	0.04	0.93	0.81		
LIMK1	5035	0.21	0.07	0.08	0.03	0.06	0.03	0.04	0.04	0.92	0.92		
CHGA	285	0.09	0.05	0.06	0.04	0.05	0.04	0.04	0.05	0.57	0.32		
MEG3	3640	0.13	0.08	0.06	0.04	0.05	0.05	0.05	0.03	0.51	0.32		■
LIG3	4038	0.3	0.07	0.06	0.03	0.04	0.05	0.05	0.03	0.92	0.81		
GPATC3	929	0.1	0.09	0.05	0.06	0.03	0.05	0.05	0.03	0.23	0.61		
SNRPN	6123	0.17	0.05	0.05	0.04	0.05	0.03	0.04	0.06	0.67	0.68	■	
GSTM1	4908	0.12	0.08	0.1	0.05	0.05	0.04	0.03	0.04	0.7	0.71		
BRCA1	2318	0.13	0.05	0.05	0.06	0.04	0.03	0.06	0.03	0.89	0.74		
IPF1	5893	0.06	0.08	0.04	0.05	0.04	0.05	0.06	0.04	0.27	0.49		
EPHA2	2062	0.08	0.07	0.04	0.06	0.06	0.05	0.05	0.03	0.34	0.65		
CD81	220	0.11	0.06	0.06	0.06	0.05	0.05	0.05	0.04	0.48	0.76		
AXIN1	4229	0.12	0.05	0.06	0.05	0.06	0.06	0.04	0.05	0.24	0.58		
PITX2	1771	0.06	0.05	0.05	0.05	0.06	0.08	0.04	0.06	0.45	0.35		
DIO3	3145	0.24	0.07	0.07	0.05	0.05	0.05	0.05	0.06	0.91	0.86		
MAGEL2	3624	0.2	0.06	0.08	0.05	0.09	0.05	0.05	0.05	0.88	0.81		
PLAGL1	3780	0.19	0.05	0.08	0.04	0.04	0.07	0.06	0.05	0.75	0.79	■	
LIF	4029	0.13	0.05	0.1	0.1	0.06	0.04	0.06	0.05	0.63	0.32		
HIC1	6059	0.06	0.1	0.04	0.11	0.06	0.06	0.05	0.05	0.15	0.7		
ZIM2	4009	0.16	0.06	0.06	0.11	0.06	0.07	0.05	0.06	0.67	0.68		
EMR3	740	0.33	0.08	0.1	0.06	0.06	0.04	0.04	0.04	0.88	0.85		
CTSD	3523	0.18	0.09	0.09	0.06	0.06	0.05	0.05	0.04	0.6	0.54		
ATP10A	110	0.09	0.06	0.08	0.06	0.06	0.07	0.05	0.05	0.74	0.73		
NNAT	1634	0.36	0.08	0.09	0.06	0.06	0.06	0.05	0.06	0.77	0.9	■	
RAN	4881	0.23	0.08	0.1	0.04	0.07	0.05	0.05	0.06	0.47	0.66		
GRB7	5759	0.09	0.09	0.06	0.06	0.06	0.05	0.07	0.05	0.31	0.58		
FZD9	5551	0.09	0.05	0.07	0.06	0.08	0.07	0.06	0.05	0.5	0.4		
SMARCB1	2354	0.18	0.07	0.08	0.06	0.06	0.06	0.04	0.03	0.69	0.51		
SNRPN	6124	0.23	0.08	0.08	0.06	0.06	0.07	0.05	0.06	0.8	0.74	■	
RAD50	2834	0.26	0.1	0.07	0.05	0.07	0.07	0.05	0.05	0.54	0.79		
SEPT5	5997	0.19	0.07	0.07	0.06	0.07	0.07	0.06	0.06	0.58	0.7		
DLC1	2431	0.12	0.07	0.07	0.07	0.07	0.05	0.07	0.07	0.5	0.27		
TNK1	5293	0.16	0.08	0.08	0.05	0.06	0.03	0.07	0.04	0.48	0.57		
BCR	2304	0.09	0.07	0.1	0.05	0.06	0.08	0.07	0.06	0.48	0.57		
TRIM29	2952	0.45	0.12	0.09	0.07	0.07	0.06	0.06	0.06	0.78	0.93		
ITGA6	2277	0.51	0.11	0.18	0.08	0.06	0.05	0.04	0.05	0.96	0.96		
SERPINB5	1908	0.33	0.1	0.13	0.07	0.06	0.06	0.07	0.07	0.97	0.97		
HOXB2	5880	0.18	0.08	0.09	0.06	0.08	0.04	0.06	0.06	0.79	0.87		
GML	1628	0.41	0.15	0.07	0.07	0.07	0.06	0.06	0.06	0.94	0.91		
CD9	5853	0.09	0.06	0.07	0.09	0.07	0.07	0.09	0.05	0.36	0.55		
NKX3-1	1617	0.58	0.14	0.22	0.07	0.07	0.07	0.05	0.06	0.95	0.95		
KRAS	3561	0.12	0.07	0.08	0.07	0.06	0.07	0.05	0.06	0.66	0.68		
MST1R	881	0.15	0.08	0.07	0.07	0.08	0.07	0.07	0.05	0.78	0.74		
DSC2	2038	0.43	0.14	0.12	0.08	0.07	0.07	0.07	0.04	0.88	0.9		
CALCA	174	0.13	0.07	0.11	0.07	0.08	0.06	0.09	0.05	0.57	0.5		
PPAT	3810	0.14	0.08	0.09	0.06	0.08	0.08	0.07	0.08	0.68	0.55		
XRCC1	5328	0.39	0.1	0.11	0.08	0.07	0.05	0.05	0.05	0.88	0.89		
PHLDA2	1682	0.11	0.1	0.05	0.08	0.08	0.1	0.07	0.05	0.59	0.3		
CYP2E1	4300	0.39	0.09	0.15	0.05	0.09	0.07	0.05	0.07	0.95	0.93		
SNURF	3937	0.27	0.09	0.1	0.06	0.07	0.08	0.08	0.07	0.7	0.81		
TSP50	3987	0.22	0.11	0.1	0.09	0.08	0.07	0.07	0.06	0.72	0.82		
CCKAR	1749	0.31	0.07	0.15	0.1	0.07	0.06	0.08	0.08	0.92	0.87		
HOXA5	1071	0.36	0.09	0.09	0.05	0.08	0.04	0.05	0.08	0.65	0.85		
HOXB2	5879	0.07	0.11	0.1	0.07	0.1	0.08	0.11	0.06	0.4	0.57		
DKFZP564O0823	2027	0.09	0.09	0.08	0.08	0.08	0.09	0.08	0.09	0.52	0.4		
CDK10	3285	0.13	0.09	0.09	0.1	0.08	0.08	0.09	0.07	0.75	0.48		
TGFBI	5091	0.11	0.07	0.09	0.12	0.09	0.09	0.08	0.11	0.29	0.47		
MAD2L1	3606	0.35	0.11	0.1	0.1	0.09	0.06	0.08	0.04	0.77	0.75		
MMP14	4148	0.13	0.11	0.09	0.13	0.1	0.07	0.09	0.08	0.38	0.73		
SLC22A3	1987	0.2	0.11	0.11	0.1	0.07	0.09	0.13	0.08	0.69	0.6		
HRASLS	1086	0.27	0.14	0.07	0.07	0.12	0.1	0.11	0.09	0.68	0.61		
UGT1A1	2406	0.39	0.21	0.15	0.08	0.09	0.07	0.12	0.08	0.91	0.88		
TRIM29	2398	0.42	0.15	0.13	0.09	0.08	0.07	0.11	0.1	0.92	0.85		
GNAS	3330	0.3	0.12	0.17	0.08	0.1	0.07	0.12	0.08	0.84	0.82		■
ASCL2	105	0.11	0.11	0.11	0.13	0.11	0.11	0.07	0.06	0.37	0.38		
SLC22A2	3924	0.37	0.08	0.14	0.23	0.07	0.05	0.34	0.06	0.97	0.95		
GLI3	630	0.45	0.17	0.18	0.1	0.11	0.07	0.12	0.09	0.94	0.94		
KLK11	4997	0.22	0.11	0.13	0.11	0.06	0.07	0.07	0.17	0.89	0.76		
COL1A2	331	0.15	0.13	0.09	0.07	0.1	0.13	0.13	0.09	0.33	0.46		
SNRPN	3936	0.36	0.12	0.15	0.1	0.13	0.11	0.12	0.08	0.61	0.86		
DIRAS3	1307	0.35	0.17	0.17	0.11	0.09	0.1	0.12	0.06	0.85	0.86	■	
GNAS	915	0.22	0.1	0.15	0.11	0.13	0.13	0.12	0.11	0.7	0.74		■

LOX	1309	0.02	0.03	0.02	0.02	0.03	0.03	0.03	0.02	0.05	0.05
PDGFA	5930	0.02	0.04	0.02	0.02	0.03	0.03	0.03	0.02	0.02	0.04
CD40	1558	0.03	0.03	0.02	0.02	0.03	0.03	0.02	0.03	0.04	0.05
LIF	743	0.03	0.02	0.02	0.04	0.03	0.03	0.03	0.02	0.04	0.03
BIRC5	54	0.11	0.02	0.08	0.02	0.03	0.02	0.03	0.02	0.03	0.04
TFPI2	2097	0.03	0.03	0.02	0.02	0.03	0.03	0.03	0.03	0.05	0.05
SKI	1604	0.03	0.03	0.03	0.03	0.03	0.02	0.03	0.02	0.03	0.03
ARNT	3022	0.04	0.03	0.03	0.02	0.03	0.03	0.02	0.03	0.04	0.03
COL4A3	125	0.02	0.02	0.02	0.03	0.03	0.03	0.03	0.02	0.04	0.07
FANCF	791	0.02	0.03	0.02	0.02	0.03	0.04	0.03	0.02	0.05	0.05
ICAM1	2651	0.03	0.03	0.03	0.02	0.04	0.03	0.03	0.02	0.04	0.03
DUSP4	1808	0.04	0.03	0.02	0.02	0.03	0.03	0.02	0.02	0.02	0.03
YES1	5452	0.03	0.03	0.02	0.03	0.03	0.03	0.03	0.02	0.03	0.02
CSF1	433	0.04	0.02	0.02	0.03	0.03	0.04	0.03	0.02	0.05	0.03
PCDH1	2856	0.02	0.04	0.03	0.03	0.03	0.03	0.03	0.02	0.04	0.04
DAPK1	2421	0.02	0.03	0.05	0.02	0.03	0.03	0.03	0.03	0.05	0.08
ZP3	4018	0.03	0.03	0.03	0.03	0.03	0.03	0.03	0.03	0.04	0.03
EGR4	720	0.02	0.03	0.03	0.03	0.03	0.03	0.03	0.03	0.03	0.03
IGFBP3	2707	0.02	0.03	0.03	0.03	0.03	0.03	0.03	0.02	0.05	0.09
RET	2861	0.03	0.03	0.02	0.02	0.03	0.03	0.03	0.02	0.08	0.06
RASA1	1697	0.05	0.02	0.02	0.04	0.03	0.02	0.03	0.03	0.03	0.04
CREB1	4297	0.02	0.03	0.02	0.03	0.03	0.03	0.02	0.03	0.1	0.04
RAB32	3857	0.02	0.03	0.03	0.02	0.03	0.04	0.03	0.03	0.04	0.07
HOXA11	3468	0.03	0.03	0.03	0.01	0.03	0.02	0.03	0.03	0.05	0.09
IGFBP7	1111	0.03	0.03	0.03	0.02	0.03	0.03	0.02	0.03	0.04	0.03
RARB	4165	0.03	0.03	0.03	0.02	0.03	0.03	0.03	0.03	0.03	0.05
FHIT	1887	0.03	0.02	0.02	0.02	0.03	0.03	0.03	0.03	0.03	0.06
LMO1	4247	0.03	0.03	0.03	0.03	0.03	0.03	0.03	0.03	0.03	0.03
TUBB3	3990	0.1	0.02	0.04	0.02	0.03	0.02	0.02	0.08	0.04	0.05
APBA1	56	0.03	0.03	0.03	0.03	0.03	0.03	0.03	0.03	0.04	0.05
TMEFF1	3979	0.03	0.03	0.03	0.03	0.04	0.03	0.03	0.03	0.03	0.03
NOTCH2	4206	0.03	0.04	0.03	0.03	0.02	0.03	0.07	0.03	0.02	0.02
CASP6	4259	0.02	0.03	0.03	0.02	0.03	0.03	0.05	0.03	0.06	0.08
PODXL	5925	0.03	0.03	0.02	0.05	0.03	0.04	0.03	0.03	0.04	0.08
TNFRSF10C	3981	0.03	0.02	0.03	0.02	0.05	0.05	0.02	0.03	0.05	0.05
PGF	1024	0.05	0.03	0.03	0.03	0.03	0.03	0.03	0.03	0.06	0.07
TGFB2	5074	0.03	0.03	0.03	0.03	0.03	0.03	0.03	0.03	0.04	0.04
MMP2	4307	0.02	0.05	0.02	0.04	0.03	0.03	0.02	0.04	0.03	0.09
TNFRSF10A	5148	0.03	0.03	0.03	0.03	0.04	0.04	0.03	0.03	0.05	0.03
TUBB3	2162	0.05	0.03	0.03	0.04	0.03	0.03	0.03	0.03	0.04	0.03
GNMT	3338	0.03	0.03	0.03	0.03	0.04	0.04	0.03	0.03	0.04	0.12
MLLT4	4134	0.03	0.03	0.03	0.03	0.03	0.03	0.03	0.03	0.04	0.11
ADAMTS12	40	0.03	0.03	0.03	0.03	0.04	0.02	0.03	0.02	0.03	0.05
LRRC32	2087	0.05	0.03	0.03	0.02	0.04	0.03	0.03	0.03	0.03	0.03
VIM	5981	0.02	0.04	0.03	0.03	0.03	0.04	0.02	0.03	0.03	0.03
ACVR1B	5834	0.02	0.03	0.02	0.03	0.03	0.03	0.02	0.04	0.03	0.06
FGFR2	2485	0.03	0.03	0.03	0.04	0.03	0.03	0.03	0.03	0.04	0.05
FZD7	1600	0.04	0.03	0.03	0.03	0.03	0.03	0.03	0.03	0.04	0.04
FLT1	3789	0.03	0.03	0.03	0.02	0.03	0.03	0.03	0.02	0.05	0.04
COL4A3	3280	0.02	0.03	0.03	0.03	0.03	0.03	0.03	0.03	0.05	0.06
ITGA2	2789	0.03	0.02	0.02	0.02	0.04	0.03	0.03	0.03	0.04	0.04
EPHB3	2160	0.05	0.02	0.03	0.02	0.04	0.04	0.03	0.03	0.05	0.09
YES1	5357	0.02	0.03	0.03	0.04	0.03	0.03	0.04	0.02	0.02	0.02
IGFBP5	717	0.04	0.03	0.03	0.04	0.03	0.02	0.03	0.03	0.06	0.09
TNC	5285	0.06	0.02	0.03	0.02	0.03	0.03	0.03	0.02	0.04	0.12
CYP1A1	2416	0.03	0.03	0.02	0.02	0.05	0.03	0.03	0.03	0.06	0.06
PLXDC2	2776	0.04	0.02	0.03	0.03	0.03	0.05	0.03	0.04	0.04	0.07
TGFA	2892	0.02	0.03	0.02	0.03	0.03	0.03	0.04	0.03	0.04	0.03
PPP2R1B	2222	0.02	0.03	0.06	0.03	0.03	0.04	0.03	0.03	0.05	0.04
AHR	28	0.03	0.03	0.04	0.02	0.03	0.03	0.03	0.03	0.04	0.05
CCND2	2329	0.04	0.02	0.03	0.04	0.04	0.03	0.03	0.03	0.05	0.03
PRKAR1A	2791	0.04	0.02	0.04	0.03	0.04	0.03	0.03	0.03	0.04	0.06
TGFBR3	1085	0.03	0.03	0.02	0.03	0.04	0.05	0.04	0.03	0.03	0.04
GALR1	3314	0.03	0.04	0.03	0.03	0.04	0.04	0.03	0.03	0.04	0.04
KIT	4019	0.03	0.03	0.03	0.04	0.03	0.03	0.03	0.03	0.06	0.06
NTRK3	4224	0.07	0.05	0.02	0.05	0.03	0.03	0.03	0.03	0.03	0.06
GUCY2D	2999	0.04	0.03	0.03	0.04	0.04	0.03	0.03	0.03	0.05	0.08
CDKN2A	6140	0.04	0.03	0.04	0.03	0.04	0.03	0.03	0.03	0.05	0.08
MAPK12	5060	0.09	0.02	0.04	0.04	0.03	0.03	0.02	0.03	0.04	0.06
DLL1	4325	0.03	0.03	0.03	0.05	0.03	0.04	0.03	0.03	0.04	0.03
MYCN	2802	0.03	0.03	0.03	0.03	0.04	0.04	0.03	0.04	0.04	0.04
MEST	1390	0.07	0.03	0.04	0.02	0.03	0.03	0.03	0.03	0.04	0.04
DAB2	3377	0.03	0.03	0.03	0.03	0.04	0.03	0.03	0.04	0.05	0.04
PAX6	1665	0.03	0.03	0.03	0.04	0.04	0.03	0.04	0.03	0.05	0.1
TMEFF1	2129	0.03	0.03	0.06	0.07	0.03	0.03	0.05	0.03	0.05	0.08
SFRP1	1946	0.04	0.05	0.03	0.03	0.03	0.03	0.03	0.04	0.04	0.07
CDK10	182	0.03	0.02	0.02	0.08	0.04	0.03	0.06	0.02	0.03	0.03
TCF7L2	1060	0.03	0.03	0.03	0.03	0.04	0.03	0.04	0.03	0.05	0.04
POMC	1741	0.03	0.03	0.03	0.03	0.04	0.04	0.03	0.04	0.04	0.03
PDE1B	2612	0.05	0.04	0.04	0.03	0.03	0.03	0.03	0.03	0.04	0.04
IRAK3	5591	0.04	0.03	0.04	0.04	0.04	0.03	0.03	0.03	0.04	0.04
TJP2	2117	0.05	0.03	0.03	0.03	0.05	0.04	0.03	0.03	0.04	0.03
IRF7	1227	0.04	0.03	0.03	0.06	0.04	0.03	0.03	0.03	0.03	0.03
TJP1	5275	0.03	0.04	0.03	0.03	0.03	0.04	0.04	0.05	0.04	0.06
SHH	6007	0.03	0.06	0.05	0.03	0.03	0.03	0.04	0.03	0.04	0.06
ADCYAP1	41	0.02	0.03	0.04	0.03	0.05	0.03	0.03	0.04	0.04	0.06
CCNA1	186	0.05	0.04	0.03	0.03	0.04	0.03	0.03	0.03	0.04	0.05
EFNA1	1825	0.04	0.03	0.03	0.04	0.06	0.04	0.03	0.03	0.04	0.03
FN1	3293	0.03	0.03	0.03	0.02	0.04	0.04	0.04	0.04	0.04	0.06
EPHA5	2693	0.03	0.03	0.03	0.04	0.05	0.04	0.03	0.03	0.03	0.06
AREG	101	0.03	0.03	0.03	0.03	0.04	0.04	0.03	0.04	0.06	0.04
APP	3007	0.03	0.04	0.03	0.04	0.04	0.03	0.03	0.04	0.06	0.04
VEGFB	6107	0.04	0.02	0.02	0.04	0.04	0.03	0.04	0.03	0.04	0.06
NPY	1640	0.04	0.03	0.04	0.03	0.04	0.04	0.03	0.03	0.06	0.04
RET	6103	0.05	0.04	0.03	0.04	0.05	0.03	0.03	0.03	0.05	0.05
CDK2	2360	0.03	0.05	0.03	0.04	0.04	0.05	0.03	0.03	0.05	0.04
MAPK14	4072	0.06	0.02	0.04	0.03	0.04	0.03	0.03	0.07	0.03	0.07
ST6GAL1	2051	0.03	0.03	0.03	0.04	0.03	0.04	0.04	0.05	0.03	0.04
WT1	2966	0.05	0.03	0.03	0.03	0.04	0.04	0.06	0.03	0.05	0.09
TESK2	2361	0.06	0.03	0.04	0.04	0.04	0.03	0.03	0.02	0.02	0.02
TFAP2C	6048	0.03	0.04	0.04	0.03	0.03	0.04	0.04	0.03	0.06	0.08
ENC1	3517	0.04	0.03	0.03	0.03	0.04	0.04	0.04	0.04	0.05	0.04
BMPR2	1938	0.06	0.03	0.03	0.03	0.04	0.05	0.05	0.03	0.03	0.04
HBEGF	1955	0.03	0.07	0.05	0.03	0.04	0.05	0.03	0.03	0.04	0.04
EGFR	2434	0.03	0.03	0.04	0.04	0.04	0.04	0.03	0.04	0.05	0.06
TMEFF2	6130	0.04	0.04	0.02	0.04	0.03	0.04	0.04	0.02	0.05	0.1
HPSE	2252	0.03	0.04	0.04	0.04	0.04	0.07	0.04	0.03	0.03	0.02
ESR1	4092	0.03	0.04	0.03	0.03	0.04	0.07	0.03	0.04	0.05	0.07
IRAK3	4952	0.04	0.04	0.03	0.04	0.04	0.03	0.04	0.03	0.05	0.06
NQO1	974	0.03	0.04	0.03	0.04	0.04	0.03	0.04	0.04	0.05	0.04
SEZ6L	1935	0.03	0.03	0.04	0.03	0.03	0.05	0.04	0.04	0.03	0.04
PTGS1	4295	0.03	0.04	0.03	0.04	0.04	0.04	0.04	0.03	0.05	0.03
POMC	3805	0.04	0.03	0.05	0.05	0.04	0.04	0.03	0.03	0.04	0.04
WNT2B	5389	0.05	0.04	0.02	0.05	0.03	0.04	0.03	0.04	0.03	0.02
FANCG	1330	0.04	0.04	0.04	0.03	0.03	0.04	0.04	0.04	0.03	0.04
DST	4843	0.03	0.04	0.04	0.03	0.04	0.04	0.03	0.03	0.05	0.04
PSIP1	2795	0.03	0.03	0.02	0.04	0.04	0.04	0.04	0.05	0.02	0.06
HLF	678	0.03	0.03	0.04	0.05	0.04	0.04	0.04	0.04	0.03	0.06
SOX1	5941	0.04	0.06	0.02	0.03	0.04	0.05	0.05	0.02	0.02	0.08
CDKN1A	4066	0.04	0.04	0.04	0.03	0.04	0.04	0.03	0.04	0.03	0.05
CDKN1A	2370	0.07	0.03	0.04	0.05	0.04	0.04	0.03	0.04	0.03	0.03
ICA1	5884	0.03	0.04	0.04	0.03	0.04	0.05	0.03	0.05	0.04	0.06
ETS2	3927	0.05	0.04	0.04	0.03	0.04	0.04	0.04	0.04	0.03	0.04
FGF8	5683	0.04	0.02	0.03	0.07	0.04	0.04	0.03	0.03	0.03	0.04
NTRK2	4919	0.09	0.04	0.03	0.04	0.05	0.03	0.05	0.02	0.02	0.04
APP	80	0.04	0.03	0.04	0.04	0.04	0.04	0.04	0.03	0.04	0.06
PTCH	4157	0.04	0.04	0.04	0.03	0.04	0.04	0.04	0.04	0.03	0.03
MCAM	4262	0.03	0.03	0.04	0.05	0.04	0.04	0.04	0.03	0.06	0.08
IHH	4947	0.04	0.03	0.03	0.04	0.04	0.05	0.04	0.06	0.08	0.06
CDC25B	124	0.02	0.05	0.03	0.04	0.04	0.04	0.04	0.03	0.04	0.03
NRAS	4215	0.03	0.03	0.04	0.03	0.04	0.04	0.04	0.05	0.06	0.05
ITPR3	1255	0.03	0.04	0.04	0.04	0.04	0.04	0.04	0.03	0.04	0.04
FYN	4057	0.03	0.04	0.04	0.06	0.04	0.04	0.04	0.03	0.05	0.04
GAS7	606	0.06	0.04	0.03	0.04	0.05	0.04	0.04	0.04	0.04	0.05
EIF2AK2	5618	0.05	0.04	0.04	0.03	0.04	0.04	0.04	0.04	0.04	0.04
MST1R	4161	0.06	0.04	0.05	0.02	0.04	0.03	0.04	0.04	0.05	0.06
CTGF	1277	0.03	0.04	0.05	0.04	0.04	0.04	0.04	0.04	0.05	0.04

PGF	4965	0.057	0.087	0.043	0.050	0.039	0.056	0.046	0.031	0.187	0.483
SOX17	5943	0.048	0.047	0.048	0.047	0.054	0.048	0.049	0.048	0.101	0.235
PRKCDBP	1762	0.048	0.027	0.049	0.056	0.048	0.046	0.050	0.053	0.175	0.107
SEMA3C	2926	0.076	0.043	0.052	0.031	0.047	0.053	0.046	0.049	0.058	0.135
IL11	4188	0.040	0.047	0.043	0.079	0.050	0.062	0.104	0.047	0.080	0.081
ERCC1	3881	0.044	0.077	0.052	0.056	0.052	0.047	0.040	0.042	0.213	0.140
MT1A	3695	0.070	0.050	0.074	0.048	0.055	0.041	0.045	0.046	0.073	0.122
GFI1	607	0.036	0.059	0.068	0.035	0.046	0.054	0.055	0.040	0.093	0.126
CTNNB1	2415	0.047	0.052	0.036	0.066	0.066	0.044	0.065	0.044	0.084	0.077
TFRC	5251	0.058	0.063	0.043	0.041	0.047	0.061	0.046	0.053	0.078	0.079
ERBB3	4091	0.043	0.041	0.053	0.053	0.045	0.053	0.064	0.048	0.066	0.111
EDN1	3160	0.066	0.047	0.050	0.054	0.059	0.050	0.050	0.051	0.103	0.130
GRB10	942	0.075	0.071	0.029	0.049	0.051	0.040	0.078	0.051	0.165	0.268
FGF2	3726	0.054	0.067	0.032	0.071	0.044	0.056	0.049	0.034	0.102	0.236
SOX1	5935	0.057	0.034	0.041	0.110	0.049	0.055	0.069	0.046	0.085	0.176
TUBB3	2163	0.062	0.052	0.052	0.081	0.069	0.040	0.048	0.047	0.081	0.104
CDKN2B	6012	0.103	0.065	0.025	0.111	0.057	0.033	0.047	0.020	0.032	0.154
PALM2-AKAP2	5117	0.048	0.039	0.046	0.058	0.065	0.056	0.082	0.044	0.094	0.134
DLK1	3150	0.050	0.057	0.043	0.068	0.056	0.057	0.046	0.042	0.061	0.275
PXN	4826	0.092	0.057	0.051	0.042	0.062	0.055	0.039	0.042	0.143	0.198
TBX1	5961	0.061	0.053	0.053	0.067	0.062	0.050	0.054	0.049	0.074	0.102
HTR1B	3489	0.049	0.059	0.068	0.043	0.069	0.062	0.051	0.047	0.094	0.417
MYLK	5076	0.075	0.053	0.052	0.045	0.060	0.069	0.056	0.051	0.385	0.185
PTPN6	1776	0.100	0.059	0.051	0.036	0.079	0.025	0.061	0.035	0.097	0.502
CTSH	601	0.050	0.041	0.056	0.055	0.058	0.046	0.066	0.057	0.072	0.085
COPG2	361	0.054	0.060	0.057	0.057	0.062	0.051	0.051	0.053	0.122	0.123
CCND1	3085	0.047	0.055	0.059	0.072	0.064	0.058	0.048	0.054	0.094	0.067
RBP1	1841	0.057	0.055	0.078	0.044	0.046	0.063	0.045	0.061	0.103	0.212
DNMT3B	692	0.065	0.041	0.045	0.069	0.052	0.061	0.098	0.045	0.335	0.203
IGF2AS	1110	0.133	0.048	0.068	0.056	0.055	0.057	0.093	0.038	0.164	0.347
ZMYND10	2208	0.059	0.056	0.049	0.044	0.057	0.063	0.066	0.028	0.056	0.245
MCM2	2322	0.119	0.052	0.072	0.050	0.054	0.059	0.060	0.052	0.096	0.148
KRAS	1285	0.049	0.057	0.053	0.058	0.073	0.064	0.056	0.060	0.082	0.082
NRG1	5678	0.055	0.057	0.069	0.058	0.073	0.078	0.052	0.049	0.054	0.104
MT1A	1563	0.091	0.062	0.075	0.056	0.059	0.048	0.049	0.054	0.083	0.218
CRIP1	403	0.093	0.055	0.063	0.100	0.061	0.051	0.047	0.039	0.141	0.215
NGFR	4197	0.065	0.052	0.056	0.060	0.068	0.056	0.059	0.057	0.102	0.091
PYCARD	1799	0.096	0.044	0.060	0.075	0.058	0.059	0.058	0.066	0.156	0.235
CDKN2A	4068	0.052	0.062	0.063	0.050	0.069	0.053	0.063	0.057	0.080	0.087
ETV1	3941	0.089	0.051	0.060	0.056	0.080	0.077	0.062	0.059	0.083	0.161
CHGA	3075	0.097	0.057	0.057	0.078	0.064	0.067	0.056	0.055	0.051	0.194
TNFRSF1B	1331	0.084	0.060	0.049	0.086	0.052	0.062	0.055	0.065	0.093	0.105
WNT2	1377	0.063	0.066	0.060	0.044	0.057	0.078	0.057	0.067	0.067	0.098
TP73	2932	0.058	0.053	0.070	0.061	0.055	0.066	0.062	0.065	0.079	0.179
TWIST1	2167	0.082	0.092	0.050	0.043	0.071	0.092	0.052	0.049	0.078	0.103
FABP3	778	0.085	0.055	0.056	0.062	0.069	0.061	0.067	0.059	0.093	0.152
HIC1	3398	0.051	0.054	0.079	0.063	0.053	0.062	0.061	0.076	0.114	0.048
DDB2	3632	0.083	0.063	0.061	0.076	0.061	0.064	0.057	0.045	0.090	0.215
ZMYND10	4012	0.111	0.075	0.062	0.139	0.044	0.046	0.062	0.060	0.115	0.094
EYA4	759	0.060	0.062	0.062	0.081	0.083	0.079	0.058	0.056	0.069	0.177
GSTM2	3354	0.061	0.053	0.043	0.114	0.070	0.050	0.073	0.064	0.057	0.138
PTGS2	2829	0.060	0.062	0.070	0.059	0.067	0.070	0.065	0.059	0.058	0.101
COL18A1	3346	0.039	0.046	0.049	0.084	0.073	0.069	0.058	0.076	0.071	0.129
SFRP1	3910	0.066	0.070	0.072	0.058	0.060	0.072	0.063	0.054	0.071	0.131
ITPR2	1248	0.090	0.059	0.053	0.068	0.069	0.065	0.064	0.052	0.077	0.150
TMEFF2	3980	0.089	0.085	0.065	0.058	0.081	0.065	0.062	0.062	0.095	0.174
GJB2	897	0.069	0.079	0.050	0.111	0.061	0.054	0.081	0.048	0.139	0.083
TNFSF8	1341	0.118	0.063	0.058	0.074	0.068	0.051	0.066	0.065	0.359	0.048
APBA1	2994	0.065	0.067	0.073	0.063	0.066	0.062	0.069	0.058	0.118	0.135
IGF2	4106	0.070	0.052	0.052	0.078	0.066	0.033	0.093	0.066	0.072	0.179
MMP2	925	0.053	0.065	0.044	0.136	0.075	0.068	0.088	0.053	0.088	0.089
CLK1	4290	0.100	0.071	0.069	0.054	0.062	0.064	0.081	0.059	0.207	0.237
KDR	800	0.067	0.054	0.073	0.062	0.076	0.076	0.067	0.061	0.088	0.098
TUSC3	3992	0.049	0.069	0.048	0.039	0.065	0.074	0.076	0.069	0.057	0.221
IFNGR2	4125	0.068	0.070	0.055	0.056	0.086	0.066	0.057	0.071	0.252	0.334
TMEFF2	2139	0.058	0.109	0.058	0.083	0.047	0.034	0.076	0.125	0.057	0.121
JUNB	4230	0.064	0.071	0.072	0.084	0.072	0.063	0.064	0.059	0.080	0.091
ST6GAL1	2050	0.045	0.051	0.096	0.099	0.089	0.064	0.072	0.045	0.128	0.074
INSR	1994	0.050	0.073	0.043	0.048	0.069	0.077	0.067	0.078	0.068	0.271
TUSC3	2164	0.080	0.068	0.068	0.070	0.068	0.061	0.089	0.062	0.097	0.151
GFI1	4071	0.086	0.047	0.070	0.059	0.069	0.092	0.068	0.033	0.187	0.230
HHIP	2209	0.100	0.065	0.061	0.049	0.072	0.085	0.086	0.058	0.110	0.104
EPHA7	1850	0.046	0.075	0.051	0.051	0.071	0.076	0.071	0.067	0.066	0.107
SH3BP2	4982	0.074	0.079	0.066	0.073	0.062	0.048	0.073	0.063	0.101	0.100
NOTCH3	5672	0.077	0.057	0.067	0.082	0.072	0.060	0.074	0.056	0.055	0.159
LAMC1	5022	0.069	0.088	0.068	0.075	0.065	0.095	0.071	0.060	0.057	0.132
MCC	887	0.093	0.053	0.071	0.061	0.070	0.078	0.068	0.080	0.072	0.096
RAD54B	5965	0.099	0.065	0.077	0.050	0.086	0.059	0.071	0.071	0.062	0.187
EPHA3	744	0.146	0.084	0.049	0.102	0.070	0.061	0.072	0.044	0.052	0.205
CSPG2	2409	0.123	0.069	0.068	0.053	0.073	0.069	0.119	0.119	0.102	0.177
BDNF	2840	0.067	0.075	0.076	0.069	0.073	0.081	0.065	0.069	0.092	0.105
DSC2	2630	0.070	0.067	0.073	0.060	0.079	0.086	0.075	0.056	0.118	0.391
HLA-DRA	1046	0.070	0.066	0.072	0.072	0.071	0.087	0.077	0.075	0.084	0.081
TNFRSF10D	2149	0.072	0.074	0.079	0.068	0.070	0.085	0.072	0.073	0.078	0.080
NR2F6	2843	0.083	0.066	0.066	0.081	0.077	0.069	0.078	0.064	0.090	0.129
CDH11	172	0.068	0.069	0.076	0.069	0.088	0.104	0.077	0.070	0.080	0.129
CAV1	2327	0.078	0.087	0.059	0.072	0.074	0.076	0.056	0.054	0.153	0.174
HS3ST2	1088	0.057	0.069	0.078	0.094	0.064	0.078	0.086	0.066	0.071	0.168
EPM2A	747	0.057	0.082	0.077	0.067	0.073	0.085	0.074	0.067	0.087	0.100
GADD45A	5733	0.066	0.080	0.071	0.080	0.064	0.083	0.076	0.059	0.114	0.153
SGCE	3922	0.090	0.076	0.050	0.089	0.069	0.080	0.071	0.060	0.116	0.120
IGF2	2680	0.163	0.076	0.052	0.109	0.088	0.058	0.072	0.056	0.141	0.200
DDR1	5511	0.079	0.069	0.070	0.106	0.078	0.088	0.063	0.063	0.090	0.134
TMEM63A	2943	0.063	0.088	0.076	0.073	0.073	0.075	0.071	0.084	0.165	0.064
ICA1	5885	0.076	0.069	0.084	0.065	0.075	0.074	0.059	0.087	0.080	0.114
TDGF1	5056	0.130	0.129	0.048	0.077	0.073	0.067	0.081	0.049	0.232	0.281
PDE1B	2862	0.050	0.080	0.083	0.055	0.055	0.073	0.080	0.082	0.093	0.099
ETV1	3670	0.077	0.087	0.074	0.060	0.080	0.093	0.076	0.070	0.106	0.175
CTTN	1286	0.096	0.091	0.062	0.114	0.081	0.071	0.064	0.072	0.086	0.095
GAS7	4060	0.097	0.080	0.088	0.071	0.080	0.058	0.050	0.074	0.092	0.103
RARA	1151	0.088	0.086	0.095	0.067	0.071	0.076	0.079	0.073	0.125	0.228
BDNF	1862	0.075	0.080	0.102	0.070	0.076	0.072	0.084	0.079	0.162	0.293
NEFL	3711	0.086	0.078	0.083	0.076	0.071	0.077	0.083	0.067	0.100	0.160
UBA52	2404	0.109	0.073	0.066	0.151	0.083	0.061	0.112	0.056	0.115	0.105
PHLDA2	3777	0.071	0.086	0.077	0.092	0.070	0.080	0.084	0.068	0.130	0.058
ISL1	5600	0.095	0.077	0.072	0.046	0.080	0.086	0.097	0.064	0.062	0.103
ID1	2271	0.087	0.071	0.080	0.082	0.093	0.073	0.077	0.060	0.076	0.165
EYA4	758	0.076	0.090	0.068	0.102	0.082	0.072	0.087	0.049	0.082	0.095
ESR1	6020	0.068	0.089	0.084	0.073	0.082	0.076	0.116	0.069	0.077	0.136
ZNF264	4017	0.071	0.079	0.075	0.080	0.093	0.091	0.068	0.091	0.110	0.067
EPHB4	5550	0.087	0.081	0.069	0.085	0.105	0.079	0.069	0.068	0.079	0.084
HIC2	1002	0.078	0.063	0.061	0.093	0.085	0.085	0.082	0.066	0.083	0.100
CSK	3479	0.140	0.061	0.056	0.068	0.060	0.096	0.108	0.093	0.104	0.060
WNT1	6115	0.084	0.078	0.080	0.082	0.086	0.090	0.078	0.077	0.107	0.181
CEBPA	3294	0.099	0.088	0.080	0.135	0.076	0.080	0.070	0.082	0.096	0.084
ACVR1B	5801	0.090	0.078	0.558	0.070	0.072	0.086	0.068	0.654	0.082	0.120
FGF12	437	0.073	0.078	0.100	0.081	0.088	0.092	0.084	0.071	0.129	0.152
WNT2	5252	0.093	0.084	0.083	0.097	0.077	0.081	0.082	0.070	0.115	0.168
APC	2257	0.096	0.068	0.098	0.094	0.072	0.088	0.075	0.078	0.129	0.162
FGF2	4008	0.191	0.078	0.101	0.088	0.086	0.082	0.077	0.074	0.329	0.368
HCK	5768	0.141	0.089	0.077	0.149	0.080	0.067	0.109	0.062	0.069	0.140
IGFBP2	5710	0.114	0.062	0.103	0.082	0.095	0.076	0.062	0.087	0.126	0.196
TCF4	5250	0.085	0.068	0.100	0.057	0.086	0.087	0.074	0.094	0.074	0.116
ABO	30	0.125	0.091	0.080	0.104	0.099	0.072	0.080	0.076	0.192	0.095
TNFRSF10B	5361	0.086	0.090	0.077	0.074	0.085	0.072	0.135	0.087	0.079	0.078
RARB	2853	0.087	0.153	0.068	0.043	0.103	0.095	0.084	0.075	0.098	0.101
TGFBI	5310	0.111	0.098	0.111	0.081	0.076	0.085	0.087	0.078	0.078	0.139
RIPK4	2311	0.071	0.054	0.098	0.092	0.080	0.113	0.119	0.063	0.172	0.136
FASTK	4872	0.098	0.070	0.089	0.129	0.091	0.078	0.084	0.065	0.117	0.119
RIPK2	2902	0.099	0.091	0.078	0.079	0.087	0.087	0.097	0.057	0.117	0.096
MTA1	5764	0.085	0.089	0.066	0.139	0.099	0.077	0.096	0.086	0.086	0.081
NGFB	3030	0.078	0.104	0.086	0.060	0.085	0.110	0.109	0.089	0.058	0.122
PTGS1	1051	0.090	0.092	0.067	0.106	0.075	0.085	0.093	0.073	0.059	0.116
TNC	5290	0.066	0.081	0.105	0.140	0.094	0.069	0.145	0.079	0.195	0.160
DNMT1	671	0.091	0.081	0.096	0.095	0.090	0.086	0.066	0.072	0.101	0.081
HOXA9	631	0.096	0.114	0.087	0.064	0.086	0.129	0.064	0.090	0.116	0.141
MALT1	4065	0.111	0.095	0.080	0.067	0.113	0.115	0.081	0.067	0.079	0.120

AATK	3	0.32	0.2	0.13	0.12	0.12	0.15	0.1	0.08	0.95	0.94
CREBBP	3290	0.37	0.14	0.17	0.17	0.12	0.11	0.09	0.09	0.76	0.83
IL12B	3938	0.28	0.18	0.17	0.09	0.12	0.14	0.13	0.12	0.83	0.81
ASCL1	102	0.16	0.15	0.14	0.14	0.11	0.13	0.11	0.1	0.63	0.25
DLC1	2430	0.24	0.15	0.17	0.1	0.14	0.06	0.09	0.14	0.87	0.58
HLA-DOA	1006	0.38	0.14	0.12	0.09	0.15	0.15	0.13	0.13	0.79	0.78
SYK	2883	0.23	0.13	0.08	0.18	0.15	0.15	0.13	0.12	0.3	0.46
FRK	1913	0.34	0.13	0.15	0.19	0.09	0.09	0.05	0.15	0.66	0.84
BCR	2298	0.15	0.13	0.12	0.17	0.13	0.23	0.09	0.14	0.63	0.66
DNMT2	682	0.41	0.26	0.22	0.08	0.13	0.15	0.12	0.11	0.92	0.88
MSH2	2768	0.27	0.14	0.14	0.15	0.14	0.17	0.14	0.11	0.89	0.84
KCNK4	1262	0.15	0.16	0.14	0.12	0.15	0.14	0.13	0.11	0.32	0.55
EPHA8	1856	0.36	0.2	0.21	0.14	0.12	0.1	0.14	0.14	0.82	0.84
XRCC2	5449	0.49	0.21	0.18	0.11	0.13	0.13	0.16	0.1	0.98	0.97
NPR2	2418	0.46	0.26	0.2	0.15	0.11	0.1	0.15	0.09	0.79	0.9
UGT1A1	2820	0.51	0.22	0.21	0.13	0.15	0.11	0.16	0.12	0.95	0.95
USP29	2184	0.33	0.17	0.14	0.23	0.14	0.13	0.18	0.11	0.9	0.81
NDN	3709	0.29	0.18	0.18	0.14	0.16	0.16	0.14	0.1	0.78	0.81
TFDP1	6041	0.22	0.19	0.16	0.07	0.16	0.15	0.14	0.17	0.77	0.64
PLAGL1	1693	0.32	0.18	0.19	0.22	0.15	0.11	0.14	0.08	0.81	0.84
EPHA5	2095	0.21	0.16	0.17	0.18	0.12	0.18	0.16	0.13	0.38	0.54
CTGF	5560	0.41	0.23	0.23	0.17	0.14	0.09	0.17	0.16	0.96	0.95
AATK	10	0.22	0.16	0.21	0.23	0.18	0.16	0.17	0.14	0.91	0.86
RARA	5097	0.23	0.16	0.18	0.19	0.2	0.13	0.18	0.09	0.24	0.66
NCL	1587	0.46	0.22	0.22	0.15	0.17	0.19	0.18	0.16	0.95	0.96
MSH3	2787	0.39	0.11	0.15	0.2	0.17	0.2	0.2	0.27	0.6	0.76
SFN	3909	0.62	0.21	0.23	0.21	0.08	0.19	0.12	0.13	0.94	0.93
FGFR4	4036	0.48	0.17	0.24	0.21	0.2	0.24	0.19	0.12	0.91	0.89
PARP1	5915	0.39	0.21	0.17	0.22	0.19	0.2	0.24	0.13	0.85	0.89
MKRN3	3676	0.4	0.56	0.25	0.08	0.18	0.05	0.32	0.11	0.95	0.95
USP29	4004	0.5	0.22	0.26	0.25	0.19	0.21	0.23	0.14	0.97	0.95
MMP14	4149	0.25	0.28	0.26	0.22	0.24	0.19	0.2	0.22	0.38	0.46
HPN	4931	0.61	0.13	0.36	0.24	0.1	0.33	0.25	0.2	0.42	0.58
PGR	4154	0.41	0.26	0.27	0.29	0.22	0.23	0.2	0.23	0.81	0.75
USP29	2185	0.69	0.38	0.26	0.29	0.23	0.16	0.26	0.2	0.93	0.94
CDH11	3284	0.21	0.26	0.26	0.29	0.27	0.28	0.22	0.22	0.24	0.52
IL12B	4190	0.49	0.32	0.27	0.22	0.29	0.25	0.25	0.21	0.88	0.88
PLSCR3	2705	0.29	0.27	0.31	0.3	0.33	0.29	0.28	0.26	0.42	0.34
TDG	2934	0.62	0.32	0.27	0.37	0.33	0.17	0.46	0.16	0.87	0.84
SNRPN	2029	0.58	0.43	0.27	0.26	0.33	0.21	0.34	0.35	0.89	0.91
MKRN3	1439	0.44	0.71	0.45	0.09	0.25	0.15	0.5	0.24	0.92	0.93
MSH3	4137	0.63	0.26	0.33	0.46	0.29	0.36	0.32	0.44	0.8	0.88
UGT1A1	2962	0.52	0.36	0.34	0.36	0.34	0.11	0.47	0.28	0.97	0.96
HLA-DOB	3429	0.49	0.32	0.45	0.35	0.31	0.24	0.39	0.36	0.78	0.78
HLA-DPB1	1030	0.66	0.38	0.44	0.33	0.26	0.3	0.5	0.34	0.96	0.94
SLC22A2	1983	0.52	0.25	0.39	0.48	0.28	0.36	0.49	0.19	0.88	0.87
MXI1	4166	0.4	0.71	0.25	0.32	0.69	0.28	0.75	0.35	0.32	0.64
DHCR24	1780	0.36	0.4	0.42	0.44	0.39	0.4	0.39	0.33	0.49	0.54
NTRK1	989	0.96	0.95	0.38	0.34	0.39	0.4	0.36	0.96	0.46	0.37
TBX1	5962	0.42	0.38	0.41	0.49	0.38	0.42	0.38	0.39	0.71	0.77
IGSF4C	3500	0.6	0.32	0.45	0.51	0.4	0.38	0.41	0.31	0.31	0.56
EFNB3	2898	0.4	0.43	0.47	0.47	0.41	0.4	0.38	0.41	0.48	0.42
FRK	2195	0.62	0.42	0.49	0.46	0.41	0.22	0.37	0.41	0.86	0.81
WEE1	2828	0.57	0.49	0.4	0.46	0.38	0.31	0.35	0.49	0.96	0.93
ZP3	2232	0.6	0.53	0.45	0.4	0.42	0.61	0.46	0.38	0.54	0.83
PYCARD	1795	0.51	0.48	0.46	0.58	0.45	0.43	0.48	0.43	0.49	0.58
HLA-DOA	1009	0.59	0.59	0.55	0.41	0.46	0.48	0.46	0.46	0.29	0.56
FGF6	4016	0.66	0.5	0.55	0.57	0.16	0.29	0.37	0.49	0.93	0.93
RARRES1	1823	0.57	0.47	0.48	0.54	0.52	0.49	0.52	0.43	0.44	0.54
FASTK	4874	0.57	0.58	0.55	0.5	0.49	0.51	0.5	0.5	0.86	0.83
PDGFB	5934	0.63	0.53	0.67	0.58	0.46	0.32	0.35	0.48	0.58	0.72
CRIP1	427	0.5	0.54	0.51	0.58	0.53	0.52	0.51	0.41	0.43	0.57
MOS	2749	0.63	0.53	0.61	0.43	0.51	0.39	0.47	0.53	0.91	0.79
TDGF1	1250	0.55	0.54	0.39	0.78	0.5	0.5	0.63	0.32	0.85	0.85
CDKN2A	6139	0.54	0.5	0.57	0.59	0.53	0.55	0.53	0.46	0.47	0.64
IL1B	3958	0.46	0.58	0.58	0.56	0.57	0.55	0.55	0.6	0.86	0.56
HLA-DOB	1011	0.65	0.58	0.65	0.54	0.5	0.38	0.56	0.6	0.93	0.92
EPHB1	2903	0.71	0.6	0.56	0.65	0.56	0.58	0.57	0.47	0.63	0.72
HBII-52	961	0.79	0.6	0.25	0.68	0.33	0.61	0.71	0.59	0.9	0.87
SLC5A5	3929	0.75	0.58	0.66	0.46	0.65	0.52	0.42	0.73	0.34	0.76
HBII-52	963	0.83	0.6	0.23	0.68	0.33	0.59	0.76	0.65	0.91	0.88
IFNG	1629	0.63	0.61	0.6	0.52	0.64	0.55	0.67	0.64	0.85	0.53
NDN	1595	0.7	0.63	0.62	0.55	0.62	0.41	0.66	0.54	0.86	0.9
TGFB1	2895	0.71	0.61	0.76	0.47	0.67	0.27	0.33	0.64	0.95	0.96
HBII-52	3375	0.71	0.78	0.61	0.47	0.64	0.53	0.63	0.74	0.76	0.54
FGF6	460	0.8	0.66	0.66	0.65	0.32	0.43	0.44	0.64	0.92	0.94
MUC1	1676	0.66	0.69	0.7	0.63	0.58	0.47	0.64	0.72	0.41	0.5
ARHGAP9	1842	0.67	0.68	0.62	0.8	0.64	0.52	0.69	0.63	0.94	0.92
KLK11	5003	0.66	0.69	0.59	0.68	0.64	0.64	0.69	0.66	0.92	0.9
PTK7	1077	0.66	0.71	0.6	0.73	0.63	0.67	0.7	0.66	0.51	0.51
BAX	3031	0.71	0.7	0.68	0.74	0.67	0.67	0.68	0.71	0.63	0.68
MC2R	1360	0.71	0.63	0.56	0.81	0.74	0.69	0.64	0.75	0.8	0.73
LMTK2	2316	0.75	0.7	0.69	0.73	0.66	0.7	0.73	0.68	0.76	0.62
ERCC3	3901	0.74	0.76	0.69	0.63	0.79	0.54	0.71	0.64	0.55	0.74
MMP7	931	0.67	0.74	0.69	0.67	0.72	0.71	0.76	0.71	0.48	0.28
IL16	1173	0.72	0.7	0.73	0.73	0.65	0.66	0.71	0.76	0.27	0.55
HSPA2	1098	0.74	0.71	0.73	0.7	0.7	0.7	0.73	0.72	0.95	0.93
CSF1R	3420	0.76	0.73	0.73	0.64	0.71	0.67	0.69	0.77	0.62	0.65
LRRC32	3027	0.81	0.78	0.78	0.7	0.71	0.61	0.69	0.73	0.94	0.93
TEK	5057	0.74	0.72	0.73	0.67	0.62	0.75	0.72	0.61	0.74	0.85
MUSK	2390	0.77	0.73	0.7	0.72	0.73	0.75	0.77	0.72	0.86	0.78
TEK	5058	0.72	0.77	0.77	0.72	0.7	0.76	0.79	0.7	0.69	0.82
SPDEF	3942	0.69	0.76	0.77	0.78	0.75	0.74	0.74	0.71	0.76	0.54
HLA-DPA1	3435	0.86	0.7	0.61	0.73	0.76	0.63	0.84	0.8	0.36	0.39
EPHX1	1882	0.74	0.76	0.76	0.75	0.76	0.7	0.76	0.75	0.76	0.58
FOSL2	585	0.87	0.85	0.77	0.77	0.73	0.76	0.71	0.69	0.9	0.96
PRSS1	2247	0.78	0.76	0.72	0.8	0.76	0.73	0.77	0.77	0.81	0.72
DES	4312	0.84	0.8	0.69	0.7	0.79	0.7	0.74	0.79	0.82	0.81
LRRK1	2091	0.76	0.79	0.77	0.81	0.78	0.76	0.8	0.76	0.42	0.56
AOC3	4223	0.78	0.8	0.82	0.75	0.77	0.74	0.78	0.81	0.7	0.53
C4B	3042	0.78	0.8	0.72	0.64	0.79	0.8	0.73	0.8	0.78	0.74
MAPK10	884	0.89	0.68	0.82	0.74	0.75	0.72	0.87	0.87	0.93	0.9
PTK6	5700	0.74	0.79	0.8	0.69	0.74	0.78	0.79	0.87	0.63	0.45
GABRA5	3295	0.8	0.82	0.77	0.8	0.78	0.8	0.75	0.77	0.84	0.76
PGR	2813	0.82	0.79	0.82	0.79	0.79	0.78	0.78	0.82	0.81	0.68
GNG7	1944	0.82	0.83	0.79	0.7	0.78	0.8	0.75	0.86	0.9	0.88
SEPT9	6002	0.87	0.8	0.9	0.79	0.63	0.88	0.4	0.79	0.88	0.89
HLA-DQA2	3450	0.82	0.8	0.78	0.79	0.82	0.75	0.79	0.8	0.83	0.65
CCR5	1761	0.81	0.81	0.8	0.82	0.77	0.78	0.81	0.78	0.68	0.65
SERPINA5	3896	0.82	0.82	0.78	0.82	0.81	0.78	0.81	0.79	0.78	0.81
S100A12	1871	0.81	0.83	0.79	0.81	0.82	0.8	0.81	0.83	0.83	0.83
IL12B	731	0.88	0.83	0.85	0.78	0.8	0.71	0.79	0.82	0.85	0.89
MPL	5754	0.8	0.82	0.83	0.83	0.81	0.8	0.83	0.79	0.73	0.38
HLA-DQA2	1039	0.73	0.82	0.83	0.79	0.82	0.76	0.81	0.82	0.85	0.77
CTLA4	594	0.86	0.82	0.83	0.89	0.8	0.81	0.82	0.81	0.91	0.85
FLJ20712	807	0.84	0.84	0.81	0.86	0.8	0.8	0.82	0.83	0.87	0.74
SRC	1746	0.83	0.85	0.81	0.82	0.84	0.79	0.81	0.85	0.91	0.86
PLA2G2A	1040	0.79	0.82	0.83	0.84	0.8	0.83	0.79	0.85	0.83	0.78
ARHGAP9	1787	0.84	0.83	0.81	0.87	0.84	0.74	0.82	0.81	0.94	0.92
APOC1	69	0.85	0.83	0.85	0.82	0.83	0.79	0.84	0.82	0.78	0.8
CHD2	263	0.89	0.86	0.83	0.83	0.83	0.8	0.85	0.81	0.94	0.86
IL4	3975	0.85	0.83	0.84	0.86	0.82	0.81	0.84	0.83	0.82	0.76
PGR	2811	0.8	0.85	0.83	0.84	0.82	0.76	0.84	0.85	0.93	0.92
APOC2	74	0.86	0.87	0.84	0.81	0.82	0.83	0.85	0.87	0.8	0.79
PLG	4268	0.86	0.84	0.84	0.8	0.86	0.81	0.84	0.85	0.89	0.89
GFAP	5877	0.83	0.82	0.85	0.86	0.85	0.85	0.85	0.87	0.92	0.82
CPA4	3087	0.84	0.87	0.86	0.84	0.85	0.83	0.87	0.85	0.85	0.75
SLC14A1	5188	0.88	0.84	0.86	0.88	0.86	0.85	0.85	0.84	0.87	0.87
PSCA	5131	0.84	0.85	0.86	0.86	0.86	0.85	0.85	0.86	0.88	0.84
MMP7	4156	0.89	0.87	0.85	0.9	0.86	0.86	0.85	0.85	0.87	0.8
B3GALT5	2833	0.88	0.88	0.88	0.87	0.85	0.85	0.85	0.84	0.88	0.77
SERPINA5	1893	0.83	0.86	0.87	0.88	0.84	0.83	0.87	0.88	0.42	0.37
ASB4	96	0.89	0.85	0.87	0.88	0.83	0.83	0.85	0.87	0.93	0.9
GABRG3	864	0.89	0.88	0.86	0.88	0.86	0.84	0.85	0.86	0.86	0.79
SFTPC	3919	0.87	0.86	0.84	0.87	0.86	0.86	0.87	0.88	0.7	0.8
JAK3	4989	0.83	0.89	0.89	0.84	0.88	0.82	0.85	0.89	0.68	0.72
DSG1	2054	0.92	0.87	0.89	0.84	0.86	0.83	0.9	0.81	0.89	0.8

RARRES1	3858	0.03	0.04	0.04	0.06	0.04	0.04	0.04	0.03	0.04	0.04
TNFRSF10D	3985	0.04	0.03	0.03	0.04	0.05	0.04	0.06	0.04	0.04	0.05
SMARCA3	3933	0.05	0.04	0.04	0.04	0.04	0.04	0.04	0.04	0.05	0.05
TGFA	2893	0.06	0.04	0.04	0.03	0.05	0.04	0.04	0.04	0.05	0.07
FLT1	515	0.06	0.04	0.04	0.05	0.04	0.05	0.03	0.03	0.04	0.05
MCM6	2818	0.04	0.04	0.03	0.03	0.05	0.05	0.04	0.04	0.04	0.06
CD9	5854	0.04	0.04	0.04	0.07	0.05	0.04	0.04	0.04	0.05	0.08
FGF8	1334	0.04	0.04	0.03	0.04	0.05	0.04	0.04	0.04	0.05	0.06
TAL1	1055	0.05	0.04	0.04	0.05	0.05	0.03	0.04	0.03	0.06	0.05
FLT3	542	0.04	0.05	0.04	0.04	0.05	0.05	0.04	0.04	0.04	0.06
MYCN	4150	0.04	0.03	0.04	0.05	0.04	0.04	0.05	0.03	0.05	0.09
BCL6	2296	0.08	0.04	0.03	0.05	0.05	0.04	0.03	0.03	0.05	0.02
ERCC1	3893	0.04	0.04	0.04	0.04	0.04	0.04	0.04	0.04	0.03	0.04
ITGB4	781	0.05	0.03	0.05	0.03	0.05	0.03	0.05	0.04	0.05	0.04
GSTP1	6038	0.04	0.04	0.04	0.04	0.05	0.04	0.04	0.04	0.05	0.04
SLC22A3	3926	0.03	0.03	0.04	0.04	0.04	0.05	0.06	0.04	0.03	0.11
FZD9	4880	0.03	0.08	0.03	0.04	0.04	0.06	0.03	0.04	0.05	0.03
MYH11	2152	0.03	0.03	0.04	0.05	0.06	0.03	0.04	0.16	0.05	0.07
MYH11	2403	0.02	0.04	0.02	0.05	0.04	0.03	0.04	0.05	0.04	0.05
PTK2B	2254	0.04	0.04	0.05	0.04	0.04	0.04	0.04	0.04	0.05	0.04
HOXA11	1063	0.05	0.03	0.04	0.08	0.05	0.04	0.03	0.04	0.04	0.04
GP1BB	926	0.05	0.03	0.04	0.04	0.04	0.04	0.03	0.04	0.04	0.1
EPHB1	1861	0.04	0.04	0.03	0.04	0.03	0.05	0.05	0.03	0.07	0.07
ESR2	3262	0.07	0.04	0.05	0.03	0.04	0.04	0.04	0.03	0.03	0.08
SMARCA3	2021	0.07	0.04	0.04	0.03	0.07	0.04	0.03	0.06	0.07	0.08
ETS1	3923	0.04	0.04	0.05	0.04	0.04	0.05	0.04	0.04	0.05	0.06
PTPRG	1099	0.05	0.03	0.03	0.06	0.04	0.04	0.05	0.03	0.06	0.07
FANCE	4848	0.04	0.04	0.04	0.05	0.05	0.04	0.04	0.04	0.06	0.06
RAF1	5069	0.05	0.04	0.04	0.04	0.04	0.04	0.04	0.04	0.04	0.09
NFKB2	4186	0.05	0.03	0.05	0.06	0.04	0.03	0.04	0.03	0.04	0.04
EPM2A	751	0.04	0.04	0.04	0.04	0.05	0.05	0.04	0.04	0.05	0.05
GAS1	5626	0.06	0.02	0.02	0.06	0.04	0.04	0.05	0.03	0.04	0.02
CDKN1C	240	0.03	0.06	0.03	0.07	0.05	0.04	0.04	0.03	0.06	0.08
TSG101	6082	0.04	0.04	0.05	0.03	0.06	0.04	0.07	0.04	0.06	0.07
PDGFRA	4234	0.04	0.04	0.05	0.03	0.06	0.04	0.04	0.05	0.05	0.09
EPHA7	2901	0.04	0.04	0.04	0.04	0.04	0.04	0.04	0.05	0.05	0.08
PROK2	3826	0.04	0.05	0.04	0.03	0.04	0.05	0.04	0.04	0.05	0.05
ALK	31	0.04	0.04	0.05	0.04	0.04	0.04	0.04	0.04	0.06	0.06
MMP2	4151	0.04	0.04	0.05	0.04	0.06	0.05	0.04	0.04	0.05	0.07
UNG	2180	0.05	0.02	0.03	0.04	0.06	0.04	0.04	0.05	0.03	0.04
COL1A2	3083	0.04	0.05	0.04	0.03	0.05	0.05	0.04	0.04	0.05	0.09
FLI1	5708	0.03	0.15	0.04	0.03	0.05	0.06	0.04	0.06	0.04	0.04
MAP3K9	5653	0.04	0.04	0.06	0.05	0.03	0.05	0.05	0.03	0.07	0.04
NTSR1	2450	0.04	0.04	0.04	0.06	0.04	0.04	0.04	0.05	0.06	0.07
GALR1	889	0.04	0.05	0.04	0.04	0.05	0.04	0.05	0.05	0.06	0.06
DAB2IP	614	0.04	0.04	0.05	0.06	0.04	0.05	0.05	0.04	0.07	0.04
LRP2	3574	0.05	0.03	0.04	0.04	0.05	0.05	0.03	0.05	0.05	0.06
EPHA1	265	0.03	0.03	0.05	0.08	0.05	0.05	0.04	0.04	0.04	0.04
ERGIC3	1740	0.04	0.04	0.05	0.04	0.05	0.05	0.04	0.05	0.06	0.07
ADCYAP1	2990	0.04	0.04	0.04	0.04	0.05	0.05	0.05	0.05	0.05	0.06
HOXB13	3478	0.07	0.07	0.05	0.04	0.04	0.04	0.05	0.02	0.04	0.09
TERT	4180	0.04	0.04	0.04	0.04	0.05	0.05	0.05	0.05	0.05	0.04
HRASLS	3481	0.04	0.05	0.06	0.03	0.05	0.06	0.05	0.04	0.04	0.05
ROR2	2324	0.04	0.04	0.05	0.05	0.06	0.05	0.04	0.04	0.06	0.03
CCND3	3091	0.06	0.05	0.03	0.06	0.05	0.05	0.04	0.04	0.06	0.05
GJB2	3318	0.03	0.05	0.06	0.03	0.05	0.04	0.06	0.04	0.06	0.08
WNT1	1757	0.06	0.05	0.05	0.05	0.04	0.04	0.04	0.04	0.05	0.07
CASP6	4260	0.05	0.04	0.04	0.06	0.05	0.06	0.04	0.04	0.08	0.08
JAK2	4228	0.04	0.05	0.05	0.04	0.05	0.06	0.04	0.05	0.07	0.05
FAS	4863	0.03	0.05	0.05	0.03	0.04	0.07	0.04	0.05	0.05	0.04
ACVR1C	5816	0.06	0.05	0.05	0.03	0.04	0.03	0.04	0.05	0.05	0.04
TNFSF8	5184	0.05	0.08	0.03	0.05	0.05	0.03	0.03	0.05	0.03	0.02
ACVR1C	5815	0.06	0.05	0.04	0.04	0.05	0.05	0.05	0.04	0.05	0.07
MAP3K1	5052	0.04	0.05	0.06	0.04	0.04	0.04	0.05	0.05	0.05	0.1
HPSE	1984	0.08	0.06	0.05	0.04	0.03	0.05	0.06	0.04	0.02	0.04
SEMA3F	5157	0.06	0.04	0.11	0.06	0.05	0.03	0.04	0.04	0.04	0.03
CDK6	3157	0.05	0.06	0.05	0.04	0.05	0.05	0.05	0.04	0.07	0.05
PAX6	3741	0.05	0.04	0.05	0.06	0.05	0.05	0.04	0.03	0.04	0.06
ROR1	2320	0.04	0.05	0.05	0.05	0.05	0.05	0.04	0.05	0.05	0.05
EPHB2	2127	0.04	0.04	0.1	0.07	0.05	0.04	0.06	0.05	0.06	0.07
THBS1	3972	0.05	0.05	0.05	0.05	0.05	0.05	0.04	0.04	0.06	0.06
FLT4	3804	0.06	0.04	0.05	0.06	0.06	0.04	0.04	0.03	0.04	0.06
HIF1A	4102	0.05	0.06	0.05	0.03	0.04	0.04	0.04	0.06	0.06	0.04
JAK3	4990	0.05	0.05	0.05	0.04	0.04	0.04	0.05	0.05	0.05	0.04
JAG2	5607	0.04	0.06	0.03	0.09	0.05	0.04	0.05	0.03	0.04	0.03
TCF4	5246	0.04	0.05	0.04	0.04	0.05	0.08	0.06	0.05	0.06	0.09
MLLT6	2369	0.07	0.05	0.03	0.07	0.05	0.04	0.04	0.04	0.06	0.06
PPARG	1042	0.05	0.04	0.04	0.05	0.05	0.05	0.05	0.04	0.07	0.05
HDAC11	971	0.04	0.04	0.04	0.05	0.05	0.05	0.05	0.05	0.06	0.06
LYN	4250	0.04	0.05	0.05	0.04	0.06	0.04	0.05	0.05	0.05	0.07
NKX3-1	1610	0.05	0.06	0.04	0.05	0.05	0.06	0.04	0.04	0.07	0.04
BMP6	1888	0.05	0.05	0.06	0.06	0.05	0.05	0.05	0.04	0.04	0.03
IL17RB	1182	0.05	0.05	0.05	0.05	0.05	0.05	0.05	0.04	0.03	0.03
ONECUT2	6117	0.05	0.05	0.03	0.05	0.04	0.06	0.05	0.04	0.06	0.09
NTSR1	3041	0.07	0.04	0.05	0.05	0.05	0.04	0.05	0.04	0.04	0.04
PLXDC1	2732	0.07	0.06	0.04	0.09	0.05	0.05	0.04	0.04	0.06	0.04
PTK2	5044	0.04	0.06	0.05	0.04	0.06	0.05	0.05	0.05	0.05	0.05
FLT3	4037	0.05	0.06	0.05	0.06	0.05	0.05	0.05	0.05	0.05	0.05
INHA	5800	0.04	0.06	0.05	0.08	0.06	0.05	0.05	0.04	0.04	0.05
INHA	5715	0.05	0.05	0.06	0.05	0.05	0.05	0.05	0.03	0.06	0.08
CDKN2B	4069	0.07	0.05	0.05	0.04	0.05	0.05	0.05	0.05	0.06	0.07
CASP3	3063	0.05	0.05	0.04	0.04	0.06	0.06	0.05	0.06	0.06	0.04
TNFRSF1B	5170	0.05	0.05	0.05	0.12	0.06	0.05	0.06	0.04	0.04	0.04
WNT2B	5394	0.03	0.04	0.05	0.05	0.06	0.05	0.05	0.06	0.06	0.04
NFKB1	4183	0.03	0.05	0.07	0.03	0.04	0.31	0.21	0.05	0.04	0.06
HCK	5769	0.06	0.04	0.05	0.04	0.07	0.06	0.05	0.05	0.05	0.05
BCL3	5500	0.05	0.04	0.05	0.06	0.05	0.05	0.05	0.04	0.04	0.03
FGFR3	4098	0.05	0.05	0.05	0.07	0.06	0.07	0.05	0.05	0.07	0.06
CPNE1	401	0.07	0.05	0.06	0.06	0.05	0.04	0.04	0.03	0.05	0.03
GP1BB	3340	0.09	0.07	0.04	0.09	0.05	0.05	0.05	0.05	0.06	0.08
IGF2	2661	0.05	0.05	0.05	0.06	0.06	0.08	0.05	0.04	0.04	0.1
MGMT	1407	0.07	0.03	0.04	0.05	0.05	0.04	0.06	0.05	0.05	0.05
AHR	3092	0.05	0.05	0.05	0.06	0.06	0.05	0.05	0.06	0.06	0.07
HSD17B12	1093	0.05	0.05	0.05	0.05	0.05	0.05	0.05	0.05	0.04	0.06
GRB10	3349	0.04	0.06	0.06	0.05	0.06	0.06	0.04	0.05	0.06	0.09
CDC25B	3183	0.05	0.05	0.05	0.07	0.07	0.07	0.05	0.05	0.06	0.06
TFPI2	3970	0.06	0.07	0.06	0.03	0.05	0.04	0.04	0.06	0.06	0.07
DKFZP564O0823	2882	0.05	0.06	0.06	0.05	0.06	0.06	0.05	0.05	0.05	0.08
CCND1	70	0.05	0.06	0.04	0.07	0.06	0.06	0.05	0.05	0.05	0.04
GSTP1	2619	0.09	0.06	0.05	0.05	0.05	0.05	0.05	0.05	0.07	0.06
TNFRSF10B	1326	0.05	0.08	0.05	0.07	0.06	0.04	0.07	0.05	0.05	0.05
CTNNA1	3487	0.07	0.07	0.06	0.08	0.05	0.05	0.05	0.05	0.06	0.06
KCNQ1	3536	0.06	0.05	0.05	0.06	0.05	0.07	0.05	0.06	0.06	0.05
SMO	5735	0.05	0.06	0.04	0.06	0.06	0.05	0.06	0.04	0.07	0.04
CSTB	3099	0.06	0.04	0.06	0.07	0.05	0.05	0.07	0.04	0.04	0.03
GFI1	4064	0.06	0.04	0.05	0.07	0.06	0.06	0.05	0.05	0.05	0.07
MAP2K6	5639	0.13	0.01	0.06	0.01	0.07	0.02	0.1	0.05	0.04	0.04
E2F3	3811	0.05	0.05	0.08	0.06	0.06	0.06	0.05	0.06	0.03	0.03
JAG2	4985	0.06	0.05	0.05	0.07	0.08	0.05	0.06	0.04	0.08	0.05
EPHB3	2708	0.05	0.07	0.06	0.15	0.04	0.07	0.03	0.04	0.03	0.06
PYCARD	3856	0.07	0.05	0.06	0.05	0.06	0.06	0.05	0.05	0.08	0.06
TIAM1	5333	0.04	0.05	0.06	0.06	0.06	0.05	0.1	0.04	0.03	0.05
BSG	4248	0.08	0.05	0.06	0.07	0.06	0.05	0.06	0.05	0.05	0.05
WT1	4207	0.04	0.05	0.05	0.06	0.06	0.06	0.06	0.05	0.06	0.06
MGMT	1432	0.09	0.06	0.06	0.05	0.05	0.07	0.05	0.04	0.06	0.04
HIC2	1001	0.05	0.05	0.04	0.06	0.06	0.06	0.06	0.06	0.06	0.05
IGF1R	649	0.06	0.05	0.06	0.06	0.06	0.06	0.05	0.05	0.07	0.08
NOTCH1	4204	0.07	0.04	0.04	0.07	0.07	0.06	0.06	0.03	0.06	0.08
AKT1	3101	0.05	0.06	0.06	0.08	0.06	0.05	0.06	0.05	0.05	0.08
THY1	5973	0.05	0.05	0.06	0.07	0.06	0.04	0.06	0.06	0.06	0.1
WNT5A	1389	0.06	0.05	0.06	0.06	0.07	0.04	0.06	0.05	0.06	0.04
MYB	5817	0.06	0.06	0.06	0.06	0.06	0.06	0.06	0.06	0.05	0.05
CASP2	5508	0.1	0.05	0.07	0.06	0.06	0.05	0.06	0.04	0.05	0.04
ICAM1	4104	0.1	0.06	0.05	0.1	0.05	0.05	0.07	0.04	0.05	0.05
BMP6	1885	0.04	0.1	0.06	0.04	0.09	0.06	0.07	0.06	0.05	0.1
RYK	5179	0.06	0.06	0.06	0.1	0.06	0.06	0.07	0.05	0.06	0.04
ITGA2	2008	0.03	0.06	0.06	0.04	0.06	0.06	0.07	0.04	0.03	0.03
RIPK3	2305	0.1	0.04	0.05	0.05	0.08	0.06	0.07	0.06	0.07	0.03
MCM2	2100	0.08	0.06	0.05	0.07	0.07	0.06	0.05	0.04	0.04	0.04
SMARCA3	2019	0.07	0.08	0.07	0.03	0.06	0.05	0.06	0.05	0.09	0.06
ABCG2	3089	0.06	0.06	0.08	0.06	0.07	0.06	0.06	0.06	0.07	0.09
NEU1	1601	0.07	0.04	0.07	0.06	0.07	0.04	0.06	0.05	0.07	0.05

IPF1	5888	0.122	0.144	0.100	0.075	0.105	0.074	0.072	0.081	0.180	0.424
AREG	3002	0.064	0.085	0.103	0.088	0.097	0.102	0.092	0.091	0.112	0.110
ITGB4	3996	0.122	0.109	0.103	0.079	0.078	0.105	0.081	0.068	0.171	0.196
CSF1	430	0.120	0.075	0.101	0.095	0.091	0.093	0.076	0.075	0.131	0.124
TFPI2	2099	0.090	0.097	0.095	0.086	0.093	0.103	0.092	0.105	0.148	0.169
PLAUR	951	0.100	0.093	0.102	0.090	0.102	0.096	0.093	0.092	0.114	0.070
EPHA8	1851	0.158	0.112	0.093	0.096	0.099	0.082	0.083	0.078	0.098	0.150
RAP1A	4896	0.124	0.066	0.099	0.064	0.078	0.091	0.102	0.101	0.184	0.243
FAT	3988	0.111	0.098	0.083	0.090	0.106	0.093	0.118	0.075	0.112	0.211
MAP3K1	5643	0.111	0.141	0.060	0.114	0.080	0.067	0.127	0.055	0.053	0.252
CFTR	256	0.102	0.093	0.205	0.098	0.083	0.190	0.072	0.069	0.181	0.356
TIAM1	5330	0.125	0.048	0.125	0.085	0.107	0.141	0.078	0.044	0.192	0.118
MBD2	4074	0.255	0.100	0.084	0.111	0.092	0.087	0.109	0.081	0.268	0.434
IHH	4944	0.109	0.096	0.084	0.106	0.120	0.096	0.093	0.095	0.083	0.095
PTGS2	2831	0.091	0.093	0.136	0.080	0.108	0.099	0.104	0.094	0.068	0.128
GLI3	3877	0.158	0.164	0.091	0.102	0.088	0.107	0.088	0.065	0.124	0.101
HTR1B	1102	0.081	0.070	0.143	0.118	0.084	0.111	0.095	0.100	0.055	0.180
GJB2	895	0.099	0.112	0.092	0.102	0.086	0.096	0.092	0.102	0.088	0.113
HOXB13	1078	0.088	0.133	0.146	0.083	0.098	0.101	0.097	0.093	0.074	0.083
PTCH2	5025	0.128	0.089	0.100	0.097	0.100	0.104	0.090	0.084	0.080	0.119
FES	605	0.100	0.104	0.097	0.091	0.111	0.100	0.128	0.077	0.058	0.131
TGFB2	1265	0.110	0.112	0.104	0.088	0.096	0.124	0.093	0.096	0.078	0.149
TMEFF1	2136	0.128	0.094	0.100	0.133	0.102	0.081	0.104	0.058	0.198	0.438
OPCML	1651	0.075	0.151	0.154	0.160	0.081	0.098	0.104	0.082	0.101	0.172
TERT	2885	0.103	0.088	0.106	0.136	0.101	0.097	0.104	0.098	0.090	0.099
COL1A1	3366	0.163	0.116	0.120	0.100	0.108	0.080	0.084	0.088	0.168	0.210
DDB2	3399	0.104	0.108	0.086	0.113	0.111	0.104	0.102	0.089	0.087	0.080
ID1	2256	0.102	0.111	0.069	0.121	0.106	0.080	0.161	0.066	0.082	0.091
CDH1	2350	0.121	0.096	0.108	0.101	0.094	0.126	0.118	0.083	0.112	0.085
FLI1	1337	0.101	0.106	0.096	0.143	0.117	0.119	0.097	0.106	0.106	0.151
SPARC	1222	0.124	0.109	0.092	0.108	0.103	0.079	0.125	0.094	0.084	0.116
NFKB1	4185	0.059	0.118	0.100	0.104	0.109	0.110	0.090	0.111	0.192	0.229
IL6	4118	0.104	0.133	0.112	0.085	0.109	0.116	0.088	0.096	0.228	0.211
COMT	130	0.083	0.083	0.102	0.164	0.127	0.159	0.114	0.086	0.117	0.086
ASCL2	103	0.192	0.100	0.122	0.141	0.101	0.102	0.116	0.072	0.254	0.206
TES	2094	0.107	0.111	0.116	0.111	0.106	0.104	0.147	0.097	0.123	0.152
BCL2L2	5384	0.127	0.076	0.148	0.058	0.095	0.135	0.124	0.077	0.097	0.144
TWIST1	2168	0.110	0.097	0.126	0.084	0.123	0.140	0.108	0.109	0.073	0.086
ALK	3111	0.077	0.071	0.102	0.136	0.132	0.131	0.117	0.055	0.060	0.103
CDH1	2352	0.147	0.110	0.088	0.109	0.148	0.137	0.076	0.106	0.154	0.165
CDH3	3057	0.090	0.110	0.111	0.161	0.119	0.094	0.110	0.083	0.093	0.100
PTHLH	973	0.129	0.137	0.112	0.107	0.110	0.104	0.111	0.105	0.185	0.206
APC	4022	0.111	0.112	0.120	0.091	0.107	0.103	0.116	0.116	0.113	0.158
LAMB1	742	0.132	0.114	0.106	0.099	0.107	0.138	0.112	0.113	0.101	0.105
CD34	3103	0.143	0.114	0.111	0.080	0.121	0.098	0.102	0.128	0.330	0.338
SMAD2	6021	0.088	0.113	0.128	0.107	0.113	0.130	0.135	0.090	0.077	0.143
GPX3	3348	0.158	0.112	0.103	0.115	0.114	0.114	0.095	0.116	0.084	0.105
PLXDC1	2871	0.125	0.119	0.102	0.172	0.121	0.108	0.111	0.097	0.103	0.079
ACVR2B	5823	0.195	0.121	0.109	0.139	0.099	0.111	0.090	0.142	0.103	0.149
SCGB3A1	1883	0.118	0.201	0.115	0.115	0.117	0.131	0.114	0.095	0.107	0.134
DCC	4077	0.116	0.114	0.132	0.092	0.116	0.116	0.123	0.109	0.264	0.408
FN1	815	0.102	0.158	0.159	0.123	0.102	0.109	0.183	0.081	0.108	0.111
ALPL	5851	0.117	0.115	0.110	0.119	0.125	0.083	0.102	0.142	0.178	0.208
GDF10	890	0.122	0.115	0.099	0.138	0.155	0.130	0.108	0.114	0.124	0.140
CD81	223	0.171	0.110	0.113	0.147	0.143	0.120	0.121	0.107	0.171	0.198
DLL1	4317	0.099	0.098	0.111	0.235	0.159	0.131	0.149	0.089	0.165	0.244
EPS8	3878	0.076	0.161	0.120	0.115	0.122	0.131	0.138	0.110	0.098	0.317
EXT1	1324	0.082	0.139	0.124	0.078	0.119	0.124	0.132	0.111	0.108	0.110
CDKN1C	234	0.148	0.121	0.106	0.149	0.160	0.106	0.124	0.114	0.127	0.091
ABO	2986	0.193	0.079	0.115	0.212	0.132	0.101	0.137	0.092	0.065	0.095
IRF5	1225	0.150	0.125	0.135	0.122	0.139	0.122	0.123	0.119	0.144	0.113
HPN	4928	0.238	0.065	0.143	0.149	0.050	0.176	0.110	0.104	0.135	0.179
HOXC6	1080	0.155	0.114	0.141	0.114	0.126	0.136	0.111	0.129	0.148	0.288
INSR	2788	0.128	0.125	0.129	0.252	0.115	0.139	0.148	0.106	0.151	0.144
HLA-DPB1	3440	0.168	0.118	0.136	0.080	0.134	0.128	0.130	0.096	0.155	0.093
PDGFRA	992	0.151	0.133	0.113	0.195	0.133	0.067	0.148	0.092	0.234	0.347
MME	3690	0.110	0.128	0.140	0.131	0.138	0.158	0.136	0.130	0.144	0.335
CTSH	3125	0.156	0.122	0.162	0.134	0.130	0.146	0.133	0.126	0.132	0.136
DLC1	4082	0.145	0.132	0.119	0.104	0.135	0.139	0.150	0.109	0.243	0.306
MLF1	4276	0.174	0.130	0.082	0.146	0.140	0.121	0.139	0.069	0.078	0.104
SH3BP2	1178	0.170	0.120	0.117	0.175	0.137	0.136	0.134	0.121	0.108	0.192
FRZB	623	0.130	0.122	0.133	0.091	0.141	0.171	0.146	0.141	0.132	0.385
LYN	752	0.142	0.190	0.221	0.125	0.131	0.134	0.181	0.124	0.140	0.134
TYK2	5329	0.203	0.139	0.111	0.307	0.145	0.142	0.134	0.096	0.151	0.153
DAB2IP	3132	0.169	0.135	0.129	0.156	0.122	0.152	0.146	0.120	0.080	0.113
PENK	3752	0.164	0.144	0.140	0.149	0.122	0.130	0.142	0.098	0.108	0.202
ITGB1	4227	0.097	0.165	0.136	0.151	0.147	0.138	0.145	0.111	0.101	0.112
TMEFF2	2137	0.132	0.137	0.156	0.141	0.143	0.143	0.143	0.120	0.131	0.165
PTPRF	977	0.178	0.217	0.076	0.080	0.081	0.235	0.153	0.131	0.099	0.186
ELK3	3501	0.114	0.133	0.140	0.149	0.145	0.148	0.163	0.127	0.089	0.276
CDK6	205	0.126	0.151	0.149	0.122	0.154	0.163	0.148	0.141	0.160	0.137
DUSP4	2896	0.138	0.148	0.156	0.101	0.150	0.191	0.152	0.145	0.170	0.187
ZNF215	2215	0.188	0.127	0.132	0.215	0.161	0.137	0.177	0.121	0.179	0.278
FLT4	559	0.188	0.168	0.146	0.091	0.155	0.156	0.136	0.154	0.099	0.130
COL6A1	1998	0.192	0.160	0.153	0.240	0.182	0.137	0.149	0.111	0.115	0.117
IL11	721	0.130	0.158	0.146	0.186	0.180	0.166	0.156	0.154	0.193	0.159
MCAM	4265	0.129	0.168	0.152	0.198	0.151	0.184	0.164	0.134	0.162	0.142
BCL3	1549	0.182	0.222	0.143	0.172	0.149	0.152	0.165	0.144	0.199	0.287
WRN	1462	0.186	0.161	0.168	0.166	0.158	0.080	0.150	0.086	0.202	0.188
DAB2IP	1562	0.200	0.158	0.158	0.195	0.186	0.151	0.149	0.163	0.134	0.150
GUCY2D	1950	0.225	0.174	0.122	0.154	0.194	0.168	0.143	0.128	0.155	0.129
SLC5A8	3930	0.173	0.180	0.180	0.141	0.153	0.170	0.143	0.136	0.129	0.239
HS3ST2	1087	0.189	0.175	0.157	0.172	0.133	0.168	0.137	0.131	0.122	0.193
EPS8	296	0.174	0.156	0.196	0.154	0.193	0.176	0.137	0.153	0.124	0.205
IGFBP3	4115	0.151	0.175	0.188	0.160	0.174	0.166	0.169	0.166	0.192	0.175
DAPK1	4075	0.161	0.124	0.205	0.173	0.163	0.194	0.176	0.148	0.186	0.185
HOXC6	1079	0.159	0.167	0.169	0.149	0.169	0.174	0.173	0.170	0.171	0.227
CDH3	230	0.170	0.185	0.172	0.152	0.168	0.205	0.159	0.164	0.140	0.182
TES	3968	0.154	0.193	0.150	0.081	0.190	0.205	0.186	0.098	0.086	0.083
GABRB3	861	0.159	0.146	0.178	0.176	0.174	0.173	0.168	0.158	0.159	0.204
HDAC9	984	0.147	0.175	0.376	0.223	0.125	0.166	0.192	0.130	0.196	0.241
BMP4	3156	0.181	0.194	0.166	0.131	0.148	0.177	0.184	0.150	0.108	0.198
HDAC9	3393	0.148	0.195	0.320	0.279	0.109	0.133	0.210	0.085	0.096	0.199
ARHGDIB	3126	0.310	0.171	0.146	0.169	0.222	0.172	0.216	0.167	0.305	0.324
DIO3	634	0.166	0.216	0.181	0.155	0.178	0.213	0.143	0.150	0.162	0.290
NEFL	1596	0.187	0.162	0.168	0.159	0.178	0.217	0.156	0.180	0.087	0.264
SCGB3A1	3888	0.189	0.164	0.176	0.208	0.162	0.178	0.172	0.154	0.157	0.183
IRAK3	4954	0.163	0.242	0.192	0.196	0.116	0.212	0.122	0.162	0.056	0.260
GDF10	3315	0.174	0.153	0.185	0.152	0.190	0.187	0.190	0.146	0.203	0.177
CASP8	4042	0.295	0.205	0.185	0.155	0.173	0.172	0.242	0.174	0.106	0.140
SEMA3A	2799	0.327	0.184	0.180	0.106	0.238	0.080	0.329	0.106	0.059	0.162
FGFR1	620	0.237	0.207	0.144	0.271	0.199	0.155	0.167	0.157	0.130	0.122
FGF9	4026	0.181	0.204	0.226	0.160	0.163	0.200	0.186	0.175	0.138	0.208
TSG101	6052	0.211	0.129	0.210	0.272	0.159	0.206	0.173	0.165	0.171	0.081
SLIT2	5725	0.192	0.200	0.145	0.211	0.187	0.203	0.190	0.152	0.160	0.261
MATK	5839	0.298	0.203	0.276	0.290	0.179	0.165	0.168	0.129	0.114	0.368
CD34	122	0.183	0.165	0.204	0.212	0.229	0.177	0.205	0.156	0.104	0.198
FGF9	4028	0.189	0.196	0.189	0.186	0.215	0.214	0.217	0.195	0.224	0.209
ADAMTS12	2989	0.252	0.202	0.194	0.219	0.185	0.189	0.252	0.171	0.062	0.170
IGFBP6	654	0.271	0.174	0.196	0.164	0.220	0.144	0.222	0.200	0.345	0.332
GNMT	917	0.194	0.159	0.203	0.249	0.209	0.180	0.203	0.127	0.151	0.309
NPY	3730	0.229	0.201	0.203	0.202	0.202	0.210	0.235	0.139	0.154	0.306
F2R	764	0.198	0.190	0.213	0.166	0.229	0.212	0.231	0.157	0.158	0.383
PDGFB	1680	0.277	0.230	0.168	0.213	0.198	0.215	0.136	0.175	0.165	0.236
NOTCH3	5088	0.258	0.221	0.217	0.377	0.197	0.173	0.132	0.078	0.110	0.325
DSP	3725	0.123	0.209	0.144	0.244	0.231	0.207	0.209	0.139	0.076	0.153
FGFR2	2501	0.244	0.186	0.202	0.260	0.184	0.216	0.233	0.150	0.150	0.334
ABL2	4216	0.180	0.253	0.226	0.183	0.208	0.211	0.227	0.212	0.114	0.229
HDAC5	3380	0.274	0.202	0.194	0.277	0.231	0.191	0.227	0.153	0.231	0.309
ETV6	442	0.207	0.192	0.226	0.263	0.201	0.260	0.176	0.225	0.247	0.215
PEG3	3746	0.262	0.171	0.214	0.249	0.232	0.177	0.261	0.161	0.325	0.295
SPARC	5009	0.314	0.328	0.201	0.252	0.219	0.155	0.227	0.187	0.126	0.304
MUC1	5784	0.291	0.184	0.237	0.520	0.216	0.091	0.231	0.189	0.115	0.073
THY1	5968	0.179	0.101	0.287	0.281	0.173	0.272	0.079	0.295	0.178	0.204
EGR4	3191	0.284	0.245	0.237	0.253	0.229	0.221	0.229	0.177	0.163	0.279
IRF7	3520	0.264	0.224	0.238	0.273	0.235	0.181	0.239	0.150	0.103	0.158
ASCL2	3019	0.275	0.233	0.249	0.327	0.238	0.292	0.226	0.214	0.180	0.175
H19	949	0.374	0.235	0.408	0.323	0.136	0.263	0.233	0.177	0.139	0.218
IGF2AS	3495	0.262	0.286	0.278	0.273	0.211	0.227	0.243	0.228	0.156	0.225
TK1	1097	0.231	0.259	0.282	0.283	0.248	0.223	0.338	0.207	0.118	0.162
BCL2L2	4235	0.572	0.175	0.439	0.200	0.266	0.243	0.399	0.218	0.172	0.279
TRAF4	6080	0.215	0.284	0.286	0.191	0.215	0.336	0.252	0.272	0.150	0.201

PWCR1	1789	0.88	0.89	0.87	0.84	0.84	0.87	0.86	0.9	0.86	0.82
LTA	820	0.88	0.94	0.94	0.84	0.86	0.83	0.96	0.86	0.77	0.63
IGF1	4132	0.86	0.87	0.89	0.9	0.88	0.85	0.89	0.87	0.6	0.53
ACTG2	33	0.89	0.87	0.89	0.89	0.86	0.86	0.86	0.89	0.92	0.85
INS	1219	0.85	0.9	0.9	0.81	0.84	0.88	0.88	0.89	0.86	0.81
DSG1	2890	0.91	0.88	0.88	0.93	0.86	0.87	0.88	0.86	0.82	0.82
SFTPA1	1949	0.9	0.9	0.89	0.9	0.85	0.85	0.88	0.84	0.76	0.6
PI3	1685	0.88	0.88	0.91	0.91	0.87	0.86	0.87	0.88	0.9	0.66
TRPM5	3986	0.88	0.9	0.89	0.86	0.88	0.88	0.88	0.91	0.92	0.86
LEFTY2	5906	0.86	0.89	0.89	0.89	0.87	0.87	0.88	0.89	0.9	0.71
CSF3	3453	0.85	0.91	0.9	0.88	0.88	0.89	0.88	0.88	0.71	0.67
CHI3L2	3077	0.88	0.89	0.89	0.92	0.88	0.89	0.87	0.88	0.73	0.75
SPI1	5021	0.88	0.89	0.89	0.87	0.88	0.87	0.89	0.89	0.91	0.8
EPHX1	2939	0.89	0.89	0.89	0.88	0.88	0.85	0.87	0.89	0.87	0.71
SNCG	3934	0.88	0.89	0.9	0.9	0.89	0.88	0.88	0.88	0.69	0.81
TRPM5	2155	0.89	0.89	0.88	0.86	0.89	0.89	0.89	0.9	0.92	0.88
S100A2	3887	0.85	0.89	0.89	0.87	0.89	0.89	0.89	0.89	0.46	0.52
SFTPB	1951	0.88	0.89	0.89	0.9	0.88	0.89	0.9	0.9	0.64	0.79
BLK	154	0.89	0.91	0.9	0.92	0.86	0.89	0.89	0.88	0.87	0.8
LRRK1	2321	0.88	0.91	0.89	0.89	0.9	0.89	0.91	0.9	0.9	0.9
KRT5	2049	0.89	0.9	0.89	0.9	0.9	0.89	0.9	0.88	0.89	0.86
FGF1	619	0.89	0.9	0.9	0.91	0.89	0.88	0.89	0.91	0.92	0.86
LEFTY2	5899	0.92	0.9	0.92	0.9	0.89	0.87	0.89	0.89	0.89	0.88
NOS3	4201	0.9	0.92	0.67	0.89	0.89	0.62	0.9	0.91	0.89	0.82
IL2	4205	0.89	0.87	0.92	0.9	0.91	0.89	0.9	0.89	0.91	0.86
CPA4	368	0.91	0.91	0.89	0.92	0.88	0.88	0.89	0.91	0.9	0.89
S100A4	5985	0.88	0.82	0.9	0.9	0.9	0.88	0.9	0.92	0.9	0.89
SNCG	2024	0.91	0.9	0.87	0.9	0.9	0.89	0.9	0.89	0.46	0.75
PRDM2	1759	0.86	0.92	0.91	0.92	0.9	0.85	0.89	0.89	0.93	0.9
PADI4	1655	0.9	0.9	0.9	0.89	0.89	0.9	0.91	0.91	0.78	0.59
IL18BP	4211	0.88	0.91	0.9	0.91	0.89	0.9	0.9	0.91	0.76	0.79
MMP3	4851	0.91	0.91	0.91	0.92	0.9	0.89	0.9	0.89	0.91	0.9
GLI2	4094	0.88	0.91	0.92	0.9	0.9	0.91	0.89	0.92	0.93	0.9
CSF2	3448	0.93	0.89	0.9	0.93	0.9	0.9	0.91	0.91	0.9	0.89
IGF1	648	0.88	0.89	0.92	0.91	0.89	0.91	0.92	0.91	0.83	0.75
MMP8	3694	0.89	0.92	0.91	0.95	0.91	0.9	0.92	0.91	0.65	0.75
IFNG	5790	0.92	0.91	0.91	0.87	0.92	0.86	0.91	0.91	0.91	0.75
SFTPD	3920	0.92	0.92	0.91	0.92	0.91	0.9	0.9	0.92	0.89	0.88
PMP22	1718	0.91	0.92	0.91	0.92	0.91	0.91	0.92	0.91	0.93	0.92
PTHR1	1772	0.91	0.93	0.92	0.92	0.91	0.91	0.91	0.92	0.6	0.63
PLAT	1041	0.92	0.92	0.92	0.92	0.91	0.9	0.91	0.91	0.86	0.83
PLG	952	0.93	0.91	0.91	0.91	0.92	0.89	0.92	0.92	0.95	0.92
THPO	5322	0.94	0.92	0.91	0.91	0.92	0.89	0.92	0.93	0.92	0.93
CHD2	261	0.94	0.94	0.91	0.92	0.92	0.87	0.93	0.91	0.94	0.92
MC2R	3630	0.93	0.93	0.91	0.93	0.92	0.91	0.92	0.92	0.93	0.92
SLC22A18	1974	0.89	0.93	0.92	0.9	0.92	0.92	0.9	0.92	0.73	0.86
DMP1	3153	0.93	0.91	0.92	0.93	0.9	0.9	0.92	0.92	0.9	0.89
TRPM5	2153	0.92	0.94	0.92	0.92	0.92	0.91	0.93	0.93	0.89	0.88
PRSS1	3060	0.92	0.93	0.92	0.93	0.91	0.91	0.93	0.91	0.89	0.87
PADI4	1658	0.92	0.93	0.93	0.92	0.92	0.92	0.92	0.93	0.79	0.68
CLDN4	4287	0.91	0.93	0.92	0.92	0.92	0.91	0.93	0.93	0.9	0.77
MPO	3028	0.89	0.93	0.92	0.91	0.92	0.92	0.92	0.94	0.76	0.64
CTLA4	3115	0.94	0.93	0.92	0.94	0.92	0.91	0.92	0.92	0.9	0.85
TRIP6	2402	0.91	0.93	0.92	0.93	0.92	0.91	0.92	0.94	0.31	0.69
MAPK4	5660	0.91	0.93	0.93	0.94	0.92	0.92	0.93	0.91	0.78	0.83
TNFRSF10C	2144	0.95	0.94	0.9	0.89	0.92	0.89	0.93	0.93	0.92	0.9
GFAP	5878	0.92	0.92	0.93	0.93	0.93	0.92	0.92	0.94	0.94	0.89
IL1RN	4198	0.92	0.92	0.94	0.94	0.93	0.92	0.93	0.92	0.93	0.88
ZNFN1A1	1653	0.93	0.93	0.93	0.94	0.92	0.92	0.92	0.93	0.93	0.9
HBII-13	3370	0.93	0.94	0.93	0.92	0.92	0.91	0.93	0.94	0.86	0.88
PI3	3778	0.92	0.93	0.93	0.95	0.86	0.85	0.94	0.93	0.77	0.72
ZAP70	5350	0.93	0.92	0.95	0.93	0.93	0.91	0.94	0.93	0.62	0.37
ACTG2	2988	0.94	0.93	0.94	0.94	0.93	0.92	0.93	0.92	0.91	0.86
HGF	1961	0.95	0.94	0.93	0.94	0.93	0.91	0.93	0.93	0.94	0.93
CDH17	4279	0.95	0.93	0.93	0.94	0.91	0.93	0.93	0.92	0.92	0.91
LTA	4046	0.93	0.93	0.93	0.92	0.93	0.93	0.94	0.94	0.87	0.73
IFNG	5788	0.93	0.93	0.92	0.91	0.94	0.89	0.94	0.93	0.88	0.77
PMP22	1730	0.94	0.93	0.93	0.93	0.93	0.92	0.93	0.93	0.89	0.81
PTHLH	4301	0.93	0.93	0.93	0.94	0.94	0.92	0.94	0.93	0.78	0.83
BCL2A1	3039	0.93	0.94	0.88	0.88	0.93	0.8	0.95	0.94	0.97	0.96
DMP1	651	0.94	0.93	0.93	0.96	0.93	0.94	0.94	0.93	0.91	0.86
CD1A	206	0.94	0.94	0.93	0.94	0.93	0.93	0.94	0.94	0.9	0.87
RUNX3	1870	0.93	0.94	0.94	0.92	0.94	0.94	0.94	0.95	0.89	0.67
SRC	6034	0.94	0.94	0.94	0.93	0.93	0.93	0.94	0.94	0.93	0.94
HTR2A	3493	0.95	0.94	0.94	0.95	0.93	0.92	0.94	0.93	0.94	0.92
TFF1	5244	0.94	0.94	0.94	0.94	0.93	0.93	0.94	0.94	0.93	0.91
MMP19	1559	0.94	0.94	0.94	0.94	0.93	0.94	0.94	0.94	0.89	0.82
PWCR1	1790	0.94	0.93	0.95	0.93	0.94	0.95	0.94	0.94	0.93	0.91
FGF7	5625	0.95	0.95	0.94	0.96	0.93	0.93	0.94	0.93	0.92	0.92
CSF3	215	0.92	0.95	0.93	0.94	0.94	0.94	0.94	0.95	0.58	0.78
APOA1	63	0.96	0.95	0.92	0.92	0.92	0.93	0.94	0.94	0.87	0.9
IL1B	3947	0.93	0.94	0.94	0.94	0.94	0.93	0.95	0.94	0.88	0.85
INS	1203	0.93	0.94	0.95	0.94	0.94	0.94	0.95	0.95	0.94	0.94
PTHR1	1775	0.94	0.94	0.94	0.94	0.94	0.94	0.94	0.95	0.94	0.94
TGFB3	1269	0.96	0.95	0.94	0.94	0.94	0.9	0.93	0.94	0.97	0.95
CHI3L2	300	0.93	0.94	0.94	0.96	0.94	0.94	0.95	0.95	0.73	0.71
FGF1	3718	0.94	0.95	0.94	0.96	0.94	0.94	0.93	0.94	0.93	0.88
RUNX3	3879	0.94	0.95	0.95	0.93	0.94	0.94	0.94	0.95	0.91	0.8
SOD3	2043	0.93	0.95	0.95	0.96	0.94	0.94	0.93	0.92	0.89	0.71
SLC14A1	5724	0.94	0.95	0.94	0.95	0.94	0.94	0.94	0.94	0.77	0.77
PLA2G2A	4976	0.94	0.95	0.94	0.94	0.94	0.94	0.95	0.94	0.86	0.71
TFF2	5260	0.94	0.95	0.94	0.94	0.94	0.95	0.95	0.95	0.88	0.86
IL1RN	745	0.95	0.92	0.95	0.94	0.93	0.94	0.95	0.93	0.94	0.93
CSF2	213	0.95	0.94	0.95	0.96	0.94	0.93	0.95	0.94	0.91	0.91
MMP10	920	0.94	0.95	0.94	0.96	0.95	0.94	0.95	0.94	0.89	0.51
PTPRH	5067	0.94	0.96	0.94	0.93	0.94	0.95	0.95	0.95	0.95	0.95
APBA2	62	0.95	0.95	0.95	0.94	0.94	0.93	0.95	0.94	0.9	0.84
AIM2	1228	0.95	0.95	0.96	0.95	0.94	0.94	0.95	0.95	0.91	0.85
AFP	5798	0.96	0.95	0.95	0.95	0.95	0.94	0.94	0.94	0.94	0.92
CSF3R	3461	0.92	0.95	0.94	0.95	0.95	0.94	0.95	0.95	0.28	0.61
B3GALT5	1844	0.95	0.95	0.95	0.96	0.94	0.95	0.95	0.95	0.95	0.91
KRT1	5013	0.94	0.95	0.95	0.96	0.95	0.94	0.95	0.94	0.89	0.84
NOTCH4	4208	0.93	0.95	0.95	0.96	0.95	0.94	0.95	0.94	0.85	0.67
KRT13	2312	0.95	0.95	0.95	0.94	0.95	0.95	0.94	0.95	0.95	0.93
WNT8B	1650	0.94	0.95	0.95	0.96	0.94	0.95	0.95	0.94	0.91	0.87
KRT13	2297	0.91	0.95	0.95	0.94	0.95	0.95	0.96	0.95	0.8	0.72
PROM1	5933	0.96	0.95	0.95	0.96	0.94	0.95	0.95	0.94	0.92	0.83
EGF	3497	0.95	0.95	0.95	0.97	0.95	0.94	0.95	0.94	0.83	0.88
APBA2	61	0.95	0.96	0.96	0.95	0.94	0.95	0.95	0.95	0.92	0.85
PIK3R1	2632	0.95	0.96	0.95	0.96	0.95	0.95	0.96	0.94	0.91	0.85
CXCL9	2584	0.96	0.96	0.95	0.96	0.94	0.93	0.96	0.94	0.93	0.86
MAPK9	5073	0.95	0.95	0.95	0.95	0.95	0.95	0.95	0.95	0.94	0.94
MMP3	4850	0.96	0.95	0.95	0.97	0.94	0.95	0.95	0.95	0.94	0.93
AATK	2976	0.95	0.97	0.96	0.95	0.95	0.95	0.94	0.96	0.96	0.94
EGF	3814	0.96	0.95	0.95	0.96	0.95	0.94	0.95	0.95	0.91	0.81
HLA-DOB	1015	0.96	0.95	0.95	0.96	0.95	0.94	0.95	0.95	0.95	0.93
ABCC2	67	0.95	0.94	0.95	0.95	0.95	0.91	0.95	0.95	0.95	0.95
PTHR1	3833	0.95	0.96	0.95	0.96	0.94	0.95	0.95	0.95	0.93	0.9
NAT2	4177	0.95	0.96	0.95	0.97	0.95	0.94	0.95	0.95	0.84	0.83
S100A2	1876	0.95	0.96	0.95	0.95	0.95	0.95	0.95	0.96	0.91	0.9
MMP9	935	0.95	0.96	0.95	0.95	0.95	0.95	0.96	0.96	0.89	0.86
NOS2A	2161	0.94	0.96	0.95	0.95	0.95	0.95	0.96	0.96	0.9	0.76
HDAC7A	978	0.95	0.96	0.96	0.95	0.95	0.95	0.96	0.95	0.94	0.93
FGF7	5679	0.96	0.95	0.95	0.97	0.95	0.95	0.96	0.95	0.94	0.91
MAS1	2738	0.93	0.95	0.96	0.96	0.96	0.95	0.94	0.95	0.96	0.95
LCN2	5739	0.92	0.95	0.95	0.96	0.96	0.95	0.96	0.96	0.54	0.79
RIPK1	5140	0.96	0.96	0.95	0.97	0.95	0.95	0.95	0.95	0.94	0.9
SLC22A18	1980	0.92	0.96	0.96	0.95	0.95	0.96	0.95	0.96	0.3	0.59
CPA4	377	0.96	0.96	0.95	0.96	0.95	0.95	0.96	0.95	0.94	0.93
SPDEF	2048	0.93	0.95	0.96	0.96	0.95	0.95	0.96	0.96	0.53	0.52
ABCB4	23	0.96	0.96	0.95	0.97	0.95	0.95	0.96	0.95	0.93	0.92
DDR2	4305	0.96	0.96	0.96	0.97	0.95	0.95	0.96	0.95	0.93	0.9
ASB4	3011	0.96	0.96	0.95	0.96	0.95	0.95	0.97	0.95	0.95	0.93
KIAA0125	2797	0.95	0.95	0.96	0.96	0.95	0.96	0.96	0.96	0.94	0.92
HBII-13	960	0.95	0.96	0.95	0.97	0.96	0.96	0.95	0.96	0.91	0.87
RHOH	5169	0.96	0.96	0.95	0.97	0.95	0.95	0.96	0.95	0.94	0.92
GABRA5	840	0.95	0.96	0.96	0.97	0.95	0.96	0.95	0.96	0.92	0.9
IL3	4217	0.96	0.96	0.96	0.96	0.95	0.95	0.96	0.96	0.95	0.95
RIPK1	5133	0.96	0.96	0.96	0.96	0.95	0.96	0.96	0.96	0.92	0.85
LY6G6E	1321	0.96	0.96	0.96	0.95	0.96	0.95	0.96	0.96	0.95	0.91
ASB4	87	0.96	0.96	0.96	0.97	0.96	0.95	0.96	0.94	0.95	0.94
AIM2	5378	0.95	0.96	0.96	0.96	0.95	0.95	0.96	0.96	0.36	0.42

MME	1553	0.12	0.08	0.05	0.07	0.04	0.04	0.07	0.04	0.05	0.06
NGFR	940	0.03	0.05	0.09	0.07	0.07	0.05	0.06	0.07	0.05	0.07
PTPRO	1786	0.05	0.11	0.04	0.09	0.07	0.07	0.06	0.05	0.07	0.08
ASCL1	3014	0.07	0.07	0.06	0.06	0.06	0.07	0.05	0.07	0.05	0.05
CAV1	2326	0.05	0.07	0.05	0.09	0.08	0.06	0.08	0.06	0.06	0.07
ETS2	3931	0.08	0.08	0.07	0.06	0.09	0.05	0.06	0.05	0.05	0.06
THBS1	2110	0.05	0.03	0.07	0.04	0.08	0.07	0.07	0.07	0.04	0.03
ROR2	3066	0.04	0.07	0.05	0.15	0.06	0.07	0.08	0.05	0.05	0.08
KIT	4020	0.06	0.06	0.07	0.09	0.07	0.07	0.07	0.06	0.07	0.07
MCC	4270	0.06	0.07	0.08	0.06	0.07	0.07	0.08	0.07	0.06	0.07
TP73	2930	0.08	0.06	0.07	0.09	0.05	0.07	0.07	0.05	0.05	0.07
CHFR	273	0.08	0.07	0.06	0.03	0.07	0.05	0.07	0.06	0.05	0.05
IFNGR2	697	0.07	0.07	0.05	0.07	0.08	0.06	0.06	0.06	0.07	0.07
PLAU	1702	0.03	0.04	0.09	0.07	0.05	0.09	0.07	0.07	0.1	0.05
NOTCH1	958	0.07	0.06	0.06	0.08	0.07	0.07	0.06	0.05	0.07	0.06
PCGF4	2555	0.05	0.05	0.03	0.16	0.11	0.08	0.08	0.05	0.05	0.04
DAPK1	2420	0.08	0.04	0.06	0.1	0.06	0.08	0.08	0.05	0.05	0.09
CEBPA	3305	0.07	0.07	0.07	0.13	0.08	0.06	0.09	0.05	0.07	0.08
TJP2	2114	0.18	0.07	0.04	0.09	0.07	0.07	0.06	0.04	0.07	0.04
RASGRF1	1826	0.09	0.06	0.05	0.09	0.08	0.09	0.06	0.05	0.07	0.05
THBS2	4189	0.09	0.06	0.09	0.09	0.05	0.05	0.05	0.08	0.07	0.07
HHIP	2760	0.05	0.08	0.14	0.06	0.09	0.08	0.05	0.06	0.08	0.06
IGSF4C	1145	0.08	0.07	0.06	0.08	0.07	0.06	0.06	0.07	0.07	0.09
PLAU	1707	0.06	0.07	0.05	0.1	0.07	0.08	0.08	0.07	0.06	0.06
FGF5	453	0.1	0.07	0.08	0.05	0.06	0.07	0.09	0.06	0.05	0.06
OPCML	3737	0.05	0.06	0.07	0.08	0.07	0.08	0.08	0.07	0.03	0.09
GATA6	5858	0.07	0.07	0.08	0.08	0.08	0.07	0.08	0.05	0.05	0.05
TP73	4200	0.09	0.07	0.08	0.12	0.09	0.07	0.07	0.07	0.05	0.06
KLF5	3539	0.09	0.06	0.06	0.08	0.08	0.09	0.09	0.06	0.04	0.08
MXI1	4857	0.11	0.06	0.1	0.08	0.08	0.06	0.12	0.05	0.04	0.05
IFNGR1	4119	0.08	0.07	0.08	0.06	0.08	0.1	0.07	0.08	0.08	0.06
CHFR	270	0.07	0.07	0.08	0.07	0.08	0.08	0.08	0.08	0.05	0.04
PKD2	5129	0.1	0.08	0.08	0.07	0.08	0.08	0.07	0.08	0.06	0.08
VAV2	3069	0.1	0.08	0.08	0.12	0.08	0.08	0.08	0.06	0.05	0.07
KLF5	1270	0.08	0.09	0.09	0.07	0.08	0.07	0.08	0.09	0.06	0.07
F2R	775	0.07	0.1	0.11	0.07	0.08	0.08	0.11	0.06	0.07	0.06
DST	5534	0.08	0.08	0.1	0.1	0.08	0.09	0.08	0.07	0.05	0.07
BMP3	2863	0.08	0.09	0.08	0.07	0.09	0.08	0.07	0.1	0.08	0.08
TNFRSF10A	5145	0.09	0.08	0.08	0.07	0.05	0.11	0.1	0.06	0.03	0.05
NPY	1644	0.09	0.06	0.09	0.08	0.09	0.09	0.08	0.08	0.05	0.06
PKD2	5127	0.07	0.09	0.08	0.08	0.1	0.1	0.09	0.09	0.05	0.08
FVT1	4044	0.1	0.09	0.09	0.1	0.09	0.08	0.08	0.08	0.06	0.07
EIF2AK2	1319	0.07	0.08	0.09	0.09	0.1	0.09	0.1	0.08	0.06	0.08
JAG1	4977	0.09	0.1	0.1	0.08	0.08	0.1	0.11	0.08	0.06	0.08
MYBL2	4167	0.14	0.08	0.08	0.1	0.1	0.1	0.09	0.07	0.04	0.07
MAF	859	0.1	0.13	0.06	0.15	0.15	0.09	0.07	0.07	0.08	0.05
PCGF4	2182	0.1	0.09	0.1	0.09	0.08	0.15	0.13	0.07	0.04	0.08
CCNE1	3096	0.12	0.12	0.09	0.1	0.1	0.11	0.13	0.1	0.07	0.06
TIMP2	5345	0.14	0.12	0.15	0.13	0.09	0.1	0.07	0.06	0.04	0.07
EPO	4087	0.17	0.09	0.12	0.21	0.11	0.11	0.08	0.08	0.09	0.06
ESR2	756	0.15	0.11	0.13	0.12	0.11	0.1	0.17	0.1	0.08	0.07
BMPR2	2867	0.1	0.12	0.12	0.1	0.12	0.12	0.11	0.11	0.07	0.06
ITGA6	2281	0.12	0.36	0.17	0.13	0.12	0.09	0.15	0.09	0.05	0.09
PURA	5960	0.04	0.19	0.12	0.13	0.22	0.05	0.29	0.05	0.05	0.04
IMPACT	1190	0.14	0.13	0.13	0.11	0.13	0.15	0.12	0.12	0.03	0.03
CYP1B1	185	0.13	0.12	0.11	0.2	0.13	0.14	0.12	0.07	0.07	0.08
MLH3	1543	0.14	0.14	0.13	0.09	0.11	0.13	0.13	0.11	0.04	0.09
MLH3	3686	0.15	0.15	0.17	0.15	0.18	0.18	0.16	0.16	0.04	0.07
HLA-DPA1	1022	0.44	0.2	0.12	0.15	0.24	0.16	0.47	0.33	0.1	0.06
HLA-DPA1	1020	0.44	0.25	0.15	0.17	0.29	0.12	0.52	0.39	0.07	0.03
RHOH	5163	0.92	0.62	0.41	0.66	0.72	0.47	0.59	0.41	0.07	0.04
HTR2A	1105	0.8	0.71	0.62	0.47	0.72	0.57	0.65	0.57	0.06	0.05
SFN	1942	0.76	0.68	0.69	0.68	0.65	0.6	0.63	0.66	0.08	0.05
WNT10B	5383	0.62	0.68	0.71	0.72	0.71	0.68	0.71	0.75	0.1	0.04
TNF	2915	0.59	0.72	0.7	0.82	0.81	0.83	0.81	0.79	0.03	0.02
SPP1	1241	0.86	0.87	0.87	0.88	0.87	0.84	0.86	0.87	0.07	0.07
VAV1	6062	0.83	0.85	0.89	0.86	0.89	0.91	0.86	0.91	0.09	0.05
IL18BP	4209	0.86	0.91	0.91	0.92	0.91	0.91	0.91	0.91	0.06	0.05
CAPG	3045	0.89	0.92	0.92	0.94	0.92	0.92	0.92	0.92	0.03	0.03
VAV1	1645	0.94	0.86	0.92	0.89	0.95	0.96	0.91	0.95	0.05	0.04
CASP10	3056	0.92	0.93	0.93	0.95	0.93	0.93	0.94	0.93	0.04	0.1
VAMP8	2187	0.91	0.93	0.93	0.95	0.94	0.93	0.94	0.94	0.03	0.04
TNFSF10	1109	0.93	0.94	0.94	0.96	0.94	0.94	0.94	0.94	0.06	0.04
PDGFRB	2808	0.91	0.95	0.94	0.95	0.94	0.94	0.95	0.95	0.03	0.03
OSM	5894	0.9	0.95	0.94	0.95	0.95	0.94	0.95	0.95	0.06	0.03
CEACAM1	113	0.9	0.95	0.96	0.95	0.96	0.96	0.95	0.97	0.04	0.04
P2RX7	2854	0.93	0.96	0.95	0.96	0.96	0.95	0.96	0.96	0.06	0.07
THBS2	2907	0.94	0.97	0.34	0.97	0.96	0.96	0.97	0.97	0.08	0.07
EVI2A	3965	0.95	0.96	0.96	0.97	0.96	0.97	0.97	0.97	0.07	0.04
OSM	5910	0.94	0.97	0.96	0.96	0.97	0.97	0.97	0.97	0.05	0.05

PCDH1	2174	0.255	0.039	0.392	0.337	0.276	0.069	0.279	0.244	0.335	0.072
PTCH2	4292	0.224	0.293	0.292	0.400	0.259	0.273	0.259	0.208	0.141	0.469
FER	581	0.272	0.273	0.196	0.281	0.316	0.238	0.270	0.234	0.083	0.239
ERCC6	3906	0.352	0.279	0.264	0.202	0.248	0.286	0.284	0.249	0.135	0.216
NGFB	2157	0.224	0.261	0.320	0.167	0.288	0.284	0.290	0.258	0.169	0.154
MLF1	891	0.406	0.284	0.218	0.352	0.314	0.247	0.277	0.167	0.127	0.264
CYP1B1	3526	0.455	0.267	0.345	0.245	0.331	0.246	0.273	0.290	0.125	0.232
IGFBP7	1124	0.286	0.316	0.238	0.320	0.287	0.281	0.259	0.243	0.122	0.254
KCNQ1	1267	0.269	0.280	0.291	0.330	0.289	0.278	0.288	0.246	0.279	0.280
STAT5A	5736	0.365	0.277	0.346	0.309	0.311	0.244	0.264	0.235	0.184	0.152
PDGFA	5926	0.371	0.287	0.301	0.361	0.324	0.283	0.284	0.292	0.226	0.198
SEMA3A	2804	0.356	0.297	0.315	0.147	0.363	0.150	0.403	0.197	0.219	0.371
CD34	3105	0.418	0.389	0.343	0.289	0.310	0.144	0.180	0.305	0.194	0.246
EPHB4	1590	0.386	0.333	0.290	0.316	0.324	0.322	0.297	0.287	0.159	0.317
ETS1	341	0.303	0.355	0.320	0.336	0.319	0.314	0.360	0.281	0.241	0.242
STK11	2879	0.292	0.405	0.299	0.583	0.335	0.344	0.239	0.314	0.406	0.281
RUNX1T1	5176	0.308	0.343	0.327	0.451	0.376	0.302	0.371	0.259	0.270	0.303
BCAM	140	0.394	0.340	0.330	0.377	0.134	0.347	0.203	0.258	0.096	0.281
COL18A1	3245	0.391	0.340	0.293	0.409	0.334	0.334	0.378	0.211	0.121	0.256
OAT	1647	0.445	0.359	0.368	0.330	0.305	0.338	0.366	0.348	0.172	0.279
MYLK	5669	0.363	0.386	0.375	0.348	0.339	0.340	0.362	0.311	0.233	0.344
EPO	2436	0.379	0.383	0.375	0.352	0.444	0.389	0.346	0.268	0.294	0.385
PTCH2	969	0.447	0.402	0.403	0.382	0.363	0.385	0.366	0.284	0.286	0.436
PTHLH	5037	0.412	0.446	0.291	0.437	0.371	0.310	0.493	0.378	0.339	0.395
FZD9	4876	0.411	0.411	0.384	0.537	0.378	0.400	0.407	0.295	0.154	0.296
TAL1	5047	0.259	0.760	0.200	0.224	0.787	0.254	0.665	0.705	0.244	0.356
TRIP6	2954	0.807	0.452	0.779	0.492	0.707	0.410	0.317	0.539	0.137	0.116
TEK	1256	0.533	0.689	0.652	0.501	0.464	0.684	0.728	0.529	0.260	0.399
SPI1	1229	0.600	0.588	0.635	0.645	0.641	0.618	0.653	0.682	0.254	0.397
MFAP4	2358	0.764	0.797	0.764	0.763	0.757	0.753	0.765	0.799	0.150	0.402
KLK10	1272	0.832	0.829	0.853	0.772	0.769	0.616	0.769	0.813	0.120	0.097
SEMA3B	3894	0.727	0.829	0.794	0.804	0.785	0.797	0.805	0.798	0.102	0.186
SEMA3B	1886	0.714	0.843	0.821	0.784	0.816	0.840	0.819	0.839	0.208	0.153
SERPINE1	1037	0.820	0.852	0.863	0.855	0.840	0.839	0.847	0.882	0.340	0.392
MMP9	4160	0.810	0.848	0.874	0.878	0.858	0.847	0.854	0.871	0.108	0.114
IL16	1171	0.844	0.876	0.853	0.879	0.847	0.860	0.848	0.868	0.138	0.335
LAT	3562	0.846	0.872	0.859	0.840	0.870	0.865	0.857	0.888	0.073	0.087
CEACAM1	3171	0.842	0.882	0.879	0.800	0.869	0.883	0.864	0.916	0.121	0.237
SEPT9	6004	0.928	0.900	0.952	0.869	0.774	0.940	0.558	0.893	0.183	0.463
CASP10	121	0.884	0.899	0.880	0.918	0.901	0.894	0.913	0.899	0.071	0.153
PLAT	4991	0.811	0.905	0.914	0.912	0.911	0.914	0.920	0.911	0.342	0.329
AXL	1482	0.923	0.925	0.911	0.927	0.941	0.131	0.911	0.106	0.146	0.238
PSCA	5698	0.888	0.937	0.912	0.919	0.933	0.902	0.921	0.932	0.083	0.154
CARD15	4256	0.906	0.929	0.926	0.928	0.916	0.918	0.918	0.937	0.120	0.124
PDGFRB	2810	0.918	0.917	0.930	0.929	0.925	0.912	0.925	0.935	0.174	0.270
PTPRH	979	0.903	0.932	0.919	0.910	0.925	0.930	0.927	0.945	0.153	0.235
S100A4	1714	0.906	0.939	0.928	0.909	0.933	0.832	0.924	0.948	0.073	0.116
MFAP4	2363	0.912	0.936	0.929	0.932	0.922	0.913	0.930	0.932	0.171	0.449
PDGFRB	4153	0.919	0.936	0.929	0.931	0.937	0.928	0.936	0.951	0.094	0.109
P2RX7	2170	0.925	0.937	0.926	0.952	0.932	0.934	0.943	0.935	0.138	0.217
PECAM1	5686	0.895	0.950	0.936	0.940	0.931	0.943	0.943	0.946	0.224	0.283
VAMP8	4005	0.932	0.932	0.948	0.944	0.942	0.936	0.942	0.947	0.264	0.407
RARA	5118	0.886	0.942	0.943	0.952	0.942	0.927	0.943	0.946	0.201	0.528
CARD15	4255	0.914	0.947	0.940	0.949	0.935	0.936	0.947	0.945	0.149	0.318
VAMP8	2190	0.926	0.942	0.945	0.950	0.949	0.937	0.948	0.956	0.315	0.398
PECAM1	5123	0.908	0.948	0.947	0.954	0.945	0.949	0.949	0.947	0.255	0.324
MPO	2373	0.907	0.946	0.946	0.956	0.947	0.948	0.954	0.952	0.086	0.102
CD86	1766	0.953	0.955	0.956	0.949	0.945	0.912	0.947	0.950	0.116	0.323
STAT5A	5243	0.916	0.956	0.945	0.952	0.950	0.943	0.952	0.955	0.231	0.431
MPL	5750	0.934	0.958	0.953	0.956	0.951	0.950	0.955	0.953	0.260	0.364
TIE1	2941	0.922	0.958	0.954	0.960	0.952	0.950	0.954	0.958	0.236	0.393
IL10	1170	0.926	0.958	0.952	0.970	0.960	0.951	0.956	0.953	0.189	0.204
NOTCH4	913	0.935	0.917	0.955	0.958	0.953	0.956	0.957	0.955	0.119	0.348
EMR3	732	0.949	0.959	0.954	0.950	0.955	0.950	0.958	0.962	0.153	0.310
CSF3R	3463	0.939	0.958	0.948	0.969	0.956	0.955	0.962	0.966	0.176	0.427
MMP9	4158	0.952	0.959	0.962	0.956	0.958	0.957	0.965	0.962	0.236	0.443
AXL	5413	0.934	0.966	0.960	0.958	0.957	0.958	0.965	0.961	0.348	0.338
SPP1	5214	0.947	0.963	0.961	0.953	0.959	0.951	0.966	0.960	0.260	0.367
CASP10	3158	0.939	0.957	0.962	0.969	0.955	0.962	0.962	0.958	0.145	0.366
CD2	209	0.954	0.964	0.960	0.969	0.958	0.960	0.960	0.962	0.075	0.100
LTB4R	3582	0.950	0.963	0.957	0.970	0.961	0.958	0.966	0.961	0.161	0.278
TNFSF10	5375	0.955	0.964	0.962	0.975	0.962	0.963	0.962	0.956	0.145	0.164
IL8	754	0.955	0.963	0.965	0.980	0.963	0.958	0.967	0.961	0.095	0.132
EMR3	3222	0.913	0.962	0.959	0.966	0.965	0.958	0.968	0.965	0.160	0.350
LTB4R	1320	0.960	0.966	0.968	0.968	0.964	0.962	0.966	0.969	0.235	0.393
PADI4	3740	0.951	0.969	0.966	0.974	0.969	0.969	0.970	0.968	0.170	0.265
IL8	4225	0.964	0.973	0.970	0.979	0.971	0.970	0.974	0.969	0.169	0.265
AFF3	5475	0.961	0.971	0.971	0.977	0.969	0.969	0.973	0.971	0.093	0.085
WNT10B	5381	0.955	0.973	0.970	0.979	0.971	0.970	0.977	0.974	0.138	0.215
SERPINE1	5177	0.969	0.976	0.973	0.978	0.974	0.972	0.975	0.975	0.197	0.473

SPI1	5208	0.95	0.96	0.96	0.96	0.96	0.96	0.96	0.96	0.26	0.59
ABCC2	3072	0.96	0.96	0.96	0.96	0.95	0.95	0.96	0.95	0.95	0.95
WNT8B	6070	0.96	0.96	0.96	0.97	0.96	0.96	0.96	0.96	0.95	0.94
TNFRSF1A	5365	0.96	0.97	0.96	0.96	0.96	0.96	0.97	0.97	0.68	0.85
THPO	1276	0.94	0.96	0.96	0.97	0.96	0.95	0.96	0.96	0.61	0.75
EPHX1	2166	0.96	0.96	0.96	0.96	0.96	0.95	0.96	0.96	0.96	0.92
ZIM3	2205	0.97	0.97	0.96	0.96	0.96	0.95	0.96	0.96	0.96	0.94
SNCG	2022	0.95	0.96	0.96	0.97	0.96	0.95	0.96	0.96	0.26	0.64
CDH17	4283	0.96	0.96	0.96	0.97	0.96	0.96	0.96	0.96	0.91	0.95
CCL3	2868	0.94	0.96	0.96	0.96	0.96	0.94	0.97	0.96	0.64	0.44
NID1	5078	0.97	0.97	0.96	0.96	0.96	0.96	0.96	0.96	0.96	0.95
GPR116	2202	0.96	0.97	0.96	0.97	0.96	0.96	0.96	0.96	0.95	0.92
ACVR1	5829	0.96	0.96	0.96	0.97	0.96	0.95	0.96	0.96	0.76	0.82
MMP19	3693	0.96	0.96	0.96	0.96	0.96	0.96	0.96	0.96	0.9	0.92
ZIM3	4010	0.96	0.97	0.96	0.97	0.96	0.96	0.97	0.96	0.94	0.95
APOA1	66	0.97	0.97	0.96	0.97	0.96	0.95	0.96	0.96	0.96	0.95
DDR2	5516	0.97	0.96	0.96	0.98	0.97	0.95	0.96	0.95	0.93	0.93
ABCB4	2983	0.97	0.96	0.96	0.97	0.96	0.96	0.97	0.96	0.95	0.92
KRT5	2803	0.96	0.97	0.96	0.97	0.96	0.94	0.97	0.97	0.96	0.96
ABCB4	21	0.94	0.97	0.97	0.97	0.96	0.94	0.97	0.96	0.94	0.85
GABRA5	844	0.96	0.97	0.96	0.97	0.96	0.96	0.96	0.96	0.93	0.93
GLI2	609	0.96	0.96	0.97	0.97	0.96	0.96	0.97	0.96	0.95	0.96
GABRG3	3303	0.96	0.97	0.96	0.97	0.96	0.96	0.97	0.96	0.96	0.95
LCN2	5734	0.95	0.97	0.96	0.97	0.97	0.96	0.96	0.97	0.62	0.86
PRSS8	3828	0.96	0.98	0.97	0.95	0.96	0.96	0.97	0.97	0.97	0.96
TNF	2916	0.97	0.97	0.97	0.97	0.96	0.96	0.97	0.96	0.96	0.94
IAPP	5581	0.97	0.97	0.96	0.98	0.96	0.96	0.97	0.96	0.94	0.92
S100A4	6015	0.94	0.96	0.96	0.97	0.97	0.97	0.97	0.97	0.57	0.22
DCN	3592	0.97	0.97	0.96	0.98	0.96	0.96	0.97	0.96	0.95	0.93
LMO2	4123	0.95	0.97	0.96	0.97	0.97	0.97	0.97	0.97	0.26	0.67
IL13	4116	0.97	0.97	0.96	0.97	0.96	0.96	0.97	0.96	0.94	0.92
AGXT	2991	0.96	0.97	0.96	0.96	0.97	0.96	0.97	0.97	0.97	0.94
TMPRSS4	2951	0.97	0.97	0.96	0.96	0.96	0.96	0.97	0.97	0.96	0.96
PI3	1688	0.95	0.97	0.96	0.97	0.96	0.96	0.97	0.97	0.65	0.87
FOLR1	1599	0.97	0.97	0.96	0.97	0.96	0.96	0.97	0.97	0.95	0.95
ITK	2291	0.97	0.97	0.97	0.97	0.96	0.96	0.97	0.96	0.91	0.76
P2RX7	2173	0.97	0.97	0.96	0.97	0.97	0.96	0.97	0.96	0.96	0.96
H19	950	0.95	0.97	0.97	0.97	0.95	0.97	0.96	0.97	0.78	0.81
LCK	1646	0.97	0.98	0.97	0.96	0.96	0.97	0.97	0.97	0.92	0.84
MAS1	2733	0.97	0.97	0.97	0.97	0.96	0.96	0.97	0.96	0.95	0.95
TIMP3	2910	0.97	0.97	0.97	0.97	0.97	0.96	0.97	0.97	0.96	0.96
CDH17	5476	0.97	0.97	0.97	0.97	0.96	0.96	0.97	0.97	0.95	0.95
ACVR1	5799	0.97	0.97	0.96	0.98	0.97	0.93	0.97	0.96	0.95	0.95
TRIP6	2401	0.94	0.97	0.97	0.97	0.97	0.97	0.97	0.97	0.15	0.6
NOS2A	2838	0.97	0.97	0.97	0.97	0.97	0.96	0.97	0.97	0.86	0.88
SOD3	2044	0.97	0.97	0.97	0.98	0.97	0.97	0.97	0.96	0.79	0.86
IL10	1164	0.95	0.97	0.96	0.98	0.97	0.97	0.97	0.97	0.48	0.77
EGF	233	0.98	0.97	0.97	0.98	0.97	0.96	0.97	0.97	0.96	0.96
SERPINB2	1895	0.97	0.97	0.97	0.98	0.97	0.96	0.97	0.97	0.96	0.95
RUNX3	1869	0.96	0.97	0.97	0.97	0.97	0.96	0.97	0.97	0.89	0.67
MMP1	4304	0.97	0.97	0.97	0.98	0.97	0.97	0.97	0.96	0.96	0.96
EDNRB	715	0.97	0.97	0.97	0.98	0.97	0.97	0.98	0.97	0.95	0.93
EVI2A	398	0.96	0.97	0.97	0.98	0.97	0.97	0.97	0.97	0.59	0.49
SFTPA1	3913	0.98	0.97	0.97	0.97	0.96	0.97	0.98	0.96	0.97	0.96
TIMP3	2913	0.97	0.98	0.97	0.98	0.97	0.97	0.97	0.97	0.97	0.96
SRC	6030	0.97	0.97	0.97	0.97	0.97	0.97	0.97	0.97	0.97	0.97
FGR	5688	0.97	0.97	0.97	0.97	0.96	0.97	0.97	0.97	0.95	0.91
EDNRB	700	0.98	0.97	0.97	0.98	0.97	0.97	0.97	0.97	0.95	0.94
NID1	5082	0.97	0.98	0.97	0.98	0.97	0.97	0.97	0.97	0.96	0.94
TFF2	5257	0.97	0.97	0.97	0.98	0.97	0.97	0.98	0.97	0.97	0.96
CSF1R	210	0.96	0.97	0.97	0.98	0.97	0.97	0.98	0.97	0.6	0.79
CD1A	208	0.97	0.97	0.97	0.98	0.97	0.97	0.97	0.97	0.96	0.96
LMO2	2729	0.96	0.97	0.97	0.98	0.97	0.97	0.98	0.98	0.24	0.62
ACTG2	36	0.97	0.98	0.97	0.98	0.97	0.97	0.98	0.97	0.96	0.96
IL1A	674	0.98	0.98	0.97	0.98	0.97	0.97	0.98	0.96	0.97	0.96
TMPRSS4	2383	0.97	0.98	0.97	0.98	0.97	0.97	0.98	0.97	0.86	0.66
ZNFN1A1	6078	0.98	0.98	0.97	0.98	0.97	0.97	0.98	0.97	0.97	0.97
AGXT	50	0.97	0.98	0.98	0.98	0.98	0.98	0.98	0.98	0.78	0.83
GPR116	2995	0.98	0.98	0.98	0.98	0.97	0.97	0.98	0.97	0.97	0.95
GNG7	2739	0.98	0.98	0.97	0.98	0.98	0.97	0.98	0.98	0.97	0.97
C4B	171	0.98	0.98	0.97	0.96	0.98	0.97	0.98	0.98	0.95	0.94
BLK	162	0.97	0.98	0.98	0.98	0.98	0.98	0.98	0.98	0.95	0.92
ITK	2794	0.98	0.98	0.98	0.98	0.97	0.97	0.98	0.97	0.93	0.78
UGT1A7	2413	0.98	0.98	0.97	0.98	0.97	0.97	0.98	0.98	0.96	0.96
ZIM3	2204	0.98	0.98	0.97	0.98	0.98	0.98	0.98	0.98	0.97	0.97
AFF3	5371	0.98	0.98	0.98	0.98	0.98	0.98	0.98	0.98	0.97	0.97
MMP1	4136	0.98	0.98	0.98	0.98	0.98	0.98	0.98	0.98	0.97	0.97
CCL3	1959	0.98	0.98	0.98	0.98	0.98	0.98	0.99	0.98	0.96	0.92
PWCR1	3846	0.98	0.98	0.98	0.98	0.98	0.98	0.98	0.98	0.97	0.97
